# Evaluating Reference Ages for Selecting Prosthesis Types for Heart Valve Replacement in Korea

**DOI:** 10.1001/jamanetworkopen.2023.14671

**Published:** 2023-05-22

**Authors:** Sung Jun Park, You Jung Ok, Ho Jin Kim, Ye-Jee Kim, Seonok Kim, Jung-Min Ahn, Dae-Hee Kim, Jae-Sung Choi, Joon Bum Kim

**Affiliations:** 1 Department of Thoracic and Cardiovascular Surgery, Severance Cardiovascular Hospital, Yonsei University College of Medicine, Seoul, Korea; 2 Department of Thoracic and Cardiovascular Surgery, SMG-SNU Boramae Medical Center, Seoul National University College of Medicine, Seoul, Korea; 3 Department of Thoracic and Cardiovascular Surgery, Asan Medical Center, University of Ulsan College of Medicine, Seoul, Korea; 4 Department of Clinical Epidemiology and Biostatistics, Asan Medical Center, University of Ulsan College of Medicine, Seoul, Korea; 5 Divison of Cardiology, Asan Medical Center, University of Ulsan College of Medicine, Seoul, Korea

## Abstract

**Importance:**

Although a patient’s age may be the only objective figure that can be used as a reference indicator in selecting the type of prosthesis in heart valve surgery, different clinical guidelines use different age criteria.

**Objective:**

To explore the age-associated survival-hazard functions associated with prosthesis type in aortic valve replacement (AVR) and mitral valve replacement (MVR).

**Design, Setting, and Participants:**

This cohort study compared the long-term outcomes associated with mechanical and biologic prostheses in AVR and MVR according to recipient’s age using a nationwide administrative data from the Korean National Health Insurance Service. To reduce the potential treatment-selection bias between mechanical and biologic prostheses, the inverse-probability-of-treatment-weighting method was used. Participants included patients who underwent AVR or MVR in Korea between 2003 and 2018. Statistical analysis was performed between March 2022 and March 2023.

**Exposures:**

AVR, MVR, or both AVR and MVR with mechanical or biologic prosthesis.

**Main Outcomes and Measures:**

The primary end point was all-cause mortality after receiving prosthetic valves. The secondary end points were the valve-related events, including the incidence of reoperation, systemic thromboembolism, and major bleeding.

**Results:**

Of the total of 24 347 patients (mean [SD] age, 62.5 [7.3] years; 11 947 [49.1%] men) included in this study, 11 993 received AVR, 8911 received MVR, and 3470 received both AVR and MVR simultaneously. Following AVR, bioprosthesis was associated with significantly greater risks of mortality than mechanical prosthesis in patients younger than 55 years (adjusted hazard ratio [aHR], 2.18; 95% CI, 1.32-3.63; *P* = .002) and in those aged 55 to 64 years (aHR, 1.29; 95% CI, 1.02-1.63; *P* = .04), but the risk of mortality reversed in patients aged 65 years or older (aHR, 0.77; 95% CI, 0.66-0.90; *P* = .001). For MVR, the risk of mortality was also greater with bioprosthesis in patients aged 55 to 69 years (aHR, 1.22; 95% CI, 1.04-1.44; *P* = .02), but there was no difference for patients aged 70 years or older (aHR, 1.06; 95% CI, 0.79-1.42; *P* = .69). The risk of reoperation was consistently higher with bioprosthesis, regardless of valve position, in all age strata (eg, MVR among patients aged 55-69 years: aHR, 7.75; 95% CI, 5.14-11.69; *P* < .001); however, the risks of thromboembolism and bleeding were higher in patients aged 65 years and older after mechanical AVR (thromboembolism: aHR, 0.55; 95% CI, 0.41-0.73; *P* < .001; bleeding: aHR, 0.39; 95% CI, 0.25-0.60; *P* < .001), with no differences after MVR in any age strata.

**Conclusions and Relevance:**

In this nationwide cohort study, the long-term survival benefit associated with mechanical prosthesis vs bioprosthesis persisted until age 65 years in AVR and age 70 years in MVR.

## Key Points

**Question:**

Which type of prosthesis is associated with the best outcomes by age of the recipient undergoing aortic or mitral valve replacement?

**Findings:**

This cohort study of 24 347 patients who underwent aortic or mitral valve replacement compared the long-term outcomes associated with mechanical and bioprostheses according to the recipient’s age. The mechanical prosthesis was associated with a survival benefit over bioprosthesis, and the benefit was maintained in patients up to age 65 years for replacements in the aortic position and age 70 years for replacements in the mitral position.

**Meaning:**

The findings of this study may encourage health care practitioners to adopt a more conservative approach in choosing a prosthesis type and provide information for better shared decision-making for both patients and physicians.

## Teaser

This cohort study compares the long-term outcomes associated with mechanical and biologic prostheses in aortic valve replacement and mitral valve replacement according to recipient’s age using a nationwide administrative data in Korea.

## Introduction

Prosthetic valve replacement is the gold standard for the treatment of severe, symptomatic valvular heart disease.^1^ The choice of prosthesis type (biologic vs mechanical) is influenced by several factors, including the patient’s age, lifestyle, and preference, and the trade-off dynamics between the risks of reintervention and life-long anticoagulation.^2,3,4^ The individual patient’s values and preferences are important factors in the shared decision-making process for this choice; however, the patient’s age may be the only objective figure that health care practitioners can present as a reference indicator among several influential factors.

The latest US and European guidelines for valvular heart disease have suggested an age criterion for biologic or mechanical prosthesis according to valve position (aortic or mitral).^5,6^ Owing to the lack of sufficiently powered randomized trials, large registry-type observational studies have been the major bases for evidence for these guidelines.^7,8,9,10^ Despite the substantial overlap of evidence bases, these 2 guidelines have taken different positions regarding the selection of prosthesis type, with a more forward stance on the use of bioprosthesis in the American College of Cardiology and American Heart Association (ACC/AHA) guidelines.^5,6^ The ACC/AHA guidelines’ flexibly individualize the choice of either mechanical or bioprosthetic aortic valve replacement (AVR) for patients aged 50 to 65 years, whereas the guidelines from the European Society of Cardiology and European Association for Cardio-Thoracic Surgery recommend mechanical prosthesis in AVR for patients up to age 60 years. The age criterion disaccord between these landmark guidelines leaves this issue uncertain.

The accumulation of experiences based on a sufficiently large cohort may help shape a balanced perspective on this issue. Therefore, this study aimed to compare the nationwide outcomes of mechanical vs bioprosthesis for AVR or mitral valve replacement (MVR), using the national administrative database of the Korean National Health Insurance Service (NHIS) linked with real-time vital status information derived from Statistics Korea.^11,12,13^

## Methods

This cohort study was approved by the institutional review board of Asan Medical Center, Seoul, Korea, and the study was deemed exempt from requiring individual patients’ consent because all of the data from the NHIS were anonymized. We followed the Strengthening the Reporting of Observational Studies in Epidemiology (STROBE) reporting guideline.

### Data Sources

The NHIS, Korea’s national, mandatory health care insurance system, is a single-payer program covering 100% of the Korean population (52 million in 2019).^12^ The NHIS has established a public nationwide claims database (National Health Information Database) that includes sociodemographics, vital statistics, national health screening data, and health care data with diagnosis, procedures, and prescriptions for all patients.^11^ Diagnoses are coded according to the *International Statistical Classification of Diseases and Related Health Problems, Tenth Revision *(*ICD-10*). Complete long-term follow-up of all the patient was possible by virtue of the NHIS single-payer system.^14^

All baseline comorbidities of this cohort were identified by extracting *ICD-10* codes, which were recorded twice or more individually within 1 year of surgery (eTable 1 in Supplement 1). A history of operative profiles was also identified by extracting the conforming NHIS claim codes for each patient (eTable 2 in Supplement 1). We included the cumulative hospital volume for cardiac surgery during the study period (2003-2018), and health screening data for inclusive comparisons. Charlson comorbidity index (CCI) and congestive heart failure, hypertension, age at least 75 years (doubled), diabetes, stroke (doubled), vascular disease, age 65 to 74 years, and sex category (female) (CHA_2_DS_2_-VASc) score were assessed from the baseline comorbidities.

### Study Population

This study included patients who underwent AVR or MVR between January 2003 and December 2018. The exclusion criteria for patients were repeat AVR or MVR surgery, concomitant pulmonic or tricuspid valve replacement, concomitant aorta surgery, preoperative mechanical circulatory support or mechanical ventilation, concomitant aortic or mitral valve repair, simultaneous AVR and MVR with different types of prostheses, and concomitant cardiac tumor surgery (eFigure 1 in Supplement 1). This study assessed patients who received AVR or MVR as well as those who received both AVR and MVR simultaneously (double valve replacement [DVR]).

An age-stratified analysis was conducted. The cutoff for age strata of AVR or MVR was chosen from the exploratory analysis examining the age-associated relative hazards of bioprosthesis compared with mechanical prosthesis. For AVR and DVR, patients were subcategorized by age into 40 to 54 years, 55 to 64 years, and 65 to 79 years; for MVR, they were subcategorized into 40 to 54 years, 55 to 69 years, and 70 to 79 years.

### Study End Points

The primary outcome was all-cause mortality after prosthetic valve replacement. The secondary outcomes were cardiovascular death and the valve-related events, including the reoperation, systemic thromboembolism (including ischemic stroke), and major bleeding (including hemorrhagic stroke) (eTable 3 in Supplement 1).

Data on vital status and cause of death (COD) were compiled and linked with data from Statistics Korea through 1-by-1 match-up using personal ID numbers. Statistics Korea annually collects vital status and COD information from death certificates and classifies COD according to the Korean Standard Classification of Diseases and Causes of Death, based on the *ICD-10*. Cardiovascular death was defined as *ICD-10* codes for diseases of the circulatory system (I00-99). Ischemic stroke event was defined as having both an *ICD-10* diagnostic code and an NHIS claims code for brain image studies (computed tomography or magnetic resonance imaging) at the hospital. Major bleeding was defined as hemorrhagic stroke diagnosed with brain image studies, gastrointestinal bleeding, or hemorrhagic events that occurred in unclassified sites (eg, extracranial, intraocular, intraarticular, and hemothorax) requiring hospitalization.

### Statistical Analysis

Categorical variables were compared using the χ^2^ test or Fisher exact test and are presented as frequencies and percentages. Continuous variables, expressed as mean and SD, were compared using the *t* test.

To reduce the potential treatment-selection bias, the inverse-probability-of-treatment-weighting (IPTW) method was used based on the propensity score incorporating all the baseline variables. With that technique, weights for patients receiving a mechanical prosthesis were the inverse of (1 − propensity score), and weights for patients receiving a bioprosthetic were the inverse of propensity score. Propensity scores were estimated separately using multiple logistic-regression analysis for each age strata in each of the 3 populations (AVR, MVR, and DVR). The IPTW based on the stabilized weight was truncated to values between the 95th and 99th percentiles. Using the estimated weights, we examined the similarity between the mechanical and bioprosthetic groups by calculating standardized mean differences (SMDs). Variables with SMD greater than 0.2, except for Health Screening Data, were additionally adjusted to compare the risk of primary and secondary outcomes (MVR: year of surgery; DVR: CCI and cumulative hospital volume for cardiac surgery).

To assess the age-dependent associations of prosthesis type with mortality, a Cox proportional-hazards model was fit with the use of an interaction term for the age and prosthesis types in the IPTW-adjusted cohort. Linear and natural cubic spline models with the number of knots (2, 3, 4, or 5 knots) function were considered and compared based on the Akaike information criterion. The natural cubic spline model with 2 knots was chosen as the most suitable.

Age-stratified analyses were conducted based on the respective age cutoffs for AVR (65 years) and MVR (70 years) in these exploratory models. After adjustment with the IPTW-method, a Cox proportional hazard model with robust SEs was used to compare the risk of primary and secondary outcomes between the mechanical and bioprosthetic groups. The proportional hazards assumption was assessed using the Schoenfeld residual, which yielded no evidence to suggest rejecting the assumption. The secondary outcomes were also evaluated after adjustment with the IPTW method, with all-cause mortality as a competing risk.^15^ Subdistribution hazards were estimated with the Fine and Gray method.

Subgroup analyses were performed across various subgroups to compare the outcomes between the mechanical and bioprosthesis. For these comparisons, the interaction between prosthesis types and each subgroup was evaluated in IPTW-adjusted cohorts. All reported *P* values were 2-tailed, and *P* < .05 was considered statistically significant. We used R software version 4.0.3 (R Project for Statistical Computing) and SAS Enterprise Guide software version 7.1 (SAS Institute) for statistical analyses. Statistical analysis was performed between March 2022 and March 2023.

## Results

### Patient Characteristics

Among 39 851 patients who underwent at least 1 left-sided prosthetic heart valve replacement between 2003 and 2018 in South Korea, a total of 24 374 patients (mean [SD] age, 62.5 [7.3] years; 11 947 [49.1%] men) were included in this study after applying the exclusion criteria (eFigure 1 in Supplement 1). A total of 11 993 patients underwent AVR, 8911 patients underwent MVR, and 3470 patients received DVR. The types of valves used in this study are listed in eTable 4 in Supplement 1. Distributions of patients undergoing valve replacements by age are shown in eFigure 2 in Supplement 1.

Baseline and operative characteristics of patients are summarized in Table 1. At baseline, recipients of bioprosthesis were older and had more comorbidities (higher CCI) than recipients of mechanical prosthesis (Table 1). The distributions of propensity scores are presented in eFigure 3 in Supplement 1. After adjustment with the IPTW method, most of the covariates in the cases of AVR and MVR were well-balanced between the groups, throughout the all age strata (eTables 5-10 in Supplement 1). Across all subpopulations of AVR and MVR, the only variables with adjusted SMD greater than 0.20 were years of surgery and level of hospital in patients aged 40 to 54 years who underwent MVR. However, in the patients who underwent DVR, there were limitations in obtaining an evenly balanced cohort even after adjustment because of the relatively small cohort and eccentric use of prosthesis type, except for the age strata of patients aged 65 years or older (eTables 11-13 in Supplement 1).

**Table 1.** Baseline and Operative Characteristics According to the Types of Prostheses, Before Inverse Probability Weighting

Variable	AVR prosthesis			MVR prosthesis			DVR prosthesis		
	No. (%)		SMD	No. (%)		SMD	No. (%)		SMD
	Mechanical (n = 4825)	Biological (n = 7168)		Mechanical (n = 5957)	Biological (n = 2954)		Mechanical (n = 2463)	Biological (n = 1007)	
Age, mean (SD), y	57.5 (8.0)	70.7 (6.0)	1.857	55.2 (8.0)	68.7 (7.2)	1.770	55.0 (7.8)	69.3 (6.8)	1.950
Sex									
Female	1691 (35.0)	3200 (44.6)	0.197	3507 (58.9)	1985 (67.2)	0.173	1395 (56.6)	595 (59.1)	0.050
Male	3134 (65.0)	3968 (55.4)	0.197	2450 (41.1)	969 (48.8)	0.173	1068 (43.4)	412 (40.9)	0.050
Baseline comorbidities									
Atrial fibrillation	277 (5.7)	456 (6.4)	0.026	2242 (37.6)	12.4 (40.8)	0.064	870 (35.3)	363 (36.0)	0.015
Hypertension	2563 (53.1)	5154 (71.9)	0.396	2909 (48.8)	1954 (66.1)	0.356	1270 (51.6)	665 (66.0)	0.297
Diabetes	933 (19.3)	2267 (31.6)	0.285	818 (13.7)	811 (27.5)	0.344	313 (12.7)	237 (23.5)	0.284
Dyslipidemia	768 (15.9)	1862 (26.0)	0.249	722 (12.1)	533 (18.0)	0.166	284 (11.5)	189 (18.8)	0.203
CKD	200 (4.1)	406 (5.7)	0.070	126 (2.1)	148 (5.0)	0.157	65 (2.6)	50 (5.0)	0.122
Dialysis	152 (3.2)	235 (3.3)	0.007	66 (1.1)	80 (2.7)	0.117	41 (1.7)	33 (3.1)	0.104
Stroke, TIA, or SE	373 (7.7)	954 (13.3)	0.183	841 (14.1)	530 (17.9)	0.104	290 (11.8)	161 (16.0)	0.122
Ischemic heart disease	1611 (33.4)	3233 (45.1)	0.242	1215 (20.4)	917 (31.0)	0.245	561 (22.8)	319 (31.7)	0.201
Myocardial infarction	130 (2.7)	290 (4.0)	0.075	149 (2.5)	109 (3.7)	0.069	60 (2.4)	31 (3.1)	0.039
Previous PCI	168 (3.5)	550 (7.7)	0.183	92 (1.5)	126 (4.3)	0.163	37 (1.5)	42 (4.2)	0.161
Congestive heart failure	1213 (25.1)	2285 (31.9)	0.150	2365 (39.7)	1519 (51.4)	0.237	1042 (42.3)	504 (50.0)	0.156
Anemia	438 (9.1)	909 (12.7)	0.116	486 (8.2)	403 (13.6)	0.177	203 (8,2)	134(13.3)	0.164
COPD	160 (3.3)	520 (7.3)	0.177	180 (3.0)	216 7.3)	0.195	67 (2.7)	70 (7.0)	0.198
Asthma	518 (10.7)	1253 (17.5)	0.195	817 (13.7)	605 (20.5)	0.180	288 (11.7)	224 (22.2)	0.284
Peripheral vascular disease	256 (5.3)	629 (8.8)	0.136	263 (4.4)	209 (7.1)	0.115	106 (4.3)	69 (6.9)	0.111
Previous cardiac surgery	19 (0.4)	43 (0.6)	0.029	23 (0.4)	22 (0.7)	0.048	3 (0.1)	6 (0.6)	0.079
Previous cancer	227 (4.7)	683 (9.5)	0.188	200 (3.4)	209 (7.1)	0.168	68 (2.8)	70 (7.0)	0.196
CCI									
0	1557 (32.3)	1232 (17.2)	0.459	1617 (27.1)	376 (12.7)	0.507	686 (27.9)	168 (16.7)	0.474
1	1293 (26.8)	1549 (21.6)	0.399	1700 (28.5)	668 (22.6)	0.488	781 (31.7)	225 (22.3)	0.449
2	837 (17.3)	1479 (20.6)		1196 (20.1)	628 (21.3)		463 (18.8)	210 (20.9)	
≥3	728 (15.1)	1761 (24.6)		1051 (17.6)	798 (27.0)		395 (16.0)	260 (25.8)	
≥5	410 (8.5)	1147 (16.0)		393 (6.6)	484 (16.4)		138 (5.6)	144 (14.3)	
Year of surgery									
2002-2005	1024 (21.2)	701 (9.8)	0.390	1604 (26.9)	481 (16.3)	0.286	734 (29.8)	173 (17.2)	0.365
2006-2009	1125 (23.3)	1369 (19.1)		1461 (24.5)	720 (24.4)		628 (25.5)	220 (21.8)	
2010-2013	1255 (26.0)	2001 (27.9)		1325 (22.2)	711 (24.1)		509 (20.7)	252 (25.0)	
2014-2018	1421 (29.5)	3097 (43.2)		1567 (26.3)	1042 (35.3)		592 (24.0)	362 (35.9)	
Level of hospital									
Tertiary general^a^	3855 (79.9)	5327 (74.3)	0.133	4557 (76.5)	2138 (72.4)	0.095	1978 (80.3)	758 (75.3)	0.121
General	970 (20.1)	1841 (25.7)		1400 (23.5)	816 (27.6)		485 (19.7)	249 (24.7)	
Cumulative hospital volume for AVR, No. per year									
<250	1255 (26.0)	1923 (26.8)	0.156	NA	NA	NA	511 (20.7)	278 (27.6)	0.255
250-999	1313 (27.2)	2009 (28.0)		NA	NA		668 (27.1)	298 (29.6)	
1000-2999	1275 (26.4)	2181 (30.4)		NA	NA		841 (34.1)	327 (32.5)	
≥3000	982 (20.4)	1055 (14.7)		NA	NA		443 (18.0)	104 (10.3)	
Cumulative hospital volume for MVR, No. per year									
<250	NA	NA	NA	1789 (30.0)	1014 (34.3)	0.291	618 (25.1)	340 (33.8)	0.211
250 to 1000	NA	NA		1549 (26.0)	968 (32.8)		561 (22.8)	236 (23.4)	
1000 to 3000	NA	NA		1628 (27.3)	733 (24.8)		1284 (52.1)	431 (42.8)	
≥3000	NA	NA		991 (16.6)	239 (8.1)		0	0	
Infective endocarditis	697 (14.4)	692 (9.7)	0.148	775 (13.0)	484 (16.4)	0.095	363 (14.7)	205 (20.4)	0.148
Congestive heart failure	1242 (25.7)	2017 (28.1)	0.054	2145 (36.0)	1275 (43.2)	0.147	945 (38.4)	462 (45.9)	0.153
Mode of valve disease									
Aortic stenosis	1493 (30.9)	3649 (50.9)	0.464	NA	NA	NA	469 (19.0)	265 (26.3)	0.183
Aortic regurgitation	1399 (29.0)	1030 (14.4)		NA	NA		709 (28.8)	269 (26.7)	
Combined	1792 (37.1)	2325 (32.4)		NA	NA		934 (37.9)	327 (32.5)	
Unspecified	141 (2.9)	164 (2.3)		NA	NA		351 (14.3)	146 (14.5)	
Mitral stenosis	NA	NA	NA	3120 (52.4)	1563 (52.9)	0.149	1264 (51.3)	528 (52.4)	0.182
Mitral regurgitation	NA	NA		180 (3.0)	112 (3.8)		78 (3.2)	42 (4.2)	
Combined	NA	NA		2424 (40.7)	1078 (36.5)		901 (36.6)	303 (30.1)	
Unspecified	NA	NA		233 (3.9)	201 (6.8)		220 (8.9)	134 (13.3)	
Concomitant procedure									
Tricuspid valve repair	164 (3.4)	181 (2.5)	0.052	2450 (41.1)	1299 (44.0)	0.058	959 (38.9)	365 (36.2)	0.056
Coronary arterial bypass grafting	450 (9.3)	1275 (17.8)	0.249	269 (4.5)	298 (10.1)	0.215	67 (2.7)	62 (2.7)	0.167
Surgical ablation for atrial fibrillation	207 (4.3)	358 (5.0)	0.033	2622 (44.0)	1237 (41.9)	0.043	973 (39.5)	367 (36.4)	0.063
BMI									
Mean (SD)	24.4 (3.2)	24.3 (3.3)	NA	23.6 (3.2)	23.5 (3.3)	NA	23.3 (3.0)	23.2 (3.2)	NA
<18.5	49 (1.0)	138 (1.9)	0.031	124 (2.1)	75 (2.5)	0.028	59 (2.4)	40 (4.0)	0.009
≥18.5 to <23	1046 (21.7)	1636 (22.8)		1419 (23.8)	755 (25.6)		597 (24.2)	248 (24.6)	
≥23 to <25	818 (17.0)	1266 (17.7)		824 (13.8)	430 (14.6)		381 (15.5)	175 (17.4)	
≥25 to <30	1168 (24.2)	1840 (25.7)		966 (16.2)	500 (16.9)		346 (14.0)	133 (13.2)	
≥30	164 (3.4)	261 (3.6)		119 (2.0)	64 (2.2)		30 (1.2)	16 (1.6)	
Not available	1580 (32.7)	2027 (28.3)		2505 (42.1)	1130 (38.3)		1050 (42.6)	395 (39.2)	
Blood pressure, mm Hg									
Systolic									
Mean (SD)	126.5 (17.0	129.9 (17.3)	0.171	119.3 (16.2)	124.3 (16.8)	0.243	119.9 (15.8)	124.8 (17.9)	0.243
<120	1024 (21.2)	1280 (17.9)	0.200	1712 (28.7)	658 (22.3)	0.305	672 (27.3)	209 (20.8)	0.292
≥120 to <140	1536 (31.8)	2465 (34.4)		1375 (23.1)	836 (28.3)		587 (23.8)	291 (28.9)	
≥140	685 (14.2)	1381 (19.3)		365 (6.1)	326 (11.0)		153 (6.2)	112 (11.1)	
Not available	1580 (32,7)	2042 (28.5)		2505 (42.1)	1134 (38.4)		1051 (42.7)	395 (39.2)	
Diastolic									
Mean (SD)	78.89 (11.1)	75.8 (11.1)	0.0008	74.3 (11.2)	75.3 (11.1)	0.089	72.4 (10.6)	73.4 (10.9)	0.090
<80	1762 (36.5)	2900 (40.5)	0.105	2191 (36.8)	1061 (35.9)	0.115	961 (39.0)	390 (38.7)	0.108
≥80 to <90	1074 (22.3)	1554 (21.7)		937 (15.7)	544 (18.4)		354 (14.4)	165 (16.4)	
≥90	409 (8.5)	672 (9.4)		324 (5.4)	215 (7.3)		97 (3.9)	57 (5.7)	
Not available	1580 (32.7)	2042 (28.5)		2505 (42.1)	1134 (38.4)		1051 (42.7)	395 (39.2)	
Smoking									
Never smoker	1771 (36.7)	3344 (46.7)	0.224	2383 (40.0)	1426 (48.3)	0.195	973 (39.5)	459 (45.6)	0.142
Previous smoker	717 (14.9)	1000 (14.0)		455 (7.6)	191 (6.5)		211 (8.6)	80 (7.9)	
Current smoker	712 (14.8)	704 (9.8)		541 (9.1)	164 (5.6)		203 (8.2)	58 (5.8)	
Not available	1625 (33.7)	2120 (29.6)		2578 (43.3)	1173 (39.7)		1076 (43.7)	410 (40.7)	
Alcohol use									
None	1358 (28.1)	3072 (42.9)	0.337	1560 (26.2)	1092 (38.0)	0.254	608 (24.7)	371 (36.8)	0.303
Mild to moderate	1662 (34.4)	1664 (23.2)		1712 (28.7)	621 (21.0)		729 (29.6)	200 (19.9)	
Heavy	187 (3.9)	314 (4.4)		114 (1.9)	64 (2.2)		53 (2.2)	27 (2.7)	
Not available	1618 (33.5)	2118 (29.5)		2571 (43.2)	1177 (39.8)		1073 (43.6)	409 (40.6)	
Creatinine, mg/dL									
≤1.5	2157 (44.7)	3954 (55.2)	0.247	2191 (36.8)	1254 (42.5)	0.176	845 (34.3)	433 (43.0)	0.196
>1.5	84 (1.7)	229 (3.2)		56 (0.9)	73 (2.5)		26 (1.1)	18 (1.8)	
Not available	2584 (53.6)	2985 (41.6)		3710 (62.3)	1627 (55.1)		1592 (64.6)	556 (55.2)	
eGFR, mL/min/1.73 m^2^									
≥60	1586 (32.9)	2595 (36.2)	0.292	1529 (25.7)	753 (25.5)	0.274	597 (24.2)	266 (26.4)	0.281
<60	211 (4.4)	814 (11.4)		229 (3.8)	320 (10.8)		101 (4.1)	111 (11.0)	
Not available	3028 (62.8)	3759 (52.4)		4199 (70.5)	1881 (63.7)		1765 (71.7)	630 (62.6)	

Abbreviations: AVR, aortic valve replacement; BMI, body mass index (calculated as weight in kilograms divided by height in meters squared); CCI, Charlson Comorbidity Index; CKD, chronic kidney disease; COPD, chronic obstructive pulmonary disease; DVR, double valve replacement; eGFR, estimated glomerular filtration rate; MVR, mitral valve replacement; PCI, percutaneous coronary intervention; SE, systemic embolization; SMD, standardized mean difference; TIA, transient ischemic attack.

SI conversion factor: To convert creatinine to micromoles per liter, multiply by 76.25.

^a^ Designated and certificated by the Ministry of Health and Welfare.

The number of patients who received bioprostheses increased throughout the study period regardless of valve position. For AVR, the use of bioprostheses increased from 9.8% of patients in the first quartile (2002-2005) to 43.2% of patients in the last quartile (2014-2018) (*P* < .001). For MVR, the proportion of bioprosthesis increased from 16.3% of patients in the first quartile to 35.3% of patients in the last quartile (*P* < .001).

### Mortality After Valve Replacement Surgery

**Figure 1.**
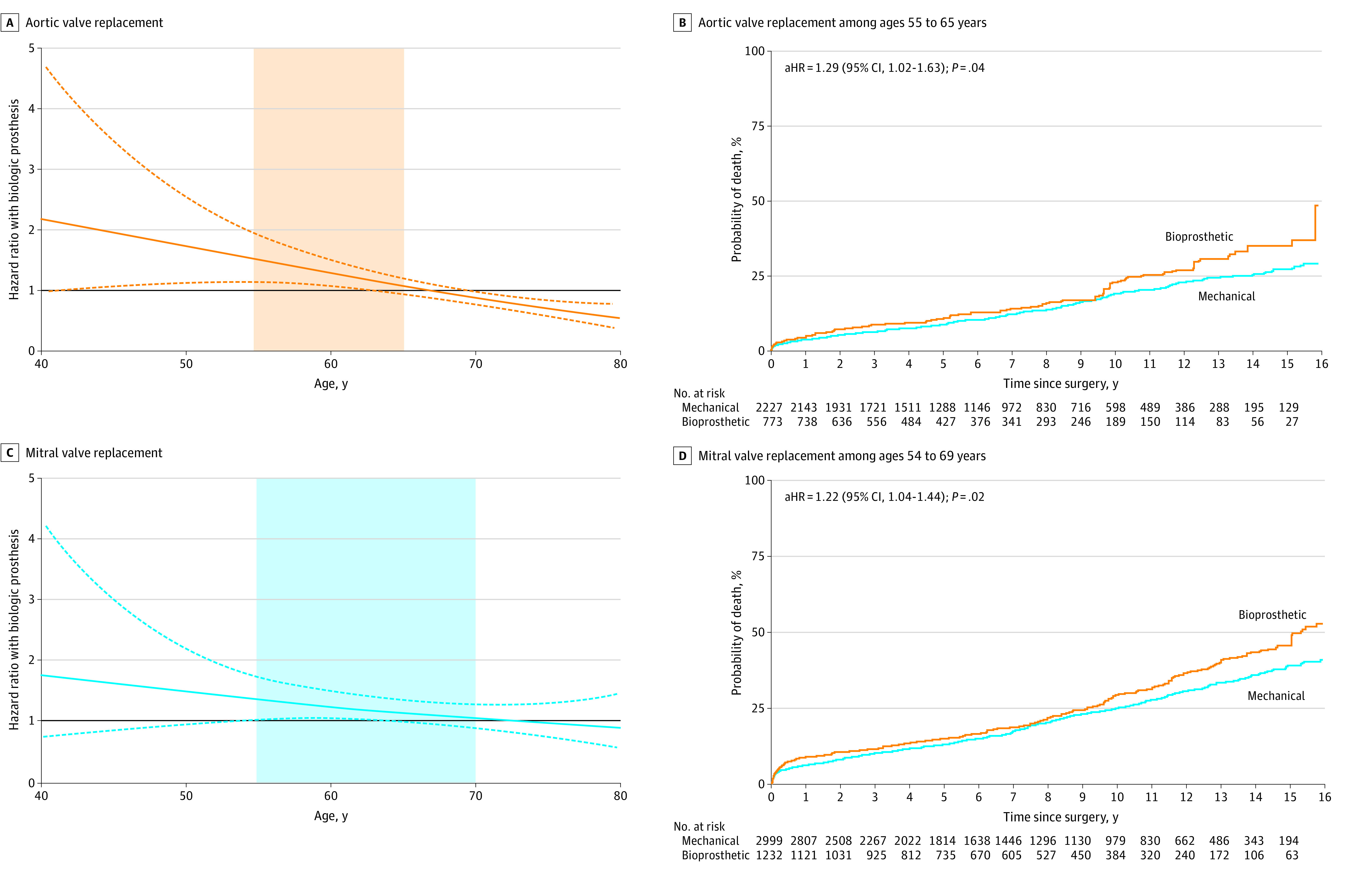
Age-Associated Relative Hazards of Mortality With Bioprosthesis Compared With Mechanical Prosthesis and Adjusted Risks of Mortality in Middle Age Strata A and C, Central solid lines indicate the adjusted hazard ratio; dashed lines, 95% CIs. The horizontal line at 1.00 denotes no difference between bioprosthesis vs mechanical prosthesis; shading, age 55 to 64 years in aortic valve replacement and age 55to 69 years in mitral valve replacement, which correspond to the survival curves in the B and D, respectively. It corresponds to the survival curve in the right part of this figure.

The spline curves of Figure 1 display the age-associated relative hazards of mortality for bioprosthesis compared with mechanical prosthesis in each valve position. Patient age was examined as a continuous variable in the IPTW-adjusted cohort. The central adjusted hazard ratio (aHR) line nearly touches the baseline (HR = 1.0) at approximately age 65 years for AVR (Figure 1A) and approximately age 70 years for MVR (Figure 1B), suggesting that the survival benefit associated with mechanical prosthesis may persist to a higher age in MVR than AVR.

**Figure 2.**
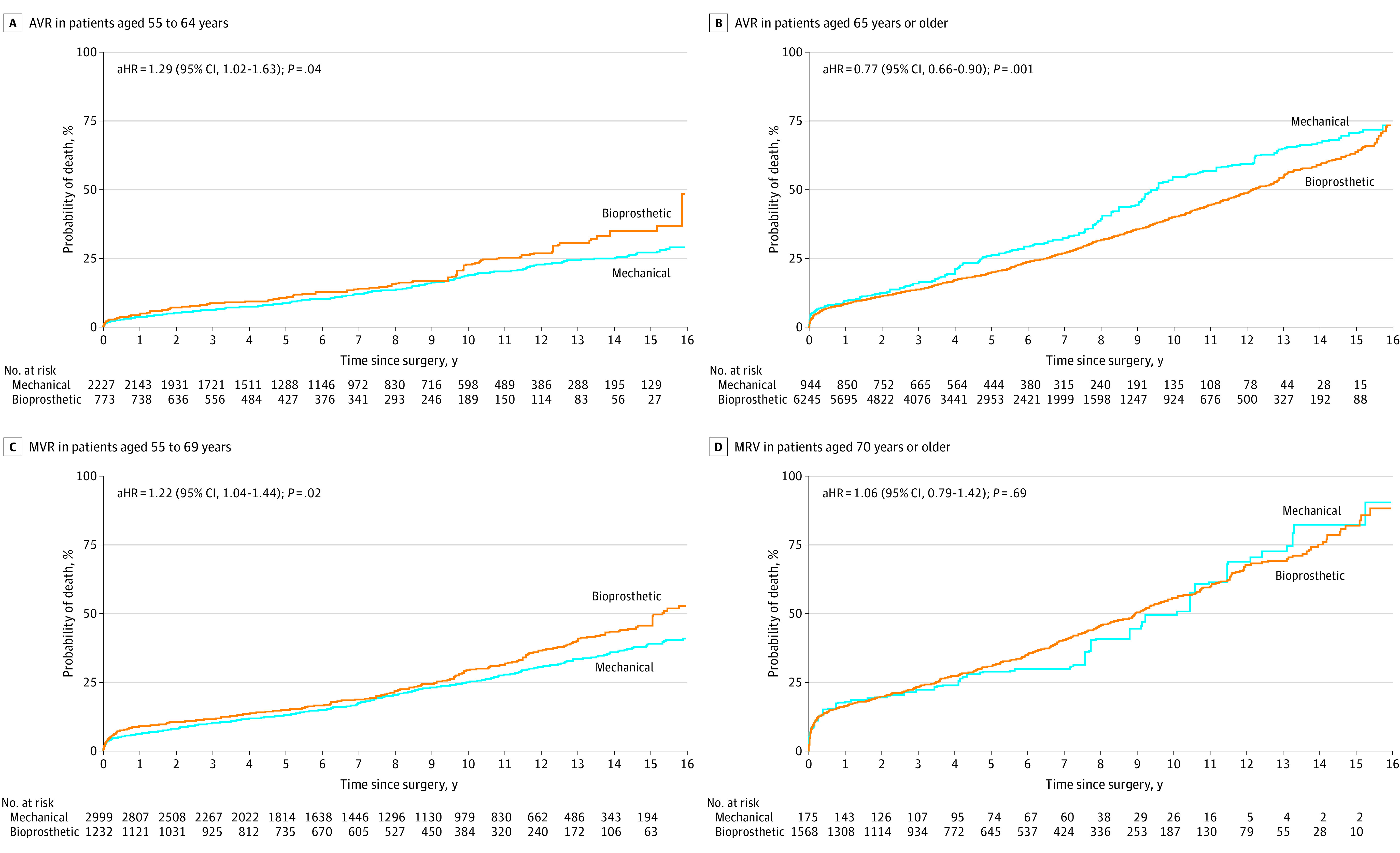
Adjusted Risks of Mortality Associated With Bioprosthesis According to Age Strata aHR, indicates adjusted hazard ratio; AVR aortic valve replacement; MVR, mitral valve replacement.

Similarly, in the stratified analysis of AVR according to age strata, the risks of long-term mortality were significantly higher with bioprosthesis in patients aged 40 to 54 years (aHR, 2.18; 95% CI, 1.32-3.63; *P* = .002) and in patients aged 55 to 64 years (aHR, 1.29; 95% CI, 1.02-1.63; *P* = .04), whereas bioprosthesis was associated with a lower mortality in patients aged 65 to 79 years (aHR, 0.77; 95% CI, 0.66-0.90; *P* = .001) (Table 2 and Figure 2A; eFigure 4 in Supplement 1).

**Table 2.** Comparative Outcomes Associated With Mechanical vs Bioprosthetic Aortic Valve Replacement Using Competing-Risk Analysis

Outcome	Unadjusted		IPTW-adjusted			
	HR (95% CI)	*P* value	Mechanical prosthesis, No.	Biological prosthesis, No.	aHR (95% CI)	*P* value
Age <55 y (n = 1804)^a^						
Death	2.79 (1.91-4.07)	<.001	186	30	2.19 (1.32-3.63)	.002
Cardiovascular death	3.23 (2.01-5.20)	<.001	93	15	1.99 (1.07-3.68)	.03
Noncardiovascular death	1.82 (0.97-3.42)	.06	93	15	2.17 (0.97-4.83)	.06
Valve-related events						
Reoperation	4.58 (2.32-9.04)	<.001	35	9	3.27 (1.49-7.19)	.003
Thromboembolism	0.73 (0.32-1.67)	.46	117	4	0.45 (0.16-1.24)	.12
Major bleeding	2.52 (1.07-5.95)	.04	35	5	1.74 (0.58-5.24)	.33
Age 55-64 y (n = 3000)^b^						
Death	1.58 (1.29-1.93)	<.001	324	137	1.29 (1.02-1.63)	.04
Cardiovascular death	1.74 (1.32-2.29)	<.001	150	73	1.48 (1.06-2.05)	.02
Noncardiovascular death	1.32 (0.99-1.75)	.06	174	63	1.08 (0.78-1.51)	.64
Valve-related events						
Reoperation	2.62 (1.58-4.33)	<.001	35	32	2.77 (1.56-4.91)	<.001
Thromboembolism	0.66 (0.46-0.95)	.03	173	34	0.56 (0.37-0.85)	.006
Major bleeding	0.62 (0.35-1.10)	.10	74	14	0.54 (0.28-1.03)	.06
Age ≥65 y (n = 7189)^c^						
Death	1.14 (1.01-1.28)	.04	363	1834	0.77 (0.66-0.90)	.001
Cardiovascular death	0.92 (0.78-1.10)	.36	188	814	0.67 (0.53-0.84)	.001
Noncardiovascular death	1.25 (1.06-1.47)	.01	175	1020	0.95 (0.76-1.20)	.69
Valve-related events						
Reoperation	1.87 (0.93-3.74)	.08	5	103	3.11 (1.30-7.45)	.01
Thromboembolism	0.61 (0.49-0.75)	<.001	109	375	0.55 (0.41-0.73)	<.001
Major bleeding	0.29 (0.21-0.40)	<.001	38	90	0.39 (0.25-0.60)	<.001

Abbreviations: IPTW, inverse-probability-of-treatment weighting; aHR, adjusted hazard ratio.

^a^ IPTW analysis includes 1654 patients with mechanical prosthesis and 150 biological prosthesis.

^b^ IPTW analysis includes 2227 patients with mechanical prosthesis and 773 biological prosthesis.

^c^ IPTW analysis includes 944 patients with mechanical prosthesis and 6245 biological prosthesis.

In MVR, receiving a bioprosthesis was associated with a significantly higher long-term mortality than receiving a mechanical prosthesis in patients aged 55 to 69 years (aHR, 1.22; 95% CI, 1.04-1.44; *P* = .02) (Figure 2B). However, long-term mortality did not differ between the valve types in patients aged 40 to 54 years (aHR, 1.15; 95% CI, 0.65-2.03; *P* = .63) or in patients aged 70 to 79 years (aHR, 1.06; 95% CI, 0.79-1.42; *P* = .69) (Table 3 and Figure 2B; eFigure 4 in Supplement 1). In DVR, receiving a bioprosthesis was associated with higher mortality (aHR, 2.02; 95% CI, 1.28-3.19; *P* = .002) than receiving a mechanical prosthesis in patients aged 55 to 64 years (eTable 14 and eFigure 5 in Supplement 1), with no significant difference in the other age groups.

**Table 3.** Comparative Outcomes Associated With Mechanical vs Bioprosthetic Mitral Valve Replacement Using Competing-Risk Analysis

Outcome	Unadjusted		IPTW-adjusted			
	HR (95% CI)	*P* value	Events, No.		aHR (95% CI)	*P* value
			Mechanical prosthesis	Biological prosthesis		
Age <55 y (n = 2937)^a^						
Death	2.01 (1.43-3.06)	<.001	301	17	1.13 (0.65-1.98)	.67
Cardiovascular death	0.68 (0.30-1.54)	.36	170	3	0.34 (0.14-0.84)	.02
Noncardiovascular death	3.97 (2.52-6.26)	<.001	131	14	2.29 (1.17-4.46)	.02
Valve related events						
Reoperation	7.64 (4.77-12.23)	.000	16	1	4.58 (2.43-8.62)	<.001
Thromboembolism	0.58 (0.26-1.30)	.19	201	8	0.81 (0.26-2.56)	.72
Major bleeding	0.57 (0.18-1.82)	.35	101	3	0.63 (0.10-3.93)	.62
Age 55-69 y (n = 4231)^b^						
Death	1.77 (1.56-2.01)	<.001	692	334	1.22 (1.04-1.44)	.02
Cardiovascular death	1.78 (1.50-2.10)	<.001	385	194	1.25 (1.01-1.55)	.04
Noncardiovascular death	1.52 (1.25-1.85)	<.001	307	140	1.13 (0.88-1.45)	.33
Valve related events						
Reoperation	5.27 (3.68-7.56)	<.001	43	126	7.83 (5.19-11.82)	<.001
Thromboembolism	0.95 (0.76-1.19)	.67	266	107	0.99 (0.73-1.34)	.95
Major bleeding	1.20 (0.89-1.63)	.24	136	62	1.12 (0.75-1.68)	.57
Age ≥70 y (n = 1743)^c^						
Death	0.99 (0.78-1.24)	.90	71	662	1.09 (0.81-1.47)	.56
Cardiovascular death	0.94 (0.70-1.25)	.66	43	427	1.14 (0.77-1.68)	.51
Noncardiovascular death	0.99 (0.68-1.44)	.97	28	235	0.92 (0.58-1.49)	.75
Valve related events						
Reoperation	∞	<.001	0	54	∞	<.001
Thromboembolism	1.81 (0.99-3.33)	.06	9	157	2.28 (0.96-5.75)	.06
Major bleeding	1.04 (0.48-2.25)	.93	5	59	1.38 (0.53-3.61)	.51

Abbreviations: IPTW, inverse-probability-of-treatment weighting; aHR, adjusted hazard ratio.

^a^ IPTW analysis includes 2783 patients with mechanical prosthesis and 154 biological prosthesis.

^b^ IPTW analysis includes 2999 patients with mechanical prosthesis and 1232biological prosthesis.

^c^ IPTW analysis includes 175 patients with mechanical prosthesis and 1568 biological prosthesis.

Overall, the risk profiles of cardiovascular death according to prosthesis type were very similar to that of all-cause mortality (Table 2 and Table 3; eTable 14 in Supplement 1). However, the bioprosthetic MVR was associated with a lower risk of cardiovascular death (aHR, 0.34; 95% CI, 0.14-0.84; *P* = .02) in patients younger than 55 years that was not in all-cause death. The COD information is presented in eTable 15 to 17 in Supplement 1.

### Complications After Valve Replacement Surgery

#### Reoperation

The cumulative incidence of reoperation was significantly higher in the bioprosthesis group throughout all age strata, regardless of valve position (Table 2 and Table 3; eTable 14 in Supplement 1). The relative hazard of reoperation with bioprosthesis was more prominent in MVR and DVR (Table 3; eTable 14 in Supplement 1). In the patients aged 55 to 69 years who received MVR and patients aged 55 to 64 years who received DVR who received bioprosthetic valve replacement, the risk of reoperation was significantly higher (MVR: aHR, 7.75; 95% CI, 5.14-11.69; *P* < .001; DVR: aHR, 7.13; 95% CI, 3.26-13.18; *P* < .001), in agreement with significant differences in survival in this age group.

#### Systemic Thromboembolism and Major Bleeding

In AVR, the cumulative incidence of systemic thromboembolism was significantly higher with mechanical prosthesis in patients aged 55 years or older (eg, age ≥65 years: aHR, 0.55; 95% CI, 0.41-0.73; *P* < .001), but this was not the case in patients younger than 55 years (Table 2). However, the risk of major bleeding was greater with mechanical AVR only in patients older than 65 years (aHR, 0.39; 95% CI, 0.25-0.60; *P* < .001). In MVR and DVR, there were no differences in the risks of thromboembolism and major bleeding in any age strata (Table 3; eTable 14 in Supplement 1). The comparative outcomes without competing risk analysis are summarized in eTables 18 to 20 in Supplement 1.

### Subgroup Analyses According to the Risk Profiles

Subgroup risk analyses were conducted for the middle age groups (AVR: 55-64 years; MVR: 55-69 years) (eFigure 6 in Supplement 1). Survival benefits associated with the mechanical prosthesis were observed in most of the subgroups (eTable 21 and eTable 22 in Supplement 1), and there were no benefits associated with the use of bioprostheses in this age group in any subgroups, regardless of valve position. CCI (*P* for interaction = .04) and concomitant coronary bypass (*P* for interaction = .008) were identified as significant association modifiers in the comparison between mechanical vs bioprosthetic MVR in patients aged 55 to 69 years (eTable 22 in Supplement 1). There were no significant associations of mechanical MVR with survival in patients aged 55 to 69 years with high risk profiles, including CCI of 2 or greater (aHR, 1.05; 95% CI, 0.85-1.29; *P* = .68) or concomitant coronary bypass (aHR, 0.69; 95% CI, 0.45-1.07; *P* = .10).

### Sensitivity Analysis

In AVR and MVR, the comparative outcomes between the recipients of mechanical and bioprostheses were examined in the combined population of youngest and middle age strata (AVR: 40 to 64 years; MVR: 40 to 69 years). After IPTW adjustment, baseline profiles were relatively well-balanced for most covariates (eTable 23 and eTable 24 in Supplement 1). Overall, the results of the analysis conducted in the combined population were consistent with the individual findings of the youngest age strata and middle age strata (eTable 25 in Supplement 1). In MVR among patients aged 40 to 69 years, the risk of all-cause mortality was significantly higher for bioprosthesis than mechanical prosthesis (aHR, 1.36; 95% CI, 1.16-1.61; *P* < .001). However, there were no significant differences in the risks of cardiovascular death between the groups (aHR, 1.18; 95% CI, 0.95-1.47; *P* = .13), and the risk of noncardiovascular death was significantly higher for patients who received a bioprosthesis (aHR, 1.52; 95% CI, 1.19-1.94; *P* < .001).

## Discussion

In this nationwide cohort study conducted over 16 years, the long-term outcomes of mechanical prosthesis and bioprosthesis in aortic or mitral position were evaluated after age stratification. A survival benefit associated with mechanical prosthesis, compared with bioprosthesis, was observed in patients up to age 65 years in AVR and up to age 70 years in MVR. Mechanical AVR was associated with a lower risk of reoperation throughout all age strata but also greater risks of thromboembolism and bleeding in patients aged 55 years or older. Likewise, in the patients who underwent MVR or DVR, the risks of reoperation were also greater with bioprosthesis throughout all age strata, with no differences in the risks of thromboembolism and major bleeding in any age strata.

The findings of this study challenge the results of previous landmark studies,^2,3,16^ raising concerns regarding current clinical practice, in which the use of bioprosthesis is increasing in patients aged 70 years and younger.^17^ In this atmosphere of overwhelming preference of bioprosthesis, the survival benefit associated with mechanical prosthesis until age 65 years (for AVR) or age 70 years (for MVR) may encourage health care practitioners to adopt a more conservative approach while choosing a prosthetic valve. Previous studies comparing mechanical vs bioprosthetic AVR have shown discordant results.^3,16,18^ Statewide cohort studies in both New York and California showed no differences in mortality of patients aged 50 to 69 years and 55 to 64 years, respectively.^3,16^ However, in a similar nationwide analysis from Sweden by Glaser et al,^18^ patients aged 50 to 69 years receiving mechanical AVR had better long-term survival than those who received bioprosthetic AVR.^18^ Glaser et al attribute the difference between their results and the findings from the statewide study in New York to the high-quality anticoagulation management in Sweden. A similar explanation may be applied to our findings. In South Korea, the NHIS is mandatory for all legal residents and covers almost all major medical practices, except for cosmetic procedures or surgeries. Therefore, all expenses related to heart surgery and postoperative anticoagulation management are provided to patients without significant financial burden. In addition to this, the high population density and high accessibility of medical facilities in Korea may have contributed to high-quality anticoagulation management with very low rates of follow-up losses.

The ACC/AHA practice guidelines explain the importance of the shared decision-making process for prosthetic valve selection based on the values and preferences of informed patients.^5^ Informed patients may choose a prosthesis type while considering the trade-offs between the risks of reintervention and lifelong anticoagulation, based on their values. Although the decision-making process is centered around the patient’s choice, an important piece of information to offer patients is the comparative outcomes between mechanical and bioprosthesis in their age group. If the values, lifestyle, and preferences of the informed patient are important factors for the choice of prosthetic valve type, the information offered to patients based on clinical evidence may be the cornerstone of the decision. This information should be simple and balanced. Under the status quo, without compelling or indisputable evidence on this issue, we believe that our findings may be valuable in shared decision-making between patients and practitioners.

### Limitations and Strengths

This study has some limitations. To our knowledge, this study was the first to analyze patients who received DVR together, albeit not to a satisfactory level of statistical power. Due to the eccentric use of prosthesis in patients aged younger than 65 years in this setting, intergroup baseline profile imbalance and selection bias could not be effectively overcome. Particularly, in the age strata of patients younger than 55 years, the difference in case volume was too great at the intergroup baseline imbalance to be appropriately handled, so that the results of this population may have only limited meaning. Similarly, the prominent intergroup divergence of mortality in patients aged 55 to 64 years should be interpreted with caution. Nevertheless, the increased risk of reoperation in bioprosthetic DVR compared with mechanical prosthesis throughout all the age strata may have important implications.

Similarly, in AVR and MVR, the prosthetic valves were also used in an inconsistent pattern, with predominant use of mechanical prosthesis in the youngest population (<55 years) and predominant use of bioprosthesis in the oldest population (≥65 years in AVR; ≥70 years in MVR). Although the imbalances were addressed with a robust statistical effort, the eccentric use pattern between the groups is a limitation of this study.

This study was based on a claims administrative database for obtaining baseline profiles and outcomes. Thus, it may be subject to coding errors and omissions or misclassifications, along with lacking important variables, such as echocardiography data, cause of reoperation, and anticoagulation adequacy. Nonetheless, this study is strengthened by great accuracy and 100% completeness of survival information provided by Statistics Korea. Although cardiovascular death and other COD information were captured in this administrative data, the boundary between cardiovascular death and noncardiovascular death is arbitrary and unclear, as it may involve the subjective judgment of the recorder. Rather, hard end points, such as all-cause mortality, may be more valid and reliable in the analysis of administrative data.

In treating valvular heart disease, it has been recognized that there are potential interracial or interethnic differences in baseline profile, management, and outcomes.^19,20^ In particular, it has been continuously suggested that the target international normalized ratio after mechanical valve replacement in Asian populations may be different from that in the Western populations.^21,22,23^ Existing evidence regarding prosthetic valve type selection primarily use data from Western populations, without race-oriented evidence. Since this study was conducted among an East Asian population, the results may not be generalizable to other races, ethnicities, or countries but may have stronger race-specific implications for East Asian populations.

## Conclusions

In this nationwide cohort study comparing the long-term outcomes associated with mechanical and bioprostheses in AVR and MVR, mechanical prosthesis was associated with survival benefits in patients aged up to 65 years for AVR and in patients aged 55 to 69 years for MVR. The risk of reoperation was greater with bioprostheses, regardless of valve position in all age-strata, but it also had a protective association against thromboembolism or bleeding after AVR in patients aged 70 years or older. However, there were no significant differences in the risk of thromboembolism or bleeding after MVR and DVR in any age strata.

## Notes

Supplement 1. **eTable 1.** Definition of Baseline Comorbidities **eTable 2.** Definition of Operative Profiles **eTable 3.** Definition of Clinical Outcomes **eTable 4.** Trade Names of Mechanical or Bioprosthetic Valves Used for Aortic or Mitral Valve Replacement **eTable 5.** Baseline and Operative Characteristics of AVR Patients Aged 40 to 54 Years **eTable 6.** Baseline and Operative Characteristics of AVR Patients Aged 55 to 64 Years **eTable 7.** Baseline and Operative Characteristics of AVR Patients Aged 65 to 79 Years **eTable 8.** Baseline and Operative Characteristics of MVR Patients Aged 40 to 54 Years **eTable 9.** Baseline and Operative Characteristics of MVR Patients Aged 55 to 69 Years **eTable 10.** Baseline and Operative Characteristics of MVR Patients Aged 70 to 79 Years **eTable 11.** Baseline and Operative Characteristics of DVR Patients Aged 40 to 54 Years **eTable 12.** Baseline and Operative Characteristics of DVR Patients Aged 55 to 64 Years **eTable 13.** Baseline and Operative Characteristics of DVR Patients Aged 65 to 79 Years **eTable 14.** Comparative Outcomes of Mechanical vs Bioprosthetic DVR Using Competing-Risk Analysis **eTable 15.** Cause of Death Information After AVR **eTable 16.** Cause of Death Information After MVR **eTable 17.** Cause of Death Information After DVR **eTable 18.** Comparative Outcomes of Mechanical vs Bioprosthetic AVR Without Competing-Risk Analysis **eTable 19.** Comparative Outcomes of Mechanical vs Bioprosthetic MVR Without Competing-Risk Analysis **eTable 20.** Comparative Outcomes of Mechanical vs Bioprosthetic DVR Without Competing-Risk Analysis **eTable 21.** Adjusted Hazards of Bioprosthesis for Mortality According to Various Subgroups in AVR **eTable 22.** Adjusted Hazards of Bioprosthesis for Mortality According to Various Subgroups in MVR **eTable 23.** Baseline and Operative Characteristics of AVR Patients Aged 40 to 64 Years **eTable 24.** Baseline and Operative Characteristics of MVR Patients Aged 40 to 69 Years **eTable 25.** Comparative Outcomes of Mechanical vs Biologic Prosthesis in Patients Aged <65 Years in AVR, <70 Years in MVR, and 65 Years in DVR Using Competing-Risk Analysis **eFigure 1.** Patient Inclusion Flow Diagram **eFigure 2.** Distributions of Patients Undergoing Valve Replacements Depending on Age Strata **eFigure 3.** Distributions of Propensity Scores According to Age Strata in AVR, MVR, and DVR **eFigure 4.** Adjusted Risks of Mortality of Bioprosthesis in Patients Aged <55 Years in AVR and MVR **eFigure 5.** Adjusted Risks of Mortality of Bioprosthesis According to Age Strata in DVR **eFigure 6.** Adjusted Risks for Mortality According to Various Subgroups in the Middle Age Group

Supplement 2.
Data Sharing Statement

## References

[1] Schwarz F, Baumann P, Manthey J, . The effect of aortic valve replacement on survival. Circulation. 1982;66(5):1105-1110. doi:10.1161/01.CIR.66.5.1105 7127696

[2] Chikwe J, Chiang YP, Egorova NN, Itagaki S, Adams DH. Survival and outcomes following bioprosthetic vs mechanical mitral valve replacement in patients aged 50 to 69 years. JAMA. 2015;313(14):1435-1442. doi:10.1001/jama.2015.3164 25871669

[3] Goldstone AB, Chiu P, Baiocchi M, . Mechanical or biologic prostheses for aortic-valve and mitral-valve replacement. N Engl J Med. 2017;377(19):1847-1857. doi:10.1056/NEJMoa1613792 29117490 PMC9856242

[4] Russo A, Grigioni F, Avierinos JF, . Thromboembolic complications after surgical correction of mitral regurgitation incidence, predictors, and clinical implications. J Am Coll Cardiol. 2008;51(12):1203-1211. doi:10.1016/j.jacc.2007.10.058 18355659

[5] Otto CM, Nishimura RA, Bonow RO, ; Writing Committee Members. 2020 ACC/AHA guideline for the management of patients with valvular heart disease: a report of the American College of Cardiology/American Heart Association Joint Committee on Clinical Practice Guidelines. J Am Coll Cardiol. 2021;77(4):e25-e197. doi:10.1016/j.jacc.2020.11.018 33342586

[6] Vahanian A, Beyersdorf F, Praz F, ; ESC/EACTS Scientific Document Group. 2021 ESC/EACTS guidelines for the management of valvular heart disease. Eur Heart J. 2022;43(7):561-632. doi:10.1093/eurheartj/ehab395 34453165

[7] Hammermeister K, Sethi GK, Henderson WG, Grover FL, Oprian C, Rahimtoola SH. Outcomes 15 years after valve replacement with a mechanical versus a bioprosthetic valve: final report of the Veterans Affairs randomized trial. J Am Coll Cardiol. 2000;36(4):1152-1158. doi:10.1016/S0735-1097(00)00834-2 11028464

[8] Oxenham H, Bloomfield P, Wheatley DJ, . Twenty year comparison of a Bjork-Shiley mechanical heart valve with porcine bioprostheses. Heart. 2003;89(7):715-721. doi:10.1136/heart.89.7.715 12807838 PMC1767737

[9] Stassano P, Di Tommaso L, Monaco M, . Aortic valve replacement: a prospective randomized evaluation of mechanical versus biological valves in patients ages 55 to 70 years. J Am Coll Cardiol. 2009;54(20):1862-1868. doi:10.1016/j.jacc.2009.07.032 19892237

[10] Weber A, Noureddine H, Englberger L, . Ten-year comparison of pericardial tissue valves versus mechanical prostheses for aortic valve replacement in patients younger than 60 years of age. J Thorac Cardiovasc Surg. 2012;144(5):1075-1083. doi:10.1016/j.jtcvs.2012.01.024 22341653

[11] Cheol Seong S, Kim YY, Khang YH, . Data resource profile: the National Health Information Database of the National Health Insurance Service in South Korea. Int J Epidemiol. 2017;46(3):799-800. doi:10.1093/ije/dyw253 27794523 PMC5837262

[12] Choi EK. Cardiovascular research using the Korean National Health Information Database. Korean Circ J. 2020;50(9):754-772. doi:10.4070/kcj.2020.0171 32725984 PMC7441000

[13] Song SO, Jung CH, Song YD, . Background and data configuration process of a nationwide population-based study using the korean national health insurance system. Diabetes Metab J. 2014;38(5):395-403. doi:10.4093/dmj.2014.38.5.395 25349827 PMC4209354

[14] Park SJ, Jo AJ, Kim HJ, . Real-world outcomes of on- vs off-pump coronary bypass surgery: result from Korean Nationwide Cohort. Ann Thorac Surg. 2022;113(6):1989-1998. doi:10.1016/j.athoracsur.2021.07.035 34400133

[15] Austin PC, Fine JP. Propensity-score matching with competing risks in survival analysis. Stat Med. 2019;38(5):751-777. doi:10.1002/sim.8008 30347461 PMC6900780

[16] Chiang YP, Chikwe J, Moskowitz AJ, Itagaki S, Adams DH, Egorova NN. Survival and long-term outcomes following bioprosthetic vs mechanical aortic valve replacement in patients aged 50 to 69 years. JAMA. 2014;312(13):1323-1329. doi:10.1001/jama.2014.12679 25268439

[17] Alkhouli M, Alqahtani F, Simard T, Pislaru S, Schaff HV, Nishimura RA. Predictors of use and outcomes of mechanical valve replacement in the United States (2008-2017). J Am Heart Assoc. 2021;10(9):e019929. doi:10.1161/JAHA.120.019929 33870704 PMC8200758

[18] Glaser N, Jackson V, Holzmann MJ, Franco-Cereceda A, Sartipy U. Aortic valve replacement with mechanical vs. biological prostheses in patients aged 50-69 years. Eur Heart J. 2016;37(34):2658-2667. doi:10.1093/eurheartj/ehv580 26559386

[19] Kong WKF, Regeer MV, Poh KK, . Inter-ethnic differences in valve morphology, valvular dysfunction, and aortopathy between Asian and European patients with bicuspid aortic valve. Eur Heart J. 2018;39(15):1308-1313. doi:10.1093/eurheartj/ehx562 29029058

[20] Wilson JB, Jackson LR II, Ugowe FE, . Racial and ethnic differences in treatment and outcomes of severe aortic stenosis: a review. JACC Cardiovasc Interv. 2020;13(2):149-156. doi:10.1016/j.jcin.2019.08.056 31973792

[21] Huang JT, Chan YH, Wu VC, . Analysis of anticoagulation therapy and anticoagulation-related outcomes among asian patients after mechanical valve replacement. JAMA Netw Open. 2022;5(2):e2146026. doi:10.1001/jamanetworkopen.2021.46026 35103794 PMC8808330

[22] Limdi NA, Brown TM, Yan Q, . Race influences warfarin dose changes associated with genetic factors. Blood. 2015;126(4):539-545. doi:10.1182/blood-2015-02-627042 26024874 PMC4513254

[23] Shen AY, Yao JF, Brar SS, Jorgensen MB, Chen W. Racial/ethnic differences in the risk of intracranial hemorrhage among patients with atrial fibrillation. J Am Coll Cardiol. 2007;50(4):309-315. doi:10.1016/j.jacc.2007.01.098 17659197

