# Supplementary material for: Evaluating Reference Ages for Selecting Prosthesis Types for Heart Valve Replacement in Korea
Source: JAMA Netw Open. 2023 May 22;6(5):e2314671. doi: 10.1001/jamanetworkopen.2023.14671 (PMC10203891; doi:10.1001/jamanetworkopen.2023.14671)
Supplement: Supplement 1. — eTable 1. Definition of Baseline Comorbidities eTable 2. Definition of Operative Profiles eTable 3. Definition of Clinical Outcomes eTable 4. Trade Names of Mechanical or Bioprosthetic Valves Used for Aortic or Mitral Valve Replacement eTable 5. Baseline and Operative Characteristics of AVR Patients Aged 40 to 54 Years eTable 6. Baseline and Operative Characteristics of AVR Patients Aged 55 to 64 Years eTable 7. Baseline and Operative Characteristics of AVR Patients Aged 65 to 79 Years eTable 8. Baseline and Operative Characteristics of MVR Patients Aged 40 to 54 Years eTable 9. Baseline and Operative Characteristics of MVR Patients Aged 55 to 69 Years eTable 10. Baseline and Operative Characteristics of MVR Patients Aged 70 to 79 Years eTable 11. Baseline and Operative Characteristics of DVR Patients Aged 40 to 54 Years eTable 12. Baseline and Operative Characteristics of DVR Patients Aged 55 to 64 Years eTable 13. Baseline and Operative Characteristics of DVR Patients Aged 65 to 79 Years eTable 14. Comparative Outcomes of Mechanical vs Bioprosthetic DVR Using Competing-Risk Analysis eTable 15. Cause of Death Information After AVR eTable 16. Cause of Death Information After MVR eTable 17. Cause of Death Information After DVR eTable 18. Comparative Outcomes of Mechanical vs Bioprosthetic AVR Without Competing-Risk Analysis eTable 19. Comparative Outcomes of Mechanical vs Bioprosthetic MVR Without Competing-Risk Analysis eTable 20. Comparative Outcomes of Mechanical vs Bioprosthetic DVR Without Competing-Risk Analysis eTable 21. Adjusted Hazards of Bioprosthesis for Mortality According to Various Subgroups in AVR eTable 22. Adjusted Hazards of Bioprosthesis for Mortality According to Various Subgroups in MVR eTable 23. Baseline and Operative Characteristics of AVR Patients Aged 40 to 64 Years eTable 24. Baseline and Operative Characteristics of MVR Patients Aged 40 to 69 Years eTable 25. Comparative Outcomes of Mechanical vs Biologic Prosthesis in Patients Aged <65 Y [file jamanetwopen-e2314671-s001.pdf]

Supplemental Online Content

Park SJ, Ok YJ, Kim HJ, et al. Evaluating reference ages for selecting prosthesis types for heart valve replacement in Korea. *JAMA Netw Open*. 2023;6(5):e2314671. doi:10.1001/jamanetworkopen.2023.14671

eTable 1. Definition of Baseline Comorbidities
eTable 2. Definition of Operative Profiles
eTable 3. Definition of Clinical Outcomes
eTable 4. Trade Names of Mechanical or Bioprosthetic Valves Used for Aortic or Mitral Valve Replacement
eTable 5. Baseline and Operative Characteristics of AVR Patients Aged 40 to 54 Years
eTable 6. Baseline and Operative Characteristics of AVR Patients Aged 55 to 64 Years
eTable 7. Baseline and Operative Characteristics of AVR Patients Aged 65 to 79 Years
eTable 8. Baseline and Operative Characteristics of MVR Patients Aged 40 to 54 Years
eTable 9. Baseline and Operative Characteristics of MVR Patients Aged 55 to 69 Years
eTable 10. Baseline and Operative Characteristics of MVR Patients Aged 70 to 79 Years
eTable 11. Baseline and Operative Characteristics of DVR Patients Aged 40 to 54 Years
eTable 12. Baseline and Operative Characteristics of DVR Patients Aged 55 to 64 Years
eTable 13. Baseline and Operative Characteristics of DVR Patients Aged 65 to 79 Years
eTable 14. Comparative Outcomes of Mechanical vs Bioprosthetic DVR Using Competing-Risk Analysis
eTable 15. Cause of Death Information After AVR
eTable 16. Cause of Death Information After MVR
eTable 17. Cause of Death Information After DVR
eTable 18. Comparative Outcomes of Mechanical vs Bioprosthetic AVR Without Competing-Risk Analysis
eTable 19. Comparative Outcomes of Mechanical vs Bioprosthetic MVR Without Competing-Risk Analysis
eTable 20. Comparative Outcomes of Mechanical vs Bioprosthetic DVR Without Competing-Risk Analysis
eTable 21. Adjusted Hazards of Bioprosthesis for Mortality According to Various Subgroups in AVR
eTable 22. Adjusted Hazards of Bioprosthesis for Mortality According to Various Subgroups in MVR
eTable 23. Baseline and Operative Characteristics of AVR Patients Aged 40 to 64 Years
eTable 24. Baseline and Operative Characteristics of MVR Patients Aged 40 to 69 Years

© 2023 Park SJ et al. *JAMA Network Open.*

eTable 25. Comparative Outcomes of Mechanical vs Biologic Prosthesis in Patients Aged <65 Years in AVR, <70 Years in MVR, and 65 Years in DVR Using Competing-Risk Analysis
eFigure 1. Patient Inclusion Flow Diagram
eFigure 2. Distributions of Patients Undergoing Valve Replacements Depending on Age Strata
eFigure 3. Distributions of Propensity Scores According to Age Strata in AVR, MVR, and DVR
eFigure 4. Adjusted Risks of Mortality of Bioprosthesis in Patients Aged <55 Years in AVR and MVR
eFigure 5. Adjusted Risks of Mortality of Bioprosthesis According to Age Strata in DVR
eFigure 6. Adjusted Risks for Mortality According to Various Subgroups in the Middle Age Group

This supplemental material has been provided by the authors to give readers additional information about their work.

© 2023 Park SJ et al. *JAMA Network Open.*

eTable 1. Definition of baseline comorbidities

Comorbidities	ICD-10 codes	NHIS claim code	Number of diagnosis
Atrial fibrillation	I48	-	Admission or outpatient clinic ≥ 2
Hypertension	I10-I13, I15	-	Admission or outpatient clinic ≥ 3
Diabetes mellitus	E10-E14	-	Admission or outpatient clinic ≥ 3
Dyslipidemia	E78	-	Admission or outpatient clinic ≥ 3
Chronic kidney disease	N18	-	Admission or outpatient clinic ≥ 2
Dialysis	-	O701-O708	Admission or outpatient clinic ≥ 2
Ischemic stroke	I63, I64, I67.8-9	-	Admission or outpatient clinic ≥ 2
Transient ischemic attack	G45	-	Admission or outpatient clinic ≥ 2
Thromboembolism	I74	-	Admission or outpatient clinic ≥ 2
Ischemic heart disease	I20-I25	-	Admission or outpatient clinic ≥ 2
Myocardial infarction	I21-I23	-	Admission or outpatient clinic ≥ 2
Previous PCI	I20-I25	M6551-6554, M6561-M6567, M6571, M6572, M6634, M6638	Admission or outpatient clinic ≥ 1
Congestive heart failure	I50, I42, I11.0, I13.0, I13.2	-	Admission or outpatient clinic ≥ 2
Anemia	D50-D64	-	Admission or outpatient clinic ≥ 2
COPD	J44	-	Admission or outpatient clinic ≥ 2
Asthma	J45	-	Admission or outpatient clinic ≥ 2
Peripheral vascular disease	I70.1, I70.2, I70.8,	-	Admission or outpatient clinic ≥ 2

© 2023 Park SJ et al. *JAMA Network Open.*

	I70.9, I73		
Previous cardiac surgery	-	O1660, O1671, O1672, O1680, O1701-O1705, O1710, O1711, O1721-O1723, O1730, O1740, O1750, O1760, O1770, O1781-O1783, O1791-O1793, O1794-O1800, O1800, O1810, O1821-O1826, O1830, O1840, O1851, O1852, O1861, O1873-O1875, O1878, O1879, O0881-O0883, O1940, O1950, O1960, O1970, O1981, O1982, O2001, O2004, O2006, O2007, O1640, O1641, OA640, OA641, O1648, OA648, O1649, OA649, O1647, OA647, O2031-O2033	Admission ≥ 1
Previous cancer	C00-C97	-	Admission or outpatient clinic ≥ 1
Endocarditis	I33, I38, I39.0-1, I39.4, I39.8		Admission or outpatient clinic ≥ 1
Congestive heart failure	I50.0		Admission or outpatient clinic ≥ 1

PCI, percutaneous coronary intervention; COPD, chronic obstructive pulmonary disease; NOS, not otherwise specified

© 2023 Park SJ et al. *JAMA Network Open.*

eTable 2. Definition of operative profiles

Operative profiles	NHIS claim code
Procedure	
AVR	O1793, O1799
MVR	O1792
Prosthesis	
Mechanical valve	G2011025, G2011002, G2011007, G2011011, G2011012, G2011021, G2011129, G2011034, G2011029
Biological valve	G2001034, G2001102, G2001321, G2001002, G2001134, G2001203, G2001234, G2001003, G2001103, G2001121, G2001221, G2001021, G2001007, G2301002, G2301034
Concomitant procedure	
TV repair	O1781
CABG	O1640, O1641, O1647-O1649, OA640, OA641, OA647-OA649
Surgical ablation	O2006

AVR, aortic valve replacement; MVR, mitral valve replacement; MV, mitral valve; TV, tricuspid valve; CABG, coronary artery bypass grafting

© 2023 Park SJ et al. *JAMA Network Open.*

eTable 3. Definition of clinical outcomes

Comorbidities	ICD-10 codes	NHIS claim code	Additional diagnosis
Cardiovascular death	I00-I99		
Ischemic stroke	I63, I64	-	Hospitalization, brain imaging (CT or MRI)
Systemic thromboembolism	I74	-	Hospitalization, imaging (CT or MRI)
Major bleeding	I85.0, K22.1, K22.8, K25.0, K25.2, K25.4, K25.6, K26.0, K26.2, K26.4, K26.6, K27.0, K27.2, K27.4, K27.6, K28.0, K28.2, K28.4, K28.6, K29.0, K31.8, K55.2, K57.0, K57.1-K57.5, K57.8, K57.9, K62.5, K66.1, K92.0, K92.1, K92.2, D62, H05.2, H35.6, H43.1, J94.2, M25.0, R04	-	Hospitalization
Hemorrhagic stroke	I60-I62	-	Hospitalization, brain imaging (CT or MRI)

CT, computed tomography; MRI, magnetic resonance imaging.

© 2023 Park SJ et al. *JAMA Network Open.*

eTable 4. Trade names of mechanical or bioprosthetic valves used for aortic or mitral valve replacement.

Mechanical prosthesis	NHIS claim code	Biologic prosthesis	NHIS claim code
MECAHNICAL HEART VALVE (carbomedics, titanium)	G2011025	HANCOCK II VALVE	G2001003
EDWARDS MIRA MECHANICAL VALVE	G2011002	SOPRANO PERICARDIAL HEART VALVE	G2001034
SJM MASTERS SERIES VALVE	G2011007	CARPENTIER EDWARDS PERIMOUNT MAGNA TFX VALVE	G2001102
ON-X MECHANICAL HEART VALVE	G2011011	MOSAIC TISSUE VALVE	G2001103
BICARBON HEART VALVE (Sorin)	G2011012	SJM EPIC VALVE	G2001121
SJM REGENT MECHANICAL HEART VALVE	G2011021	TRIFECTA VALVE	G2001321
ATS HEART VALVE	G2011129	INTUITY ELITE VALVE SYSTEM	G2301002
BICARBON MECHANICAL HEART VALVE (Sorin)	G2011034	CARPENTIER EDWARDS PERIMOUNT VALVE	G2001002
ATS HEART VALVE	G2011029	PERICARBON MORE PERICARDIAL HEART VALVE	G2001134
		AVALUS BIOPROSTHESIS	G2001203
		EPIC SUPRA VALVE	G2001221
		MITROFLOW AORTIC PERICARDIAL HEART VALVE/ CROWN PRTAORTIC PERICARDIAL HEART VALVE	G2001234
		SJM BIOCOR PROCINE VALVE	G2001021
		TORONTO SPV VALVE	G2001007
		Perceval S	G2301034

© 2023 Park SJ et al. *JAMA Network Open.*

eTable 5. Baseline and operative characteristics of AVR (aortic valve replacement) patients aged 40 to 54 years

	Unadjusted				IPTW-adjusted		
	Mechanical prosthesis (n=1654)	Bioprosthetic prosthesis (n=150)	p-value	SMD	Mechanical prosthesis (n=1654)	Bioprosthetic prosthesis (n=150)	SMD
Baseline Demographics							
Age, years	48.6±4.1	49.3±4.3	0.033	0.177	48.6±4.1	48.4±4.3	0.055
Female	506 (30.6)	38 (25.3)	0.179	0.117	498.1 (30.1)	41.9 (27.9)	0.049
Baseline conditions							
Atrial fibrillation	51 (3.1)	2 (1.3)	0.313	0.119	48.7 (2.9(	3.6 (2.4)	0.035
Hypertension	641 (38.8)	62 (41.3)	0.535	0.053	640.6 (38.7)	49.2 (32,8)	0.124
Diabetes mellitus	178 (10.8)	20 (13.3)	0.335	0.079	181.4 (11.0)	18.5 (12.3)	0.042
Dyslipidemia	169 (10.2)	18 (12.0)	0.493	0.057	169.5 (10.2)	15.5 (10.3)	0.003
Chronic kidney disease	59 (3.6)	14 (9.3)	0.001	0.236	66.4 (4.0)	4.6 (3.1)	0.051
Dialysis	55 (3.3)	14 (9.3)	<0.001	0.249	63.3 (3.8)	7.7 (5.1)	0.062
Stroke, TIA or SE	87 (5.3)	7 (4.7)	0.754	0.027	88 (5.3)	13.9 (9.3)	0.153
Ischemic heart disease	384 (23.2)	25 (16.7)	0.067	0.164	375.4 (22.7)	30.5 (20.3)	0.058
Myocardial infarction	26 (1.6)	1 (0.7)	0.721	0.086	24.8 (1.5)	0.7 (0,5)	0.109
Previous PCI	25 (1.5)	3 (2.0)	0.502	0.037	24.9 (1.5)	1.1 (0.7)	0.076
Congestive heart failure	379 (22.9)	36 (24.0)	0.762	0.026	380.8 (23.0)	33.4 (22.3)	0.018
Anemia	164 (9.9)	30 (20.0)	<0.001	0.286	175 (10.6)	17 (11.3)	0.024
COPD	28 (1.7)	1 (0.7)	0.507	0.095	26.2 (1.6)	0.2 (0.1)	0.158
Asthma	114 (6.9)	12 (8.0)	0.610	0.042	115.7 (7.0)	10.2 (6.8)	0.006
Peripheral vascular disease	62 (3.7)	2 (1.3)	0.126	0.154	58.7 (3.5)	2.3 (1.5)	0.13
Previous cardiac surgery	3 (0.2)	0 (0.0)	1.000	0.06	2.8 (0.2)	0 (0.0)	0.058
Previous cancer	42 (2.5)	12 (8.0)	0.001	0.246	49.2 (3.0)	6 (4.0)	0.055
Charlson comorbidity index			0.020	0.252			0.001
0	683 (41.3)	55 (36.7)	0.004	0.286	675.4 (40.8)	50.7 (33.8)	0.192

© 2023 Park SJ et al. *JAMA Network Open.*

1	467 (28.2)	34 (22.7)			462.1 (27.9)	53.3 (35.5)	
2	253 (15.3)	25 (16.7)			255 (15.4)	25.4 (16.9)	
≥ 3	169 (10.2)	18 (12.0)			170.2 (10.3)	12.7 (8.5)	
≥ 5	82 (5.0)	18 (12.0)			91.4 (5.5)	7.8 (5.2)	
Years of Surgery			<0.001	0.472			0.166
2002~2005	398 (24.1)	18 (12.0)			381.7 (23.1)	28.5 (19.0)	
2006~2009	415 (25.1)	25 (16.7)			402.5 (24.3)	31.2 (20.8)	
2010~2013	405 (24.5)	40 (26.7)			407.2 (24.6)	38.7 (25.8)	
2014~2018	436 (26.4)	67 (44.7)			462.7 (28.0)	51.7 (34.5)	
Level of hospital			0.033	0.174			0.197
*Tertiary general hospital	1304 (78.8)	107 (71.3)			1304.5 (78.9)	105.5 (70.3)	
General hospital	350 (21.2)	43 (28.7)			349.5 (21.1)	44.5 (29.7)	
Cumulative hospital volume for cardiac surgery							
<250 cases	424 (25.6)	40 (26.7)	0.032	0.253	426.9 (25.8)	41.6 (27.7)	0.15
250-999 cases	423 (25.6)	53 (35.3)			434.9 (26.3)	47.1 (31.4)	
1000-2999 cases	498 (30.1)	38 (25.3)			492 (29.7)	39.1 (26.1)	
≥3000 cases	309 (18.7)	19 (12.7)			300.2 (18.1)	22.1 (14.7)	
Endocarditis	325 (19.6)	46 (30.7)	0.001	0.256	339.3 (20.5)	35.1 (23.4)	0.07
Congestive heart failure	423 (25.6)	43 (28.7)	0.407	0.07	428.2 (25.9)	42.6 (28.4)	0.056
Bicuspid aortic valve	239 (14.4)	30 (20.0)	0.068	0.147	247.5 (15.0)	25.8 (17.2)	0.061
Mode of valve disease			0.365	0.149			0.232
Aortic stenosis	338 (20.4)	24 (16.0)			335.3 (20.3)	18.6 (12.4)	
Aortic regurgitation	701 (42.4)	69 (46.0)			703.2 (42.5)	72.7 (48.5)	
Combined	558 (33.7)	49 (32.7)			555.8 (33.6)	50.6 (33.7)	
Unspecified	57 (3.4)	8 (5.3)			59.7 (3.6)	8.1 (5.4)	
Concomitant Procedure							
Tricuspid valve repair	44 (2.7)	3 (2.0)	0.793	0.044	43(2.6)	2 (1.3)	0.09
Coronary arterial bypass grating	88 (5.3)	6 (4.0)	0.486	0.063	85.9 (5.2)	7.9 (5.3)	0.004

© 2023 Park SJ et al. *JAMA Network Open.*

Surgical ablation for atrial fibrillation	49 (3.0)	4 (2.7)	1.000	0.018	49 (3.0)	6.1 (4.1)	0.06
Health Screening Data							
Height, m	165.3±8.7	165.8±7.1	0.586	0.055	165.4±8.7	165.1±6.9	0.039
Weight, kg	66.5±11.9	65.7±12.4	0.550	0.065	66.4±11.9	65.1±11.2	0.11
BMI, kg/m^2	24.2±3.3	23.8±3.5	0.247	0.124	24.2±3.3	23.8±3.0	0.122
< 18.5	19 (1.1)	6 (4.0)	0.071	0.22	24 (1.5)	3.1 (2.1)	0.126
≥ 18.5 and < 23	354 (24.1)	35 (23.3)			357.1 (21.6)	35.3 (23.5)	
≥ 23 and < 25	244 (14.8)	18 (12.0)			240.6 (14.5)	24.1 (16.1)	
≥ 25 and < 30	339 (20.5)	25 (16.7)			332.7 (20.1)	23.9 (15.9)	
≥ 30	50 (3.0)	5 (3.3)			50.2 (3.0)	3.9 (2.6)	
Not available	648 (39.2)	61 (40.7)			649.3 (39.3)	59.9 (39.9)	
Systolic blood pressure, mmHg	124.9±17.6	123.6 (14.6)	0.423	0.082	124.8 (17.6)	125.5±15.4	0.041
< 120	369 (22.3)	34 (22.7)	0.932	0.058	369.5 (22.3)	32.5 (21.7)	0.056
≥ 120 and < 140	443 (26.8)	40 (26.7)			443.1 (26.8)	37.9 (25,3)	
≥ 140	194 (11.7)	15 (10.0)			192.1 (11.6)	19.7 (13.1)	
Not available	648 (39.2)	61 (40.7)			649.3 (39.3)	59.9 (39.9)	
Diastolic blood pressure, mmHg	75.6±11.6	74.2±11.2	0.264	0.125	75.5±11.7	74.7±11.4	0.063
< 80	554 (33.5)	51 (34)	0.901	0.067	555.9 (33.6)	49.1 (32.7)	0.051
≥ 80 and < 90	326 (19.7)	29 (19.2)			325.2 (19.7)	31.4 (20.9)	
≥ 90	126 (7.6)	9 (6.0)			123.6 (7.5)	9.7 (6.5)	
Not available	648 (39.2)	61 (40.7)			649.3 (39.3)	59.9 (39.9)	
Smoking			0.862	0.074			0.116
Never smoker	484 (29.3)	40 (26.7)			480.6(29.1)	37.3 (24.9)	
Previous smoker	226 (13.7)	19 (12.7)			226 (13.7)	25.3 (16.9)	
Current smoker	284 (17.2)	28 (18.7)			285.5 (17.3)	26.7 (17.8)	

© 2023 Park SJ et al. *JAMA Network Open.*

Not available	660 (39.9)	63 (42.0)			661.8 (40.0)	60.7 (40.5)	
Alcohol use			0.853	0.076			0.037
None	322 (19.5)	30 (20.0)			323.6 (19.6)	30.2 (20.1)	
Mild-to-moderate	619 (37.4)	51 (34.0)			614.7 (37.2)	54.8 (36.5)	
Heavy	56 (3.4)	6 (4.0)			56.6 (3.4)	4.3 (2.9)	
Not available	657 (39.7)	63 (42.0)			659.1 (39.8)	60.7 (40.5)	
Creatinine, mg/dL			0.005	0.225			0.174
<= 1.5	630 (38.1)	6 (43.3)5			639.9 (38.7)	61 (40.7)	
> 1.5	19 (1.1)	6 (4.0)			20.4 (1.2)	5.6 (3.7)	
Not available	1005 (60.8)	79 (52.7)			993.6 (60.1)	83.3 (55.5)	
eGFR, mL/min/1.73m^2			0.021	0.201			0.111
≥ 60	503 (30.4)	52 (34.7)			514.8 (31.1)	49.6 (33.1)	
< 60	29 (1.8)	7 (4.7)			30.9 (1.9)	5.2 (3.5)	
Not available	1122 (67.8)	91 (60.7)			1108.2 (67.0)	95.3 (63.5)	

*Designated and certificated by the Ministry of Health and Welfare

Values are n (%), or mean ± standard deviation, unless otherwise indicated.

IPTW, inverse-probability-of-treatment weighting; SMD, standardized mean difference; BMI, body mass index; TIA, transient ischemic attack; SE, systemic embolization; PCI, percutaneous coronary intervention; COPD, chronic obstructive pulmonary disease; eGFR, estimated glomerular filtration rate; AVR, aortic valve replacement; MVR, mitral valve replacement.

© 2023 Park SJ et al. *JAMA Network Open.*

eTable 6. Baseline and operative characteristics of AVR (aortic valve replacement) patients aged 55 to 64 years

	**Unadjusted**				**IPTW-adjusted**		
	Mechanical prosthesis (n = 2227)	Biological prosthesis (n = 773)	p-value	SMD	Mechanical prosthesis (n = 2227)	Biological prosthesis (n = 773)	SMD
Baseline Demographics							
Age, years	59.5±2.8	61.3±2.5	<0.001	0.675	60.0±2.8	60.1±2.8	0.046
Female	790 (35.5)	289 (37.4)	0.34	0.04	801.1 (36.0)	289.2 (37.4)	0.03
Baseline Comorbidities							
Atrial fibrillation	128 (5.7)	31 (4.0)	0.063	0.081	118.2 (5.3)	41 (5.3)	<0.001
Hypertension	1277 (57.3)	478 (61.8)	0.029	0.092	1293.8 (58.1)	439.7 (56.9)	0.024
Diabetes mellitus	500 (22.5)	192 (24.8)	0.175	0.056	512.7 (23.0)	177.4 (22.9)	0.002
Dyslipidemia	406 (18.2)	172 (22.3)	0.015	0.1	438.4 (19.7)	160.6 (20.8)	0.027
Chronic kidney disease	97 (4.4)	61 (7.9)	<0.001	0.148	113.9 (5.1)	42.3 (5.5)	0.016
Dialysis	69 (3.1)	49 (6.3)	<0.001	0.153	85.3 (3.8)	34.5 (4.5)	0.032
Stroke, TIA or SE	173 (7.8)	55 (7.1)	0.555	0.025	170.4 (7.7)	57.9 (7.5)	0.006
Ischemic heart disease	813 (36.5)	282 (36.5)	0.99	0.001	810.4 (36.4)	273 (35.3)	0.022
Myocardial infarction	70 (3.1)	28 (3.6)	0.519	0.026	72.5 (3.3)	19.9 (2.6)	0.041
Previous PCI	91 (4.1)	38 (4.9)	0.327	0.04	99.2 (4.5)	30.2 (3.9)	0.027
Congestive heart failure	536 (24.1)	213 (27.6)	0.054	0.08	563.1 (25.3)	202.7 (2.62)	0.022
Anemia	176 (7.9)	93 (12.0)	0.001	0.138	199.7 (9.0)	71.9 (9.3)	0.012
COPD	76 (3.4)	34 (4.4)	0.209	0.051	83.6 (3.8)	30.1 (3.9)	0.007
Asthma	251 (11.3)	95 (12.3)	0.445	0.032	260 (11.7)	97.3 (12.6)	0.028
Peripheral vascular disease	115 (5.2)	56 (7.2)	0.032	0.086	124.8 (5.6)	38.5 (5.0)	0.028
Previous cardiac surgery	12 (0.5)	1 (0.1)	0.204517	0.071	9.2 (0.4)	0.4 (0.1)	0.076
Previous cancer	112 (5.0)	70 (9.1)	<0.001	0.158	134 (6.0)	49.4 (6.4)	0.016

© 2023 Park SJ et al. *JAMA Network Open.*

Charlson comorbidity index			<0.001	0.173			0.028
0	682 (30.6)	181 (23.4)	<0.001	0.19	640.3 (28.8)	215.1 (27.8)	0.039
1	580 (26.0)	192 (24.8)			576.7 (25.9)	201.4 (26.1)	
2	390 (17.5)	153 (19.8)			393.7 (17.7)	130.5 (16.9)	
≥ 3	378 (17.0)	162 (21.0)			406.1 (18.2)	148.9 (19.3)	
≥ 5	197 (8.8)	85 (11.0)			210.3 (9.4)	77.1 (10.0)	
Years of Surgery			<0.001	0.352			0.08
2002~2005	430 (19.3)	95 (12.3)			386.7 (17.4)	117.6 (15.2)	
2006~2009	496 (22.3)	149 (19.3)			481.6 (21.6)	177.1 (22.9)	
2010~2013	594 (26.7)	157 (20.3)			553.9 (24.9)	179.5 (23.2)	
2014~2018	707 (31.7)	372 (48.1)			804.8 (36.1)	298.8 (38.7)	
Level of hospital			<0.001	0.173			0.018
*Tertiary general hospital	1821 (81.8)	577 (74.6)			1779.4 (79.9)	623.2 (80.6)	
General hospital	406 (18.2)	196 (25.4)			447.6 (20.1)	149.8 (19.4)	
Cumulative hospital volume for cardiac surgery			0.002	0.163			0.093
<250	551 (24.7)	188 (24.3)			559.9 (25.1)	203.7 (26.4)	
250-999	611 (27.4)	248 (32.1)			646.3 (29.0)	248.2 (32.1)	
1000-2999	610 (27.4)	223 (28.8)			602.8 (27.1)	196.3 (25.4)	
≥3000	455 (20.4)	114 (14.7)			417.9 (18.8)	124.8 (16.1)	
Endocarditis	275 (12.3)	113 (14.6)	0.105	0.066	285.3 (12.8)	100.4 (13.0)	0.005
Congestive heart failure	537 (24.1)	205 (26.5)	0.181	0.055	557.9 (25.1)	200.1 (25.9)	0.019
Bicuspid aortic valve	307 (13.8)	100 (12.9)	0.553	0.025	300.8 (13.5)	109.7 (14.2)	0.02
Mode of valve disease			0.012	0.137			0.114
Aortic stenosis	745 (33.5)	306 (39.6)			781.2 (35.1)	309.7 (40.1)	
Aortic regurgitation	547 (24.6)	167 (21.6)			532.7 (23.9)	165.8 (21.4)	
Combined	872 (39.2)	274 (35.4)			851.6 (38.2)	271.8 (35.2)	
Unspecified	63 (2.8)	26 (3.4)			61.5 (2.8)	25.7 (3.3)	

© 2023 Park SJ et al. *JAMA Network Open.*

Concomitant Procedure							
Tricuspid valve repair	71 (3.2)	12 (1.6)	0.017	0.108	60.8 (2.7)	16.5 (2.1)	0.039
Coronary arterial bypass grating	229 (10.3)	93 (12.0)	0.176	0.056	236.3 (10.6)	77.6 (10.0)	0.019
Surgical ablation for atrial fibrillation	103 (4.6)	25 (3.2)	0.099	0.072	96.2 (4.3)	37.9 (4.9)	0.027
Health Screening Data							
Height, m	162.4±8.3	161.5±8.4	0.029	0.108	162.3 (8.26)	161.61 (8.41)	0.083
Weight, kg	65.0±10.8	63.9±10.6	0.042	0.101	64.7±10.7	64.1±11.0	0.06
BMI, kg/m^2	24.6±3.2	24.5±3.4	0.494	0.033	24.5±3.2	24.5±3.6	0.004
< 18.5	19 (0.9)	16 (2.1)	0.074	0.123	31.6 (1.4)	10.9 (1.4)	0.065
≥ 18.5 and < 23	481 (21.6)	161 (20.8)			479.1 (21.5)	183.5 (23.7)	
≥ 23 and < 25	403 (18.1)	150 (19.4)			413.2 (18.6)	136.5 (17.7)	
≥ 25 and < 30	572 (25.7)	208 (26.9)			570.6 (25.6)	192.6 (34.9)	
≥ 30	87 (3.9)	25 (3.2)			84.5 (3.8)	34 (4.4)	
Not available	665 (29.9)	213 (27.6)			647.9 (29.1)	215.5 (27.9)	
Systolic blood pressure, mmHg	126.2±16.2	127.6±17.1	0.09	0.082	126.5±16.3	126.6±16.7	0.006
< 120	480 (21.6)	172 (22.3)	0.249	0.084	483.8 (21.7)	170.6 (22.1)	0.029
≥ 120 and < 140	775 (34.8)	261 (33.8)			774.4 (34.8)	276.5 (35.8)	
≥ 140	307 (13.8)	127 (16.4)			320.8 (14.4)	110.4 (14.3)	
Not available	665 (29.9)	213 (27.6)			647.9 (29.1)	215.5 (27.9)	
Diastolic blood pressure, mmHg	75.9±10.7	75.49 (10.9)	0.484	0.034	75.7±10.7	75.9±10.5	0.016
< 80	849 (38.1)	312 (40.4)	0.618	0.056	864.6 (38.8)	303.5 (39.3)	0.032
≥ 80 and < 90	531 (23.8)	185 (23.9)			537.6 (24.1)	194.2 (25.1)	
≥ 90	182 (8.2)	63 (8.2)			176.9 (7.9)	59.8 (7.7)	
Not available	665 (29.9)	213 (27.6)			647.9 (29.1)	215.5 (27.9)	

© 2023 Park SJ et al. *JAMA Network Open.*

Smoking			0.666	0.053			0.038
Never smoker	874 (39.2)	316 (40.9)			880.6 (39.5)	318.5 (41.2)	
Previous smoker	341 (15.3)	118 (15.3)			338.2 (15.2)	113.4 (14.7)	
Current smoker	336 (15.1)	122 (15.8)			349.2 (15.7)	122.2 (15.8)	
Not available	676 (30.4)	217 (28.1)			659 (29.6)	218.9 (28.3)	
Alcohol use			0.007	0.145			0.036
None	703 (31.6)	290 (37.5)			734.8 (33.0)	266.8 (34.5)	
Mild-to-moderate	753 (33.8)	226 (29.2)			728.9 (32.7)	251.9 (32.6)	
Heavy	98 (4.4)	42 (5.4)			108.2 (4.9)	37.3 (4.8)	
Not available	673 (30.2)	215 (27.8)			655 (29.4)	217 (28.1)	
Creatinine, mg/dL			0.001	0.162			0.05
<= 1.5	1072 (48.1)	432 (55.69)			1114.1 (50.0)	404.5 (52.3)	
> 1.5	48 (2.2)	19 (2.5)			50.3 (2.3)	14.6 (1.9)	
Not available	1107 (49.7)	322 (41.7)			1062.6 (47.7)	353.9 (45.8)	
eGFR, mL/min/1.73m^2			<0.001	0.207			0.058
≥ 60	784 (35.2)	337 (43.6)			834.9 (37.5)	311.2 (40.3)	
< 60	107 (4.8)	51 (6.6)			118.9 (5.3)	41.5 (5.4)	
Not available	1336 (60.0)	385 (49.8)			1273.2 (57.2)	420.3 (54.4)	

*Designated and certificated by the Ministry of Health and Welfare

Values are n (%), or mean ± standard deviation, unless otherwise indicated.
IPTW, inverse-probability-of-treatment weighting; SMD, standardized mean difference; BMI, body mass index; TIA, transient ischemic attack; SE, systemic embolization; PCI, percutaneous coronary intervention; COPD, chronic obstructive pulmonary disease; eGFR, estimated glomerular filtration rate; AVR, aortic valve replacement; MVR, mitral valve replacement.

© 2023 Park SJ et al. *JAMA Network Open.*

eTable 7. Baseline and operative characteristics of AVR (aortic valve replacement) patients aged 65 to 79 years

	**Unadjusted**				**IPTW-adjusted**		
	Mechanical prosthesis (n = 944)	Biological prosthesis (n = 6245)	p-value	SMD	Mechanical prosthesis (n = 944)	Biological prosthesis (n = 6245)	SMD
Baseline Demographics							
Age, years	68.5±3.3	72.3±4.0	<0.001	1.063	71.2±4.0	71.8±4.1	0.162
Female	395 (41.8)	2873 (46.0)	0.017	0.084	421 (44.6)	2839 (45.5)	0.017
Baseline conditions							
Atrial fibrillation	98 (10.4)	423 (6.8)	<0.001	0.129	85 (9.0)	451 (7.2)	0.065
Hypertension	645 (68.3)	4614 (73.9)	<0.001	0.123	692 (73.3)	4570 (73.2)	0.004
Diabetes mellitus	255 (27.0)	2055 (32.9)	<0.001	0.129	293 (31.0)	2005 (32.1)	0.024
Dyslipidemia	193 (20.4)	1672 (26.8)	<0.001	0.149	259 (27.4)	1614 (25.8)	0.036
Chronic kidney disease	44(4.7)	331 (5.3)	0.41	0.029	53 (5.6)	325 (5.2)	0.016
Dialysis	28 (3.0)	172 (2.8)	0.712	0.013	40 (4.2)	174 (2.8)	0.077
Stroke, TIA or SE	113 (12.0)	892 (14.3)	0.056	0.069	131 (13.9)	871 (13.9)	0.003
Ischemic heart disease	414 (43.9)	2926 (46.9)	0.085	0.06	452 (47.8)	2896 (46.6)	0.029
Myocardial infarction	34 (3.6)	261 (4.2)	0.404	0.03	57 (6.0)	259 (4.2)	0.085
Previous PCI	52 (5.5)	509 (8.2)	0.005	0.105	78 (8.3)	488 (7.8)	0.018
Congestive heart failure	298 (31.6)	2036 (32.6)	0.527	0.022	321 (34.0)	2033 (32.6)	0.029
Anemia	98 (10.4)	786 (12.6)	0.055	0.069	132 (14.0)	772 (12.4)	0.048
COPD	56 (5.9)	485 (7.8)	0.046	0.073	67 (7.1)	470 (7.5)	0.017
Asthma	153 (16.2)	1146 (18.4)	0.111	0.057	187 (19.8)	1131 (18.1)	0.043
Peripheral vascular disease	79 (8.4)	571 (9.1)	0.439	0.027	95 (10.1)	567 (9.1)	0.034
Previous cardiac surgery	4 (0.4)	42 (0.7)	0.372	0.034	3 (0.3)	40 (0.6)	0.045
Previous cancer	73 (7.7)	601 (9.6)	0.063	0.067	83 (8.8)	586 (9.4)	0.021

© 2023 Park SJ et al. *JAMA Network Open.*

Charlson comorbidity index			<0.001	0.159			0.036
0	192 (20.3)	996 (15.9)	<0.001	0.208	143 (15.1)	1034 (16.6)	0.05
1	246 (26.1)	1323 (21.2)			206 (21.8)	1358 (21.7)	
2	194 (20.6)	1301 (20.8)			205 (21.8)	1302 (20.8)	
≥ 3	181 (19.2)	1581 (25.3)			226 (23.9)	1529 (24.5)	
≥ 5	131 (13.9)	1044 (16.7)			165 (17.4)	1022 (16.4)	
Years of Surgery			<0.001	0.381			0.111
2002~2005	196 (20.8)	588 (9.4)			125 (13.2)	684 (11.0)	
2006~2009	214 (22.7)	1195 (19.1)			209 (22.1)	1223 (19.6)	
2010~2013	256 (27.1)	1804 (28.9)			264 (28.0)	1786 (28.6)	
2014~2018	278 (29.4)	2658 (42.6)			346 (36.6)	2552 (40.9)	
Level of hospital			0.049	0.381			0.051
*Tertiary general hospital	730 (77.3)	4643 (74.3)			679.4 (72.0)	4636 (74.2)	
General hospital	214 (22.7)	1602 (25.7)			265 (28.0)	1609 (25.8)	
Cumulative hospital volume for cardiac surgery			<0.001	0.338			0.196
<250 cases	280 (29.7)	1695 (27.1)			310.8 (32.9)	1724.7(27.6)	
250-999 cases	279 (29.6)	1708 (27.3)			296.8 (31.4)	1731.7 (27.7)	
1000-2999 cases	167 (17.7)	1920 (30.7)			202.4 (21.4)	1811.7 (29.0)	
≥3000 cases	218 (23.1)	922 (14.8)			134 (14.2)	976.9 (15.6)	
Endocarditis	97 (10.3)	533 (8.5)	0.078	0.06	91.9 (1.5)	549 (8.8)	0.033
Congestive heart failure	282 (29.9)	1769 (28.3)	0.327	0.034	296.1 (4.7)	1795 (28. 7)	0.057
Bicuspid aortic valve	76 (8.1)	387 (6.2)	0.031	0.072	58.4 (6.2)	404 (6.5)	0.012
Mode of valve disease			<0.001	0.197			0.084
Aortic stenosis	410 (43.4)	3319 (53.1)			460.8 (48.8)	3241.9 (51.9)	
Aortic regurgitation	151 (16.0)	794 (12.7)			121.4 (12.9)	823.6 (13.2)	
Combined	362 (38.3)	2002 (32.1)			334.2 (35.4)	2051.8 (32.9)	
Unspecified	21 (2.2)	130 (2.1)			27.6 (2.9)	127.7 (2.0)	

© 2023 Park SJ et al. *JAMA Network Open.*

Concomitant Procedure							
Tricuspid valve repair	49 (5.2)	166 (2.7)	<0.001	0.131	35.6 (3.8)	189.8 (3.0)	0.041
Coronary arterial bypass grating	133 (14.1)	1176 (18.8)	<0.001	0.128	160.7 (17.0)	1136.9 (18.2)	0.031
Surgical ablation for atrial fibrillation	55 (5.8)	329 (5.3)	0.477	0.024	49.2 (5.2)	334.4 (5.4)	0.007
Health Screening Data							
Height, m	160.1±3.4	158.8±8.6	<0.001	0.161	159.2±8.7	158.9 (8.62)	0.031
Weight, kg	62.7±10.0	61.4±10.0	0.001	0.135	61.5±10.2	61.5±10.0	0.006
BMI, kg/m^2	24.4±3.0	24.3±3.3	0.488	0.028	24.2±3.0	24.3±3.2	0.047
< 18.5	11 (1.2)	116 (1.9)			16.9 (1.8)	110.2 (1.8)	0.097
≥ 18.5 and < 23	211 (22.4)	1440 (23.1)			240.4 (25.5)	1444.2 (23.1)	
≥ 23 and < 25	171 (18.1)	1098 (17.6)			146.3 (15.5)	1102.3 (17.7)	
≥ 25 and < 30	257 (27.2)	1607 (25.7)			235.4 (24.9)	1616 (25.9)	
≥ 30	27 (2.9)	231 (3.7)			24.7 (2.6)	222.4 (3.6)	
Not available	267 (28.3)	1753 (28.1)			280.4 (29.7)	1750 (28.0)	
Systolic blood pressure, mmHg	129.5±17.3	130.3±17.3	0.227	0.05	129.8±17.3	130.2±17.3	0.022
< 120	175 (18.5)	1074 (17.2)	0.772	0.037	157.6(16.7)	1084.1 (17.4)	0.037
≥ 120 and < 140	318 (33.7)	2164 (34.7)			326.7 (34.6)	2157.2 (34.5)	
≥ 140	184 (19.5)	1239 (19.8)			179.3 (19.0)	1240.1 (19.9)	
Not available	267 (28.3)	1768 (28.3)			280.4 (29.7)	1763.6 (28.2)	
Diastolic blood pressure, mmHg	76.2±11.3	75.8±11.1	0.4	0.035	75.5±11.5	75.9±11.2	0.037
< 80	359 (38.0)	2537 (40.6)	0.353	0.063	378.7 (40.1)	2516.4 (40.3)	0.038
≥ 80 and < 90	217 (23.0)	1340 (21.5)			199.2 (21.1)	1351.6 (21.6)	
≥ 90	101 (10.7)	600 (9.6)			85.7 (9.1)	613.3 (9.8)	
Not available	267 (28.3)	1768 (28.3)			280.4 (29.7)	1763.6 (28.2)	

© 2023 Park SJ et al. *JAMA Network Open.*

Smoking			0.091	0.088			0.046
Never smoker	413 (43.8)	2988 (47.8)			429.2 (45.5)	2958.5 (47.4)	
Previous smoker	150 (15.9)	863 (13.8)			138.7 (14.7)	884 (14.2)	
Current smoker	92 (9.7)	554 (8.9)			80.8 (8.6)	558 (8.9)	
Not available	289 (30.6)	1840 (29.5)			295.2 (31.3)	1844.5 (29.5)	
Alcohol use			<0.001	0.222			0.114
None	333 (35.3)	2752 (44.1)			358.2 (37.9)	2677.6 (42.9)	
Mild-to-moderate	290 (30.7)	1387 (22.2)			256.8 (27.2)	1465.1 (23.5)	
Heavy	33 (3.5)	266 (4.3)			35 (3.7)	259.1 (4.1)	
Not available	288 (30.5)	1840 (29.5)			294 (31.1)	1843.2 (29.5)	
Creatinine, mg/dL			<0.001	0.187			0.123
<= 1.5	455 (48.2)	3457 (55.4)			456 (48.3)	3393.9 (54.3)	
> 1.5	17 (1.8)	204 (3.3)			29.9 (3.2)	198.7 (3.2)	
Not available	472 (50.0)	2584 (41.4)			458.1 (48.5)	2652.4 (42.5)	
eGFR, mL/min/1.73m^2			<0.001	0.18			0.095
≥ 60	299 (31.7)	2206 (35.3)			294.9 (31.2)	2168.4 (34.7)	
< 60	75 (7.9)	756 (12.1)			98.2 (10.4)	725.4 (11.6)	
Not available	570 (60.4)	3283 (52.6)			550.9 (58.4)	3351.2 (53.7)	

*Designated and certificated by the Ministry of Health and Welfare

Values are n (%), or mean ± standard deviation, unless otherwise indicated.
IPTW, inverse-probability-of-treatment weighting; SMD, standardized mean difference; BMI, body mass index; TIA, transient ischemic attack; SE, systemic embolization; PCI, percutaneous coronary intervention; COPD, chronic obstructive pulmonary disease; eGFR, estimated glomerular filtration rate; AVR, aortic valve replacement; MVR, mitral valve replacement.

© 2023 Park SJ et al. *JAMA Network Open.*

eTable 8. Baseline and operative characteristics of MVR (mitral valve replacement) patients aged 40 to 54 years

	Unadjusted				IPTW-adjusted		
	Mechanical prosthesis (n = 2783)	Biological prosthesis (n = 154)	p-value	SMD	Mechanical prosthesis (n = 2783)	Biological prosthesis (n = 154)	SMD
Baseline Demographics							
Age, years	48.1±4.1	48.1±4	0.873	0.013	48.1±4.1	47.9±3.9	0.051
Female	1560 (56.1)	82 (53.2)	0.495	0.056	1557.6 (56.0)	87.1 (56.6)	0.012
Baseline conditions							
Atrial fibrillation	853 (30.7)	36 (23.4)	0.056	0.164	843.4 (30.3)	39.8 (25.8)	0.099
Hypertension	1089 (39.1)	69 (44.8)	0.161	0.115	1094.7 (39.3)	55.2 (35.8)	0.072
Diabetes mellitus	226 (8.1)	23 (14.9)	0.003	0.215	232.4 (8.4)	11.4 (7.4)	0.035
Dyslipidemia	231 (8.3)	11 (7.1)	0.611	0.043	226.2 (8.1)	13.2 (8.6)	0.016
Chronic kidney disease	56 (2.0)	17 (11.0)	<0.001	0.372	65.7 (2.4)	4.4 (2.9)	0.03
Dialysis	30 (1.1)	16 (10.4)	<0.001	0.409	40 (1.4)	3.1 (2.0)	0.046
Stroke, TIA or SE	332 (11.9)	18 (11.7)	0.928	0.007	332.9 (12.0)	25.7 (16.7)	0.136
Ischemic heart disease	419 (15.1)	26 (16.9)	0.538	0.05	421.5 (15.1)	23.5 (15.3)	0.003
Myocardial infarction	56 (2.0)	6 (3.9)	0.138	0.111	58 (2.1)	2.4 (1.6)	0.04
Previous PCI	24 (0.9)	3 (1.9)	0.166	0.092	24.8 (0.9)	0.7 (0.5)	0.056
Congestive heart failure	913 (32.8)	45 (29.2)	0.356	0.078	907.1 (32.6)	39.9 (25.9)	0.147
Anemia	249 (8.9)	36 (23.4)	<0.001	0.4	265 (9.5)	12.6 (8.2)	0.047
COPD	47 (1.7)	3 (1.9)	0.745	0.019	47.5 (1.7)	4.7 (3.1)	0.089
Asthma	289 (10.4)	17 (11.0)	0.796	0.021	289.4 (10.4)	16.6 (10.8)	0.012
Peripheral vascular disease	90 (3.2)	8 (5.2)	0.187	0.098	92.6 (3.3)	4.5 (2.9)	0.025
Previous cardiac surgery	11 (0.4)	0 (0.0)	1.000	0.089	10.4 (0.4)	0 (0.0)	0.087
Previous cancer	69 (2.5)	8 (5.2)	0.061	0.142	72.3 (2.6)	5.4 (3.5)	0.053

© 2023 Park SJ et al. *JAMA Network Open.*

Charlson comorbidity index			<0.001	0.431			0.082
0	972 (34.9)	41 (26.6)	<0.001	0.444	961.1 (34.5)	46.8 (30.4)	0.126
1	837 (30.1)	35 (22.7)			826.3 (29.7)	46 (29.9)	
2	512 (18.4)	34 (22.1)			519.9 (18.7)	35.5 (23.1)	
≥ 3	361 (13.0)	21 (13.6)			363 (13.0)	18.6 (12.1)	
≥ 5	101 (3.6)	23 (14.9)			112.7 (4.0)	7.1 (4.6)	
Years of Surgery			0.001	0.366			0.293
2002~2005	802 (28.8)	24 (15.6)			782.6 (28.1)	27.6 (17.9)	
2006~2009	725 (26.1)	55 (35.7)			740.3 (26.6)	40.6 (26.4)	
2010~2013	624 (22.4)	30 (19.5)			620.9 (22.3)	34.3 (22.3)	
2014~2018	632 (22.7)	45 (29.2			639.2 (23.0)	51.5 (33.4)	
Level of hospital		0.259	0.096				0.217
*Tertiary general hospital	2131 (76.6)	124 (80.5)			2136.7 (76.8)	103.3 (67.1)	
General hospital	652 (23.4)	30 (19.5)			646.3 (23.2)	50.7 (32.9)	
Cumulative hospital volume for cardiac surgery			<0.001	0.719			0.198
<250 cases	974 (35.0)	39 (25.3)			960.4 (34.5)	52.5 (34.1)	
250-999 cases	609 (21.9)	83 (53.9)			654.7 (23.5)	48.6 (31.6)	
1000-2999 cases	1200 (43.1)	32 (20.8)			1167.9 (42.0)	52.9 (34.4)	
Endocarditis	419 (15.1)	52 (33.8)	<0.001	0.446	444 (16.0)	32 (20.5)	0.117
Congestive heart failure	881 (31.7)	58 (37.7)	0.12	0.126	889 (31.9)	37 (24.2)	0.172
Mode of valve disease			<0.001	0.353			0.28
Mitral stenosis	1445 (51.9)	71 (46.1)			1441.4 (51.8)	73.1 (47.5)	
Mitral regurgitation	94 (3.4)	9 (5.8)			95.7 (3.4)	9.1 (5.9)	
Combined	1130 (40.6)	54 (35.1)			1123.7 (40.4)	55.1 (35.8)	
Unspecified	114 (4.1)	20 (13.0)			122.2 (4.4)	16.7 (10.8)	
Concomitant Procedure							
Tricuspid valve repair	987 (35.5)	47 (30.5)	0.211	0.105	980.1 (35.2)	44.5 (28.9)	0.135

© 2023 Park SJ et al. *JAMA Network Open.*

Coronary arterial bypass grating	75 (2.7	5 (3.2)	0.610	0.033	75.2 (2.7)	2.4 (1.6)	0.082
Surgical ablation for atrial fibrillation	1182 (42.5)	60 (39.0)	0.391	0.072	1178.9 (42.4)	69.2 (44.9)	0.052
Health Screening Data							
Height, m	162.7±8.6	163.3±8.5	0.598	0.062	162.75 (8.62)	163.8±9.4	0.113
Weight, kg	62.3±11.0	62.3±11.2	0.994	0.001	62.3±11.0	63±11.2	0.065
BMI, kg/m^2	23.5±3.2	23.3±3.3	0.654	0.052	23.4±3.2	23.4±3.0	0.017
< 18.5	55 (2.0)	4 (2.6)	0.847	0.119	55.8 (2.0)	2.6 (1.7)	0.091
≥ 18.5 and < 23	631 (22.7)	32 (20.8)			628.4 (22.6)	38.2 (24.8)	
≥ 23 and < 25	340 (12.2)	21 (13.6)			340.5 (12.2)	21.9 (14.2)	
≥ 25 and < 30	389 (14.0)	17 (11.0)			386.2 (13.9)	20.2 (13.1)	
≥ 30	42 (1.5)	3 (1.9)			42.4 (1.5)	2 (1.3)	
Not available	1326 (47.6)	77 (50.0)			1329.7 (47.8)	69 (44.8)	
Systolic blood pressure, mmHg	117.4±16.1	122.5±18.3	0.007	0.298	117.6±118.4	118.4±14.5	0.048
< 120	810 (29.1)	38 (24.7)	0.477	0.127	804.1 (28.9)	50.5 (32.8)	0.101
≥ 120 and < 140	523 (18.8)	29 (18.8)			522.2 (18.8)	26.1 (16.9)	
≥ 140	124 (4.5)	10 (6.5)			127 (4.6)	8.4 (5.5)	
Not available	1326 (47.6)	77 (50.0)			1329.7 (47.8)	69 (44.8)	
Diastolic blood pressure, mmHg	73.7±11.6	75.8±12.9	0.135	0.166	73.8±11.7	73.3±11.7	0.047
< 80	956 (34.4)	45 (29.2)			949.1 (34.1)	57.7 (37.5)	0.089
≥ 80 and < 90	378 (13.6)	23 (14.9)			379.5 (13.6)	22 (14.3)	
≥ 90	123 (4.4)	9 (5.8)			124.8 (4.5)	5.3 (3.4)	
Not available	1326 (47.6)	77 (50.0)			1329.7 (47.8)	69 (44.8)	
Smoking			0.828	0.078			0.115
Never smoker	941 (33.8)	47 (30.5)			936.7 (33.7)	52.4 (34.0)	

© 2023 Park SJ et al. *JAMA Network Open.*

Previous smoker	189 (6.8)	10 (6.5)			188.3 (6.8)	14.8 (9.6)	
Current smoker	288 (10.3)	18 (11.7)			289.6 (10.4)	17 (11.0)	
Not available	1365 (49.0)	79 (51.3)			1368.4 (49.2)	69.8 (45.3)	
Alcohol use			0.685	0.106			0.086
None	557 (20.0)	26 (16.9)			550.2 (19.8)	34 (22.1)	
Mild-to-moderate	807 (29.0)	47 (30.5)			811.1 (29.1)	45.9 (29.8)	
Heavy	59 (2.1)	2 (1.3)			57.9 (2.1)	4.2 (2.7)	
Not available	1360 (48.9)	79 (51.3)			1363.7 (49.0)	69.8 (45.3)	
Creatinine, mg/dL			<0.001	0.227			0.107
<= 1.5	884 (31.8)	42 (27.3)			878.6 (31.6)	56.4 (36.6)	
> 1.5	20 (0.7)	6 (3.9)			21.4 (0.8)	1 (0.6)	
Not available	1879 (67.5)	106 (68.8)			1882.9 (67.7)	96.6 (62.7)	
eGFR, mL/min/1.73m^2			0.026	0.172			0.161
≥ 60	648 (23.3)	33 (21.4)			646.3 (23.2)	42.1 (27.3)	
< 60	45 (1.6)	7 (4.5)			45.5 (1.6)	5.5 (3.6)	
Not available	2090 (75.1)	114 (74.0)			2091.3 (75.1)	106.5 (69.2)	

*Designated and certificated by the Ministry of Health and Welfare

Values are n (%), or mean ± standard deviation, unless otherwise indicated.

IPTW, inverse-probability-of-treatment weighting; SMD, standardized mean difference; BMI, body mass index; TIA, transient ischemic attack; SE, systemic embolization; PCI, percutaneous coronary intervention; COPD, chronic obstructive pulmonary disease; eGFR, estimated glomerular filtration rate; AVR, aortic valve replacement; MVR, mitral valve replacement.

© 2023 Park SJ et al. *JAMA Network Open.*

eTable 9. Baseline and operative characteristics of MVR (mitral valve replacement) patients aged 55 to 69 years

	Unadjusted				IPTW-adjusted		
	Mechanical prosthesis (n = 2999)	Biological prosthesis (n = 1232)	p-value	SMD	Mechanical prosthesis (n = 2999)	Biological prosthesis (n = 1232)	SMD
Baseline Demographics							
Age, years	60.8±3.9	64.9±3.6	<0.001	1.077	62.0±4.3	62.3±4.2	0.077
Female	1840 (61.4)	804 (65.3)	0.017	0.081	1868 (62.3)	773 (62.8)	0.01
Baseline conditions							
Atrial fibrillation	1305 (43.5)	457 (37.1)	<0.001	0.131	1258 (42.0)	493 (40.0)	0.04
Hypertension	1691 (56.4)	742 (60.2)	0.022	0.078	1731 (57.7)	720 (58.2)	0.015
Diabetes mellitus	539 (18.0)	300 (24.4)	<0.001	0.157	609 (20.3)	279 (22.6)	0.057
Dyslipidemia	464 (15.5)	189 (15.3)	0.915	0.004	470 (15.7)	207 (16.8)	0.032
Chronic kidney disease	63 (2.1)	55 (4.5)	<0.001	0.133	83 (2.8)	46 (3.8)	0.055
Dialysis	35 (1.2)	35 (2.8)	<0.001	0.12	49 (1.6)	27 (2.2)	0.042
Stroke, TIA or SE	480 (16.0)	210 (17.0)	0.405	0.028	493 (16.4)	212 (17.2)	0.021
Ischemic heart disease	728 (24.3)	342 (27.8)	0.018	0.079	745 (24.8)	310 (25.1)	0.007
Myocardial infarction	84 (2.8)	42 (3.4)	0.29	0.035	94 (3.1)	38 (3.0)	0.006
Previous PCI	60 (2.0)	40 (3.2)	0.015	0.078	69 (2.3)	25 (2.0)	0.017
Congestive heart failure	1351 (45.0)	613 (49.8)	0.005	0.094	1392 (46.4)	593 (48.2)	0.035
Anemia	213 (7.1)	145 (11.8)	<0.001	0.16	261 (8.7)	105 (8.5)	0.006
COPD	117 (3.9)	75 (6.1)	0.002	0.1	133 (4.4)	51 (4.1)	0.015
Asthma	487 (16.2)	236 (19.2)	0.022	0.076	511 (17.0)	218 (17.7)	0.016
Peripheral vascular disease	155 (5.2)	79 (6.4)	0.108	0.053	172 (5.7)	71 (5.8)	0.002
Previous cardiac surgery	10 (0.3)	8 (0.6)	0.151	0.045	11 (0.4)	4 (0.3)	0.012
Previous cancer	124 (4.1)	70 (5.7)	0.029	0.072	139 (4.6)	57 (4.6)	<0.001

© 2023 Park SJ et al. *JAMA Network Open.*

Charlson comorbidity index			<0.001	0.241			0.072
0	618 9 (20.6)	174 (14.1)	<0.001	0.238	557 (18.6)	197 (16.0)	0.083
1	838 (27.9)	311 (25.2)			806 (26.9)	330 (26.8)	
2	640 (21.3)	277 (22.5)			650 (21.7)	266 (21.6)	
≥ 3	637 (21.2)	294 (23.9)			664 (22.2)	286 (23.2)	
≥ 5	266 (8.9)	176 (14.3)			321 (10.7)	153 (12.4)	
Years of Surgery			0.032	0.1			0.007
2002~2005	763 (25.4)	278 (22.6)			731 (24.4)	299 (24.3)	
2006~2009	694 (23.1)	331 (26.9)			731 (24.4)	300 (24.3)	
2010~2013	662 (22.1)	255 (20.7)			642 (21.4)	267 (21.7)	
2014~2018	880 (29.3)	368 (29.9)			895 (29.8)	366 (29.7)	
Level of hospital			<0.001	0.152			0.003
*Tertiary general hospital	2302 (76.8)	863 (70.0)			2255.2 (75.2)	925 (75.1)	
General hospital	697 (23.2)	369 (30.0)			743.8 (24.8)	307 (24.9)	
Cumulative hospital volume for cardiac surger y			<0.001	0.297			0.136
<250 cases	1011 (33.7)	505 (41.0)			1080 (36.0)	473 (38.4)	
250-999 cases	630 (21.0)	344 (27.9)			703 (23.4)	337 (27.3)	
1000-2999 cases	1358 (45.3)	383 (31.1)			1217 (40.6)	422 (34.2)	
Endocarditis	328 (10.9)	203 (16.5)	<0.001	0.162	371 (12.4)	160 (13.0)	0.019
Congestive heart failure	1176 (39.2)	513 (41.6)	0.143	0.049	1196 (39.9)	512 (41.5)	0.034
Mode of valve disease			<0.001	0.174			0.074
Mitral stenosis	1588 (53.0)	629 (51.1)			1582.5 (52.8)	617.3 (50.1)	
Mitral regurgitation	80 (2.7)	61 (5.0)			89.9 (3.0)	38 (3.1)	
Combined	1223 (40.8)	466 (37.8)			1201.9 (40.1)	509.3 (41.3)	
Unspecified	108 (3.6)	76 (6.2)			124.7 (4.2)	67.5 (5.5)	
Concomitant Procedure							

© 2023 Park SJ et al. *JAMA Network Open.*

Tricuspid valve repair	1385 (46.2)	526 (42.7)	0.038	0.07	1330 (44.4)	513 (41.6)	0.055
Coronary arterial bypass grating	176 (5.9)	100 (8.1)	0.007	0.088	201 (6.7)	82 (6.6)	0.004
Surgical ablation for atrial fibrillation	1388 (46.3)	552 (44.8)	0.381	0.03	1353 (45.1)	544 (44.2)	0.019
Health Screening Data							
Height, m	159.4±8.4	157.6 (8.1)	<0.001	0.211	159.1±8.5	158.1±8.3	0.121
Weight, kg	60.4±10.5	58.2±9.9	<0.001	0.22	59.9±10.5	59.0±10.3	0.086
BMI, kg/m^2	23.7±3.2	23.4±3.3	0.013	0.107	23.6±3.2	23.6±3.4	0.009
< 18.5	64 (2.1)	36 (2.9)	0.12	0.1	74.9 (2.5)	31.7 (2.6)	0.038
≥ 18.5 and < 23	748 (24.9)	320 (26.0)			761 (25.4)	310 (25.2)	
≥ 23 and < 25	470 (15.7)	189 (15.3)			460 (15.3)	182 (14.8)	
≥ 25 and < 30	547 (18.2)	188 (15.3)			518 (17.3)	202 (16.4)	
≥ 30	71 (2.4)	24 (1.9)			69 (2.3)	27 (2.2)	
Not available	1099 (36.6)	475 (38.6)			1116 (37.2)	479 (38.8)	
Systolic blood pressure, mmHg	120.6±16.3	122.6±17.3	0.005	0.124	121.4±16.8	121.9±17.2	0.026
< 120	868 (28.9)	305 (24.8)	0.009	0.114	828 (27.6)	322 (26.1)	0.043
≥ 120 and < 140	803 (26.8)	330 (26.8)			794 (26.5)	318 (25.8)	
≥ 140	229 (7.6)	121 (9.8)			261 (8.7)	112 (9.1)	
Not available	1099 (36.6)	476 (38.6)			1116 (37.2)	479 (38.9)	
Diastolic blood pressure, mmHg	74.8±10.9	75.4±11.2	0.194	0.056	75.1±11.0	75.3±11.1	0.015
< 80	1172(39.1)	440 (35.7)	0.158	0.077	1134 (37.8)	446 (36.12)	0.04
≥ 80 and < 90	531 (17.7)	221 (17.9)			536 (17.9)	216 (17.5)	
≥ 90	197 (6.6)	95 (7.7)			212 (7.1)	91 (7.4)	
Not available	1099 (36.6)	476 (38.6)			1116 (37.2)	479 (38.9)	
Smoking			0.011	0.115			0.072

© 2023 Park SJ et al. *JAMA Network Open.*

Never smoker	1366 (45.5)	565 (45.9)			1371 (45.7)	572 (46.4)	
Previous smoker	254 (8.5)	76 (6.2)			230 (7.7)	77 (6.3)	
Current smoker	246 (8.2)	83 (6.7)			231 (7.7)	82 (6.7)	
Not available	1133 (37.8)	508 (41.2)			1167 (28.9)	501 (40.6)	
Alcohol use			0.007	0.119			0.042
None	939 (31.3)	394 (32.0)			937 (31.2)	379 (30.7)	
Mild-to-moderate	875 (29.2)	299 (24.3)			836 (27.9)	326 (26.4)	
Heavy	54 (1.8)	29 (2.4)			59 (2.0)	27 (2.2)	
Not available	1131 (37.7)	510 (41.4)			1167 (38.9)	501 (40.7)	
Creatinine, mg/dL			0.001	0.119			0.06
<= 1.5	1237 (41.2)	472 (38.2)			1207 (40.2)	462 (37.5)	
> 1.5	34 (1.1)	32 (2.6)			44 (1.5)	22 (1.8)	
Not available	1728 (57.6)	728 (59.1)			1748 (58.3)	748 (60.7)	
eGFR, mL/min/1.73m^2			<0.001	0.161			0.072
≥ 60	842 (28.1)	296 (24.0)			816 (27.2)	299 (24.2)	
< 60	164 (5.5)	114 (9.3)			189 (6.3)	88 (7.1)	
Not available	1993 (66.5)	8227 (66.7)			1994 (66.5)	846 (68.6)	

*Designated and certificated by the Ministry of Health and Welfare

Values are n (%), or mean ± standard deviation, unless otherwise indicated.

IPTW, inverse-probability-of-treatment weighting; SMD, standardized mean difference; BMI, body mass index; TIA, transient ischemic attack; SE, systemic embolization; PCI, percutaneous coronary intervention; COPD, chronic obstructive pulmonary disease; eGFR, estimated glomerular filtration rate; AVR, aortic valve replacement; MVR, mitral valve replacement.

© 2023 Park SJ et al. *JAMA Network Open.*

eTable 10. Baseline and operative characteristics of MVR (mitral valve replacement) patients aged 70 to 79 years

	Unadjusted				IPTW-adjusted		
	Mechanical prosthesis (n = 175)	Biological prosthesis (n = 1568)	p-value	SMD	Mechanical prosthesis (n = 175)	Biological prosthesis (n = 1568)	SMD
Baseline Demographics							
Age, years	72.5±2.3	73.7±2.7	<0.001	0.451	73.2±2.5	73.6±5.7	0.131
Female	107 (61.1)	1099 (70.1)	0.015	0.189	124.5 (71.1)	1086.9 (69.3)	0.04
Baseline conditions							
Atrial fibrillation	84 (48.0)	711 (45.3)	0.504	0.053	70.9 (40.5)	713.3 (45.5)	0.1
Hypertension	129 (73.7)	1143 (72.9)	0.817	0.019	138.3 (79.0)	1146.4 (73.1)	0.139
Diabetes mellitus	53 (30.3)	488 (31.1)	0.82	0.018	54.2 (31.0)	485.8 (31.0)	<0.001
Dyslipidemia	27 (15.4)	333 (21.2)	0.072	0.151	32.6 (18.6)	325.1 (20.7)	0.053
Chronic kidney disease	7 (4.0)	76 (4.8)	0.618	0.041	5.8 (3.3)	74.8 (4.8)	0.075
Dialysis	1 (0.6)	29 (1.8)	0.355	0.117	1.4 (0.8)	27 (1.7)	0.082
Stroke, TIA or SE	29 (16.6)	302 (19.3)	0.39	0.07	31.2 (17.8)	297.9 (19.0)	0.03
Ischemic heart disease	68 (38.9)	549 (35.0)	0.313	0.08	65 (37.1)	554.4 (35.4)	0.038
Myocardial infarction	9 (5.1)	61 (3.9)	0.423	0.06	9.4 (5.4)	63.4 (4.0)	0.064
Previous PCI	8 (4.6)	83 (5.3)	0.684	0.033	11.5 (6.6)	81.3 (5.2)	0.058
Congestive heart failure	101 (57.7)	861 (54.9)	0.479	0.057	98.3 (56.2)	866.3 (55.2)	0.018
Anemia	24 (13.7)	222 (14.2)	0.873	0.013	22.4 (12.8)	221.6 (14.1)	0.039
COPD	16 (9.1)	138 (8.8)	0.88	0.012	24.1 (13.8)	139.9 (8.9)	0.153
Asthma	41 (23.4)	352 (22.4)	0.769	0.023	48.2 (27.5)	356.3 (22.7)	0.112
Peripheral vascular disease	18 (10.3)	122 (7.8)	0.247	0.087	11.8 (6.7)	124.8 (8.0)	0.047
Previous cardiac surgery	2 (1.1)	14 (0.9)	0.671	0.025	0.7 (0.4)	14.1 (0.9)	0.062

© 2023 Park SJ et al. *JAMA Network Open.*

Previous cancer	7 (4.0)	131 (8.4)	0.043	0.182	13.9 (7.9)	124.3 (7.9)	0.002
Charlson comorbidity index			0.384	0.075			0.008
0	27 (15.4)	161 (10.3)	0.044	0.248	19.5 (11.1)	168.1 (10.7)	0.19
1	25 (14.3)	322 (20.5)			27.5 (15.7)	311.3 (19.9)	
2	44 (25.1_	317 (20.2)			38.9 (22.2)	325 (20.7)	
≥ 3	53 (30.3)	483 (30.8)			65.6 (37.5)	483.9 (30.9)	
≥ 5	26 (14.9)	285 (18.2)			23.6 (13.5)	279.6 (17.8)	
Years of Surgery			<0.001	0.327			0.074
2002~2005	39 (22.3)	179 (11.4)			19.5 (11.1)	197.6 (12.6)	
2006~2009	42 (24.0)	334 (21.3)			42.5 (24.3)	339 (21.6)	
2010~2013	39 (22.3)	426 (27.2)			47.1 (26.9)	418 (26.7)	
2014~2018	55 (31.4)	629 (40.1)			65.9 (37.7)	613.4 (39.1)	
Level of hospital			0.471	0.057			0.062
*Tertiary general hospital	124 (70.9)	1151 (73.4)			123.3 (70.5)	1149 (73.3)	
General hospital	51 (29.1)	417 (26.6)			51.7 (29.5)	419 (26.7)	
Cumulative hospital volume for cardiac surgery			0.953	0.025			0.152
<250 cases	73 (41.7)	635 (40.5)			63.6 (36.3)	633.7 (40.4)	
250-999 cases	41 (23.4)	376 (24.0)			36.1 (20.6)	375.5 (23.9)	
1000-2999 cases	61 (34.9)	557 (35.5)			75.3 (43.0)	558.9 (35.6)	
Endocarditis	28 (16.0)	229 (14.6)	0.621	0.039	26 (15.0)	230 (14.7)	0.009
Congestive heart failure	88 (50.3)	704 (44.9)	0.175	0.108	83 (47.7)	713 (45.4)	0.045
Mode of valve disease			0.519	0.119			0.118
Mitral stenosis	87	863			101.7	853.0	
Mitral regurgitation	6	42			6.6	40.2	
Combined	71	558			57.3	570.9	
Unspecified	11	105			9.3	103.9	
Concomitant Procedure							

© 2023 Park SJ et al. *JAMA Network Open.*

Tricuspid valve repair	78 (44.6)	726 (46.3)	0.663	0.035	85 (48.5)	725 (46.3)	0.045
Coronary arterial bypass grating	18 (10.3)	193 (12.3)	0.436	0.064	20 (11.3)	190 (12.1)	0.028
Surgical ablation for atrial fibrillation	52 (29.7)	625 (39.9)	0.009	0.214	73 (41.5)	609 (38.7)	0.054
Health Screening Data							
Height, m	156.6±8.1	156.1±8.5	0.621	0.054	154.8±7.4	156.2±8.5	0.168
Weight, kg	58.3±11.1	57.7±9.8	0.632	0.049	56.6±9.3	57.7±9.8	0.122
BMI, kg/m^2	23.7±3.5	23.6±3.3	0.966	0.004	23.5±3.0	23.6±3.3	0.036
< 18.5	5 (2.9)	35 (2.2)	0.099	0.252	3.1 (1.8)	35.8 (2.3)	0.098
≥ 18.5 and < 23	40 (22.9)	403 (25.7)			43.1 (24.6)	399.1 (25.5)	
≥ 23 and < 25	14 (8.0)	220 (14.0)			25.1 (14.3)	210.9 (13.5)	
≥ 25 and < 30	30 (17.1)	295 (18.8)			30.5 (17.4)	292.7 (18.7)	
≥ 30	6 (3.4)	37 (2.4)			2.5 (1.4)	37.2 (2.4)	
Not available	80 (45.7)	578 (36.9)			70.7 (40.4)	592.2 (37.8)	
Systolic blood pressure, mmHg	122.2±13.8	125.7±16.1	0.043	0.231	126.1±14.7	125.5 (16.1)	0.042
< 120	34 (19.4)	315 (20.1)	0.056	0.231	30.2 (17.3)	313.7 (20.0)	0.085
≥ 120 and < 140	49 (28.0)	477 (30.4)			51 (29.1)	472.2 (30.1)	
≥ 140	12 (6.9)	195 (12.4)			23.1 (13.2)	187 (11.9)	
Not available	80 (45.7)	581 (37.1)			70.7 (40.4)	595.1 (38.0)	
Diastolic blood pressure, mmHg	73.6±8.9	75.2±10.8	0.104	0.161	74.8±10.0	75.0±10.8	0.021
< 80	63 (36.0)	576 (36.7)	0.024	0.273	63 (36.2)	574 (36.6)	0.08
≥ 80 and < 90	28 (16.0)	300 (19.1)			32 (18.5)	295 (18.8)	
≥ 90	4 (2.3)	111 (7.1)			9 (5.0)	104 (6.6)	
Not available	80 (45.7)	581 (37.1)			71 (40.4)	595 (38.0)	
Smoking			0.165	0.179			0.086

© 2023 Park SJ et al. *JAMA Network Open.*

Never smoker	76 (43.4)	814 (51.9)			88 (50.4)	802 (51.2)	
Previous smoker	12 (6.9)	105 (6.7)			9 (4.9)	104 (6.7)	
Current smoker	7 (4.0)	63 (4.0)			8 (4.3)	62 (4.0)	
Not available	80 (45.7)	586 (37.4)			71 (40.4)	599 (38.2)	
Alcohol use			0.104	0.21			0.052
None	64 (36.6)	672(42.9)			73 (41.7)	661 (42.2)	
Mild-to-moderate	30 (17.1)	275 (17.5)			28 (15.9)	275 (17.5)	
Heavy	1 (0.6)	33 (2.1)			4 (2.1)	31 (2.0)	
Not available	80 (45.7)	588 (37.5)			71 (40.4)	601 (38.3)	
Creatinine, mg/dL			0.094	0.178			0.11
<= 1.5	70 (40.0)	740 (47.2)			80 (45.4)	727 (46.4)	
> 1.5	2 (1.1)	35 (2.2)			2 (0.9)	33 (2.1)	
Not available	103 (58.9)	793 (50.6)			94 (53.8)	807 (51.5)	
eGFR, mL/min/1.73m^2			0.29	0.128			0.095
≥ 60	39 (22.3)	424 (27.0)			47 (27.0)	416 (26.5)	
< 60	20 (11.4)	199 (12.7)			17 (9.5)	195 (12.4)	
Not available	116 (66.3)	945 (60.3)			111 (63.5)	957 (61.0)	

*Designated and certificated by the Ministry of Health and Welfare

Values are n (%), or mean ± standard deviation, unless otherwise indicated.

IPTW, inverse-probability-of-treatment weighting; SMD, standardized mean difference; BMI, body mass index; TIA, transient ischemic attack; SE, systemic embolization; PCI, percutaneous coronary intervention; COPD, chronic obstructive pulmonary disease; eGFR, estimated glomerular filtration rate; AVR, aortic valve replacement; MVR, mitral valve replacement.

© 2023 Park SJ et al. *JAMA Network Open.*

eTable 11. Baseline and operative characteristics of DVR (double valve replacement) patients aged 40 to 54 years

	Unadjusted				IPTW-adjusted		
	Mechanical prosthesis (n = 1163)	Biological prosthesis (n = 43)	p-value	SMD	Mechanical prosthesis (n = 1163)	Biological prosthesis (n = 43)	SMD
Baseline Demographics							
Age, years	48.1±4.1	49.0±4.0	0.167	0.217	48.2±4.1	47.8±4.4	0.085
Female	658 (56.6)	18 (41.9)	0.056	0.298	652.1 (56.1)	23.6 (54.9)	0.025
Baseline conditions							
Atrial fibrillation	354 (30.4)	5 (11.6)	0.008	0.474	346.6 (29.8)	12.4 (28.8(	0.021
Hypertension	500 (43.0)	14 (32.6)	0.174	0.216	496.2 (42.7)	18.2 (42.3)	0.005
Diabetes mellitus	78 (6.7)	3 (7.0)	0.763	0.011	77.5 (6.7)	5 (11.6)	0.176
Dyslipidemia	94 (8.1)	1 (2.3)	0.248	0.261	90.8 (7.8)	0.1 (0.2)	0.401
Chronic kidney disease	19 (1.6)	4 (9.3)	0.008	0.342	21.4 (1.8)	0.4 (0.9)	0.078
Dialysis	13 (1.1)	4 (9.3)	0.002	0.375	15.6 (1.3)	0.4 (0.9)	0.039
Stroke, TIA or SE	99 (8.5)	3 (70.)	1.000	0.057	97.8 (8.4)	1.5 (3.5)	0.208
Ischemic heart disease	226 (19.4)	4 (9.3)	0.097	0.292	222.4 (19.1)	7.8 (18.1)	0.025
Myocardial infarction	21 (1.8)	0 (0.0)	1.000	0.192	20.3 (1.7)	0 (0.0)	0.188
Previous PCI	11 (0.9)	0 (0.0)	1.000	0.138	10.6 (0.9)	0 (0.0)	0.136
Congestive heart failure	431 (37.1)	13 (30.2)	0.362	0.145	426.8 (36.7)	10.1 (23.5)	0.293
Anemia	90 (7.7)	6 (14.0)	0.146	0.201	92.6 (8.0)	4.2 (9.8)	0.064
COPD	18 (1.5)	1 (2.3)	0.501	0.056	17.8 (1.5)	0.1 (0.2)	0.141
Asthma	114 (9.8)	4 (9.3)	1.000	0.017	113.6 (9.8)	3.1 (7.2)	0.088
Peripheral vascular disease	34 (2.9)	0 (0.0)	0.630	0.245	32.8 (2.8)	0 (0.0)	0.241
Previous cardiac surgery	0 (0.0)	1 (2.3)	0.036	0.218	0 (0.0)	0.1 (0.2)	0.051
Previous cancer	17 (1.5)	1 (2.3)	0.482	0.063	17.2 (1.5)	0.2 (0.5)	0.113

© 2023 Park SJ et al. *JAMA Network Open.*

Charlson comorbidity index			0.560	0.192			0.103
0	430 (37.0)	15 (34.9)	0.426	0.249	430 (37.0)	19.5 (45.3)	0.293
1	383 (32.9)	14 (32.6)			382.7 (32.9)	9.5 (22.1)	
2	185 (15.9)	5 (11.6)			184.4 (15.9)	8 (18.6)	
≥ 3	136 (11.7)	6 (14.0)			136.4 (11.7)	5.7 (13.3)	
≥ 5	29 (2.5)	3 (7.0)			29.5 (2.5)	0.3 (0.7)	
Years of Surgery			0.733	0.176			0.167
2002~2005	398 (34.2)	12 (27.9)			394.8 (33.9)	11.5 (26.7)	
2006~2009	334 (28.7)	15 (34.9)			336.9 (29.0)	14.9 (34.7)	
2010~2013	216 (18.6)	7 (16.3)			215.9 (18.6)	8.2 (19.1)	
2014~2018	215 (18.5)	9 (20.9)			215.4 (18.5)	8.4 (19.5)	
Level of hospital			0.286	0.177			0.160
*Tertiary general hospital	923 (79.4)	37 (86.0)			924.1 (79.5)	36.8 (85.6)	
General hospital	240 (20.6)	6 (14.0)			238.9 (20.5)	6.2 (14.4)	
Cumulative hospital volume for cardiac surgery (AVR)			<0.001	0.81			0.501
<250	237 (20.4)	11 (25.6)			238.9 (20.5)	11.9 (27.7)	
250-999	319 (27.4)	24 (55.8)			331.1 (28.5)	17.2 (40.0)	
1000-2999	416 (35.8)	7 (16.3)			407.7 (35.1)	12.5 (29.1)	
≥3000	191 (16.4)	1 (2.3)			185.4 (15.9)	1.4 (3.3)	
Cumulative hospital volume for cardiac surgery (MVR)			<0.001	0.813			0.399
<250	290 (24.9)	12 (27.9)			291.1 (25.0)	12.8 (29.8)	
250-1000	266 (22.9)	23 (53.5)			278.9 (24.0)	16.2 (37.7)	
1000-3000	607 (52.2)	8 (18.6)			593 (51.0)	13.9 (32.3)	
Endocarditis	174 (15.0)	17 (39.5)	<0.001	0.574	184.6 (15.9)	8.2 (19.1)	0.085

© 2023 Park SJ et al. *JAMA Network Open.*

Congestive heart failure	433 (37.2)	19 (44.2)	0.355	0.142	437.4 (37.6)	19.9 (46.3)	0.178
Bicuspid aortic valve	12 (1.0)	0 (0.0)	1.000	0.144	11.6 (1.0)	0 (0.0)	0.142
Mode of valve disease							
Aortic stenosis	171 (14.7)	5 (11.6)	0.129	0.375	168.6 (14.5)	11.2 (26.0)	0.446
Aortic regurgitation	384 (33.0)	21 (48.8)			386.9 (33.3)	17.4 (40.5)	
Combined	435 (37.4)	10 (23.3)			431.7 (37.1)	11.8 (27.4)	
Unspecified	173 (14.9)	7 (16.3)			175.8 (15.1)	2.5 (5.8)	
Mitral stenosis	603 (51.8)	20 (46.5)	0.015	0.428	599.6 (51.6)	21.9 (51.0)	0.031
Mitral regurgitation	41 (3.5)	4 (9.3)			42.9 (3.7)	1.8 (4.2)	
Combined	423 (36.4)	11 (25.6)			420.3 (36.1)	15.4 (35.8)	
Unspecified	96 (8.3)	8 (18.6)			100.2 (8.6)	3.9 (9.1)	
Concomitant Procedure							
Tricuspid valve repair	421 (36.2)	13 (30.2)	0.423	0.127	418.2 (36.0)	12.3(28.6)	0.158
Coronary arterial bypass grating	16 (1.4)	4 (9.3)	0.004	0.358	17.5 (1.5)	0.5 (1.2)	0.031
Surgical ablation for atrial fibrillation	451 (38.8)	7 (16.3)	0.003	0.521	441.8 (38.0)	10.7 (24.9)	0.286
Health Screening Data							
Height, m	162.5±8.6	162.9±8.6	0.830	0.049	162.5±7.9	165.9±9.7	0.38
Weight, kg	61.4±11.0	63.5±11.0	0.410	0.173	61.5±10.8	64.4±11.6	0.259
BMI, kg/m^2	23.2±3.2	23.9±3.2	0.488	0.181	23.2 (3.22)	23.3±3.1	0.028
< 18.5	29 (2.5)	1 (2.3)	0.075	0.519	28.8 (2.5)	1.1 (2.6)	0.646
≥ 18.5 and < 23	283 (24.3)	7 (16.3)			281.3 (24.2)	6.2 (14.4)	
≥ 23 and < 25	151 (13.0)	9 (20.9)			151.1 (13.0)	12.8 (29.8)	
≥ 25 and < 30	144 (12.4)	1 (2.3)			142.6 (12.3)	0.4 (0.9)	
≥ 30	16 (1.4)	2 (4.7)			15.8 (1.4)	0.7 (1.6)	
Not available	540 (46.4)	23 (53.5)			543.4 (46.7)	21.7 (50.5)	

© 2023 Park SJ et al. *JAMA Network Open.*

Systolic blood pressure, mmHg	118.3±16.1	118.8±16.1	0.913	0.029	118.3±15.7	118.3±18.4	0.001
< 120	325 (27.9)	10 (23.3)	0.639	0.2	323.1 (27.8)	9.2 (21.4)	0.185
≥ 120 and < 140	244 (21.0)	7 (16.3)			242.2 (20.8)	10.8 (25.1)	
≥ 140	54 (4.6)	3 (7.0)			54.3 (4.7)	1.3 (3.0)	
Not available	540 (46.4)	23 (53.5)			543.4 (46.7)	21.7 (50.5)	
Diastolic blood pressure, mmHg	72.0±11.6	72.7±11.6	0.778	0.06	72.0±10.8	72.9±10.4	0.081
< 80	430 (37.0)	13 (30.2)	0.130	0.336	428.3(36.8)	13.1 (30.5)	0.134
≥ 80 and < 90	151 (13.0)	3 (7.0)			149.6 (12.9)	6.4 (14.9)	
≥ 90	42 (3.6)	4 (9.3)			41.7 (3.6)	1.7 (4.0)	
Not available	540 (46.4)	23 (53.5)			543.4 (46.7)	21.7 (50.5)	
Smoking			0.355	0.29			0.312
Never smoker	416 (35.8)	10 (23.3)			412.7 (35.5)	11.6 (27.0)	
Previous smoker	85 (7.3)	4 (9.3)			85 (7.3)	6.8(15.8)	
Current smoker	110 (9.5)	6 (14.0)			110.1 (9.5)	2.9 (6.7)	
Not available	552 (47.5)	23 (53.5)			555.2 (47.7)	21.7 (50.5)	
Alcohol use			0.446	0.282			0.289
None	217 (18.7)	4 (9.3)			214.7 (18.5)	4.5 (10.5)	
Mild-to-moderate	374 (32.2)	14 (32.6)			372.9 (32.1)	16.2 (37.7)	
Heavy	21 (1.8)	1 (2.3)			21.2 (1.8)	0.1 (0.2)	
Not available	551 (47.4)	24 (55.8)			554.3 (47.7)	22.2 (51.6)	
Creatinine, mg/dL			0.125	0.241			0.042
<= 1.5	337 (29.0)	9 (20.9)			334.9 (28.8)	12.6 (29.3)	
> 1.5	5 (0.4)	1 (2.3)			4.8 (0.4)	0.1 (0.2)	
Not available	821 (70.6)	33 (76.7)			823.3 (70.8)	30.3 (70.5)	
eGFR, mL/min/1.73m^2			0.750	0.123			0.239

© 2023 Park SJ et al. *JAMA Network Open.*

≥ 60	245 (21.1)	7 (16.3)	242.9 (20.9)	6.6 (15.3)
< 60	25 (2.1)	1 (2.3)	25.2 (2.2)	0.1 (0.2)
Not available	893 (76.8)	35 (81.4)	894.9 (76.9)	36.3 (84.4)

*Designated and certificated by the Ministry of Health and Welfare

Values are n (%), or mean ± standard deviation, unless otherwise indicated.

IPTW, inverse-probability-of-treatment weighting; SMD, standardized mean difference; BMI, body mass index; TIA, transient ischemic attack; SE, systemic embolization; PCI, percutaneous coronary intervention; COPD, chronic obstructive pulmonary disease; eGFR, estimated glomerular filtration rate; AVR, aortic valve replacement; MVR, mitral valve replacement.

© 2023 Park SJ et al. *JAMA Network Open.*

eTable 12. Baseline and operative characteristics of DVR (double valve replacement) patients aged 55 to 64 years

	Unadjusted				IPTW-adjusted		
	Mechanical prosthesis (n = 996)	Biological prosthesis (n = 134)	p-value	SMD	Mechanical prosthesis (n = 996)	Biological prosthesis (n = 134)	SMD
Baseline Demographics							
Age, years	59.2±2.8	60.7±2.9	<0.001	0.527	59.4±2.8	59.7±3.2	0.114
Female	565 (56.7)	72 (53.7)	0.512	0.06	561.7 (56.4)	73.3 (54.7)	0.034
Baseline conditions							
Atrial fibrillation	378 (38.0)	41 (30.6)	0.098	0.155	370.3 (37.2)	49 (36.6)	0.013
Hypertension	567 (56.9)	74 (55.2)	0.709	0.034	565.3 (56.8)	70.9 (52.9)	0.077
Diabetes mellitus	168 (16.9)	28 (20.9)	0.248	0.103	173.4 (17.4)	22.1 (16.5)	0.025
Dyslipidemia	143 (14.4)	20 (14.9)	0.861	0.016	144.6 (14.5)	21.4 (16.0)	0.041
Chronic kidney disease	32 (3.2)	12 (9.0)	0.001	0.242	36.9 (3.7)	4.9 (3.7)	0.001
Dialysis	22 (2.2)	9 (6.7)	0.007252	0.22	25.7 (2.6)	3.6 (2.7)	0.008
Stroke, TIA or SE	145 (14.6)	23 (17.2)	0.426	0.071	148.9 (14.9)	18.9 (14.1)	0.023
Ischemic heart disease	244 (24.5)	41 (30.6)	0.127	0.137	250.1 (25.1)	31.8 (23.7)	0.032
Myocardial infarction	27 (2.7)	6 (4.5)	0.268987	0.095	28.9 (2.9)	3.7 (2.8)	0.01
Previous PCI	16 (1.6)	5 (3.7)	0.092545	0.132	17.8 (1.8)	2.2 (1.6)	0.011
Congestive heart failure	448 (45.0)	58 (43.3)	0.711	0.034	447.6 (44.9)	59.1 (44.1)	0.016
Anemia	77 (7.7)	29 (21.6)	<0.001	0.401	89.3 (9.0)	11.1 (8.3)	0.024
COPD	35 (3.5)	3 (2.2)	0.611456	0.076	33.1 (3.3)	1.7 (1.3)	0.139
Asthma	123 (12.3)	21 (15.7)	0.279	0.096	127.1 (12.8)	14.8 (11.0)	0.052
Peripheral vascular disease	48 (4.8)	9 (6.7)	0.346	0.081	49.6 (5.0)	5.6 (4.2)	0.038
Previous cardiac surgery	2 (0.2)	0 (0.0)	1	0.063	1.8 (0.2)	0 (0.0)	0.06
Previous cancer	35 (3.5)	10 (7.5)	0.028	0.174	39.6 (4.0)	8.5 (6.3)	0.107

© 2023 Park SJ et al. *JAMA Network Open.*

Charlson comorbidity index			0.025	0.274			0.053
0	221 (22.2)	33 (24.6)	0.002	0.389	224.5 (22.5)	36 (26.9)	0.224
1	308 (30.9)	22 (16.4)			290.5 (29.2)	26.5 (19.8)	
2	206 (20.7)	28 (20.9)			207.3 (20.8)	31.4 (23.4)	
≥ 3	187 (18.8)	32 (23.9)			190 (19.1)	28.8 (21.5)	
≥ 5	74 (7.4)	19 (14.2)			83.7 (8.4)	11.4 (8.5)	
Years of Surgery			0.176	0.214			0.114
2002~2005	268 (26.9)	38 (28.4)			269.9 (27.1)	43.2 (32.2)	
2006~2009	235 (23.6)	39 (29.1)			239.2 (24.0)	30.5 (22.8)	
2010~2013	227 (22.80	20 (14.9)			217.3 (21.8)	27.2 (20.3)	
2014~2018	266 (26.7)	37 (27.6)			269.6 (27.1)	33 (24.6)	
Level of hospital			0.009	0.23			0.11
*Tertiary general hospital	797 (80.0)	94 (70.1)			791.3 (79.4)	100.3 (74.9)	
General hospital	199 (20.0)	40 (29.9)			204.7 (20.6)	33.7 (25.1)	
Cumulative hospital volume for cardiac surgery (AVR)			<0.001	0.459			0.244
<250	208 (20.9)	40 (29.9)			214.5 (21.5)	26.7 (19.9)	
250-999	267 (26.8)	46 (34.3)			281.6 (28.3)	52.8 (39.4)	
1000-2999	338 (33.9)	41 (30.6)			331.7 (33.3)	35.2 (26.3)	
≥3000	183 (18.4)	7 (5.2)			168.3 (16.9)	19.3 (14.4)	
Cumulative hospital volume for cardiac surgery (MVR)			0.002	0.337			0.256
<250	254 (25.5)	45 (33.6)			258.7 (26.0)	32.4 (24.2)	
250-999	221 (22.2)	41 (30.6)			237.4 (23.8)	47.1 (35.1)	
1000-2999	521 (52.3)	48 (35.8)			499.9 (50.2)	54.5 (40.7)	
Endocarditis	156 (15.7)	33 (24.6)	0.009	0.225	170.2 (17.1)	27.3 (20.4)	0.085
Congestive heart failure	391 (39.3)	56 (41.8)	0.573	0.052	394 (39.6)	59.4 (44.3)	0.097

© 2023 Park SJ et al. *JAMA Network Open.*

Bicuspid aortic valve	20 (2.0)	2 (1.5)	1.000	0.039	19.3 (1.9)	1.1 (0.8)	0.097
Mode of valve disease							
Aortic stenosis	221 (22.2)	19 (14.2)	0.036	0.275	216 (21.7)	20.6 (15.4)	0.242
Aortic regurgitation	266 (26.7)	46 (34.3)			273.1 (27.4)	48.5 (36.2)	
Combined	371 (37.2)	44 (32.8)			366.6 (36.8)	42.6 (31.8)	
Unspecified	138 (13.9)	25 (18.7)			140.2 (14.1)	22.3 (16.6)	
Mitral stenosis	502 (50.4)	66 (49.3)	0.764	0.095	492.9 (49.5)	60.9 (45.4)	0.109
Mitral regurgitation	28 (2.8)	4 (3.0)			29.1 (2.9)	6.1 (4.6)	
Combined	362 (36.3)	46 (34.3)			364.4 (36.6)	52 (38.8)	
Unspecified	104 (10.4)	18 (13.4)			109.5 (11.0)	15 (11.2)	
Concomitant Procedure							
Tricuspid valve repair	406 (40.8)	58 (43.3)	0.578	0.051	405.4 (40.7)	48.6 (36.3)	0.091
Coronary arterial bypass grating	37 (3.7)	5 (3.7)	1	0.001	37.5 (3.8)	6.6 (4.9)	0.056
Surgical ablation for atrial fibrillation	406 (40.8)	54 (40.3)	0.918	0.009	405.5 (40.7)	50.3 (37.5)	0.065
Health Screening Data							
Height, m	159.7±7.9	160.1±7.1	0.673	0.054	159.7±7.9	159.3±7.5	0.055
Weight, kg	59.9±9.8	58.2±9.5	0.15	0.179	59.7±9.9	58.0±8.0	0.185
BMI, kg/m^2	23.4±2.8	22.7±3.3	0.041	0.236	23.3±2.9	22.9±3.3	0.123
< 18.5	21 (2.1)	7 (5.2)	0.057	0.295	24.2 (2.4)	6.3 (4.7)	0.353
≥ 18.5 and < 23	238 (23.9)	37 (27.6)			243 (24.4)	31.4 (23.4)	
≥ 23 and < 25	170 (17.1)	13 (9.7)			159.2 (16.0)	8.1 (6.0)	
≥ 25 and < 30	154 (15.5)	16 (11.9)			153.3 (15.4)	23.2 (17.3)	
≥ 30	12 (1.2)	2 (1.5)			11.6 (1.2)	0.9 (0.7)	
Not available	401 (40.3)	59 (44.0)			404.7 (40.6)	64.1 (47.8)	
Systolic blood pressure, mmHg	120.3±15.8	124.7±15.8	0.023	0.278	120.9±16.2	124.0±15.4	0.194

© 2023 Park SJ et al. *JAMA Network Open.*

< 120	271 (27.2)	22 (16.4)	0.057	0.269	260.9 (26.2)	30 (22.4)	0.271
≥ 120 and < 140	252 (25.3)	42 (31.3)			255.2 (25.6)	23.4 (17.5)	
≥ 140	71 (7.1)	11 (8.2)			74.2 (7.4)	16.5 (12.3)	
Not available	402 (40.4)	59 (44.0)			405.6 (40.7)	64.1 (47.8)	
Diastolic blood pressure, mmHg	72.4±10.3	75.2±9.0	0.024	0.291	72.8 (10.62)	74.3±9.4	0.141
< 80	407 (40.9)	41 (30.6)	0.104	0.23	393.2 (39.5)	40.3 (30.1)	0.205
≥ 80 and < 90	148 (14.9)	27 (20.1)			156 (15.7)	22.2 (16.6)	
≥ 90	39 (3.9)	7 (5.2)			41.2 (4.1)	7.5 (5.6)	
Not available	402 (40.4)	59 (44.0)			405.6 (40.7)	64.1 (47.8)	
Smoking			0.355	0.182			0.162
Never smoker	420 (42.2)	58 (43.3)			421.7 (42.3)	50 (37.3)	
Previous smoker	98 (9.8)	7 (5.2)			93 (9.3)	9.1 (6.8)	
Current smoker	72 (7.2)	9 (6.7)			71.8 (7.2)	10.6 (7.9)	
Not available	406 (40.8)	60 (44.8)			409.5 (41.1)	64.3 (48.0)	
Alcohol use			0.388	0.151			0.222
None	275 (27.6)	32 (23.9)			271.5 (27.3)	32 (23.9)	
Mild-to-moderate	292 (29.3)	36 (26.9)			286 (28.7)	29.9 (22.3)	
Heavy	25 (2.5)	6 (4.5)			30.7 (3.1)	7.8 (5.8)	
Not available	404 (40.6)	60 (44.8)			407.7 (40.9)	64.3 (48.0)	
Creatinine, mg/dL			0.045	0.236			0.129
<= 1.5	367 (36.8)	35 (26.1)			359 (36.0)	41.7 (31.1)	
> 1.5	14 (1.4)	3 (2.2)			13.8 (1.4)	0.9 (0.7)	
Not available	615 (61.7)	96 (71.6)			623.2 (62.6)	91.4 (68.2)	
eGFR, mL/min/1.73m^2			0.208	0.17			0.079
≥ 60	263 (26.4)	26 (19.4)			258.8 (26.0)	30.5 (22.8)	
< 60	39 (3.9)	5 (3.7)			37.6 (3.8)	5.9 (4.4)	

© 2023 Park SJ et al. *JAMA Network Open.*

Not available	694 (69.7)	103 (76.9)	699.6 (70.2)	97.6 (72.8)

*Designated and certificated by the Ministry of Health and Welfare

Values are n (%), or mean ± standard deviation, unless otherwise indicated.

IPTW, inverse-probability-of-treatment weighting; SMD, standardized mean difference; BMI, body mass index; TIA, transient ischemic attack; SE, systemic embolization; PCI, percutaneous coronary intervention; COPD, chronic obstructive pulmonary disease; eGFR, estimated glomerular filtration rate; AVR, aortic valve replacement; MVR, mitral valve replacement.

© 2023 Park SJ et al. *JAMA Network Open.*

eTable 13. Baseline and operative characteristics of DVR (double valve replacement) patients aged 65 to 79 years

	Unadjusted				IPTW-adjusted		
	Mechanical prosthesis (n = 304)	Biological prosthesis (n = 830)	p-value	SMD	Mechanical prosthesis (n = 304)	Biological prosthesis (n = 830)	SMD
Baseline Demographics							
Age, years	67.7±2.7	71.7±3.8	<0.001	1.235	69.8±3.4	70.7±3.9	0.247
Female	172 (56.6)	505 (60.8)	0.195	0.087	188.5 (62.0)	505.2 (60.9)	0.024
Baseline conditions							
Atrial fibrillation	138(45.4)	317 (38.2)	0.028	0.146	114.7 (37.7)	331.5 (39.9)	0.045
Hypertension	203 (66.8)	577 (69.5)	0.377	0.059	217.1 (71.4)	569.4 (68.6)	0.062
Diabetes mellitus	67 (22.0)	206 (24.8)	0.332	0.066	77.8 (25.6)	198.2 (23.9)	0.04
Dyslipidemia	47 (15.5)	168 (20.2)	0.069	0.125	62.9 (20.7)	160.6 (19.3)	0.033
Chronic kidney disease	14 (4.6)	34 (4.1)	0.706	0.025	14 (4.6)	33.1 (4.0)	0.03
Dialysis	6 (2.0)	20 (2.4)	0.664	0.03	9.3 (3.1)	18.4 (2.2)	0.053
Stroke, TIA or SE	46 (15.1)	135 (16.3)	0.644	0.031	41 (13.5)	134 (16.1)	0.075
Ischemic heart disease	91 (29.9)	274 (33.0)	0.326	0.066	97.3 (32.0)	259.5 (31.3)	0.016
Myocardial infarction	12 (3.9)	25 (3.0)	0.432	0.051	16.1 (5.3)	29.4 (3.5)	0.085
Previous PCI	10 (3.3)	37 (4.5)	0.382	0.061	14.7 (4.8)	34 (4.1)	0.035
Congestive heart failure	163 (53.6)	433 (52.2)	0.665	0.029	174.7 (57.5)	438 (52.8)	0.094
Anemia	36 (11.8)	99 (11.9)	0.969	0.003	31.7 (10.4)	98.8 (11.9)	0.047
COPD	14 (4.6)	66 (8.0)	0.051	0.138	13.5 (4.4)	57.4 (6.9)	0.106
Asthma	51 (16.8)	199 (24.0)	0.010	0.179	56.2 (18.5)	188.6 (22.7)	0.105
Peripheral vascular disease	24 (7.9)	60 (70.2)	0.705	0.025	25 (8.2)	57.7 (7.0)	0.048
Previous cardiac surgery	1 (0.3)	5 (0.6)	1.000	0.04	0.9 (0.3)	4.8 (0.6)	0.044
Previous cancer	16 (5.3)	59 (7.1)	0.268	0.077	22.1 (7.3)	55 (6.6)	0.025

© 2023 Park SJ et al. *JAMA Network Open.*

Charlson comorbidity index			0.292	0.089			0.004
0	35 (11.5)	120 (14.5)	0.071	0.197	36.4 (12.0)	116.4 (14.0)	0.14
1	90 (29.6)	189 (22.8)			72 (23.7)	194.2 (23.4)	
2	72 (23.7)	177 (21.3)			82 (27.0)	185.8 (22.4)	
≥ 3	72 (23.7)	222 (26.7)			68.3 (22.5)	219.9 (26.5)	
≥ 5	35 (11.5)	122 (14.7)			45.3 (14.9)	113.8 (13.7)	
Years of Surgery			0.017	0.21			0.036
2002~2005	68 (22.4)	123 (14.8)			52.1 (17.1)	138.5 (16.7)	
2006~2009	59 (19.4)	166 (20.2)			63.2 (20.8)	169 (20.4)	
2010~2013	66 (21.7)	225 (27.1)			81.1 (26.7)	214.6 (25.9)	
2014~2018	111 (36.5)	316 (38.1)			107.6 (35.4)	308 (37.1)	
Level of hospital			0.001	0.236			0.065
*Tertiary general hospital	258 (84.9)	627 (75.5)			241.2 (79.3)	636 (76.6)	
General hospital	46 (15.1)	203 (24.5)			62.8 (20.7)	194 (23.4)	
Cumulative hospital volume for cardiac surgery (AVR)			<0.001	0.309			0.076
<250	66 (21.7)	227 (27.3)			81.9 (26.9)	222.6 (26.8)	
250-999	82 (27.0)	228 (27.5)			81.8 (26.9)	225.9 (27.2)	
1000-2999	87 (28.6)	279 (33.6)			89.6 (29.5)	264 (31.78)	
≥3000	69 (22.7)	96 (11.6)			50.6 (16.6)	117.5 (14.2)	
Cumulative hospital volume for cardiac surgery (MVR)			0.007	0.216			0.074
<250	74 (24.3)	283 (34.1)			89.9 (29.6)	268.5 (32.3)	
250-999	74 (24.3)	172 (20.7)			73.8 (24.3)	179.9 (21.7)	
1000-2999	156 (51.3)	375 (45.2)			140.2 (46.1)	381.5 (46.0)	
Endocarditis	33 (10.9)	155 (18.7)	0.002	0.222	40.3 (13.3)	135.7 (16.3)	0.087
Congestive heart failure	121 (39.8)	387 (46.6)	0.041	0.138	144.8 (47.6)	372.4 (44.9)	0.055

© 2023 Park SJ et al. *JAMA Network Open.*

Bicuspid aortic valve							
Mode of valve disease							
Aortic stenosis	77 (25.3)	241 (29.0)	0.032	0.199	74.8 (24.6)	244.2 (29.4)	0.237
Aortic regurgitation	59 (19.4)	202 (24.3)			54.7 (18.0)	202 (24.3)	
Combined	128 (42.1)	273 (32.9)			133.1 (43.8)	278.3 (33.5)	
Unspecified	40 (13.2)	114 (13.7)			41.4 (13.6)	105.4 (12.7)	
Mitral stenosis	159 (52.3)	442 (53.3)	0.003	0.262	150.9 (49.6)	430.9 (51.9)	0.219
Mitral regurgitation	9 (3.0)	34 (4.1)			7.5 (2.5)	31.9 (3.8)	
Combined	116 (38.2)	246 (29.6)			123.3 (40.6)	268.4 (32.3)	
Unspecified	20 (6.6)	108 (13.0)			22.3 (7.3)	98.7 (11.9)	
Concomitant Procedure							
Tricuspid valve repair	132 (43.4)	294 (35.4)	0.014	0.164	123.2 (40.5)	310.9 (37.5)	0.063
Coronary arterial bypass grating	14 (4.6)	53 (6.4)	0.260	0.078	25.6 (8.4)	53.6 (6.5)	0.075
Surgical ablation for atrial fibrillation	116 (38.2)	306 (36.9)	0.690	0.027	115 (37.8)	311.9 (37.6)	0.005
Health Screening Data							
Height, m	159.2±8.5	157.3±8.6	0.008	0.226	158.9±8.4	157.5±8.5	0.165
Weight, kg	58.7±9.2	57.7±9.5	0.209	0.106	58.6±9.7	57.7±9.3	0.097
BMI, kg/m^2	23.1±3.0	23.3±3.1	0.537	0.053	23.1±2.8	23.2±3.1	0.031
< 18.5	9 (3.0)	32 (3.9)	0.770	0.111	11.4 (3.8)	30.1 (3.6)	0.097
≥ 18.5 and < 23	76 (25.0)	204 (24.6)			82.1 (27.0)	207.6 (25.0)	
≥ 23 and < 25	60 (19.7)	153 (18.4)			54.1 (17.8)	152.7 (18.4)	
≥ 25 and < 30	48 (15.8)	116 (14.0)			44.9 (14.8)	117.8 (14.2)	
≥ 30	2 (0.7)	12 (1.4)			1.3 (0.4)	9.5 (1.1)	
Not available	109 (35.9)	313 (37.7)			110.2 (36.3)	312.2 (37.6)	
Systolic blood pressure, mmHg	123.9±15.6	125.1±18.0	0.371	0.073	124.8±14.8	124.6±17.6	0.013

© 2023 Park SJ et al. *JAMA Network Open.*

< 120	76 (25.0)	177 (21.3)	0.400	0.116	69.2 (22.8)	188.3 (22.7)	0.034
≥ 120 and < 140	91 (29.9)	242 (29.2)			91.4 (30.1)	238.4 (28.7)	
≥ 140	28 (9.2)	98 (11.8)			33.2 (10.9)	91.1 (11.0)	
Not available	109 (35.9)	313 (37.7			110.2 (36.3)	312.2 (37.6)	
Diastolic blood pressure, mmHg	73.8±11.0	73.2±11.1	0.500	0.057	73.8 (10.4)	73.3±10.9	0.044
< 80	124 (40.8)	336 (40.5)	0.876	0.055	121.6 (40.0)	337.1 (40.6)	0.049
≥ 80 and < 90	55 (18.1)	135 (16.3)			55.4 (18.2)	137.6 (16.6)	
≥ 90	16 (5.3)	46 (5.5)			16.7 (5.5)	43 (5.2)	
Not available	109 (35.9)	313 (37.7)			110.2 (36.3)	312.2 (37.6)	
Smoking			0.658	0.083			0.071
Never smoker	137 (45.1)	391 (47.1)			150.3 (49.4)	391.4 (47.2)	
Previous smoker	28 (9.2)	69 (8.3)			25.9 (8.5)	68.2 (8.2)	
Current smoker	21 (6.9)	43 (5.2)			12 (3.9)	43.3 (5.2)	
Not available	118 (38.8)	327 (39.4)			115.8 (38.1)	327.1 (39.4)	
Alcohol use			0.772	0.07			0.076
None	116 (38.2)	335 (40.4)			126.7 (41.7)	329.5 (39.7)	
Mild-to-moderate	63 (20.7)	150 (18.1)			57.1 (18.8)	155.2 (18.7)	
Heavy	7 (2.3)	20 (2.4)			4.4 (1.4)	19.6 (2.4)	
Not available	118 (38.8)	325 (39.2)			115.8 (38.1)	325.6 (39.2)	
Creatinine, mg/dL			0.791	0.044			0.188
<= 1.5	141 (46.4)	389 (46.9)			138 (45.4)	387.7 (46.7)	
> 1.5	7 (2.3)	14 (1.7)			14.1 (4.6)	11.9 (1.4)	
Not available	156 (51.3)	427 (51.4)			151.9 (50.0)	430.5 (51.9)	
eGFR, mL/min/1.73m^2			0.917	0.028			0.056
≥ 60	89 (29.3)	233 (28.1)			80.4 (26.4)	237.1 (28.6)	
< 60	37 (12.2)	105 (12.7)			38.3 (12.6)	94.1 (11.3)	

© 2023 Park SJ et al. *JAMA Network Open.*

Not available	178 (58.6)	492 (59.3)	185.3 (61.0)	498.7 (60.1)

*Designated and certificated by the Ministry of Health and Welfare

Values are n (%), or mean ± standard deviation, unless otherwise indicated.

IPTW, inverse-probability-of-treatment weighting; SMD, standardized mean difference; BMI, body mass index; TIA, transient ischemic attack; SE, systemic embolization; PCI, percutaneous coronary intervention; COPD, chronic obstructive pulmonary disease; eGFR, estimated glomerular filtration rate; AVR, aortic valve replacement; MVR, mitral valve replacement.

© 2023 Park SJ et al. *JAMA Network Open.*

eTable 14. Comparative Outcomes of Mechanical versus Bio-prosthetic DVR (double valve replacement) using competing-risk analysis

	Original		IPTW-adjusted			
	HR (95% CI)	P-value	Mechanical prosthesis	Biological prosthesis	aHR (95% CI)	P-value
**Age < 55 (N=1206)**			1163	43		
Death	2.97 (1.65-5.38)	<0.001	135	8	1.64 (0.66-4.05)	0.284
Cardiovascular death	2.91 (1.41-6.01)	0.004	86	6	2.01 (0.68-5.91)	0.206
Non-Cardiovascular death	2.54 (0.92-6.99)	0.071	49	1	0.88 (0.21-3.63)	0.857
Valve related events						
Reoperation	8.35 (3.89-17.92)	<0.001	31	6	5.62 (1.82-17.35)	0.003
Thromboembolism	1.37 (0.49-3.82)	0.550	88	3	1.05 (0.26-4.30)	0.950
Major bleeding	0.54 (0.07-3.97)	0.541	51	1	0.39 (0.05-3.02)	0.369
**55 ≤ Age ≤64 (N=1130)**			996	134		
Death	2.20 (1.61-3.00)	<0.001	190	46	2.02 (1.28-3.19)	0.002
Cardiovascular death	1.64 (1.08-2.51)	0.022	121	31	2.06 (1.17-3.64)	0.013
Non-Cardiovascular death	2.75 (1.72-4.38)	<0.001	69	15	1.62 (0.84-3.14)	0.149
Valve related events						
Reoperation	5.75 (3.35-9.86)	<0.001	30	26	7.13 (3.86-13.18)	<0.001
Thromboembolism	0.28 (0.10-0.75)	0.011	99	4	0.25 (0.08-0.78)	0.017
Major bleeding	1.53 (0.79-2.96)	0.203	53	12	1.75 (0.77-3.95)	0.180

© 2023 Park SJ et al. *JAMA Network Open.*

Age ≥ 65 (N=1134)			304	830		
Death	1.22 (0.98-1.51)	0.070	119	331	0.95 (0.71-1.28)	0.750
Cardiovascular death	1.17 (0.89-1.54)	0.253	79	218	0.96 (0.65-1.43)	0.842
Non-Cardiovascular death	1.14 (0.81-1.61)	0.456	40	113	1.00 (0.61-1.64)	0.993
Valve related events						
Reoperation	3.29 (1.29-8.38)	0.013	3	53	6.65 (2.27-19.50)	0.001
Thromboembolism	0.62 (0.41-0.94)	0.025	31	56	0.65 (0.36-1.14)	0.131
Major bleeding	0.70 (0.34-1.46)	0.341	6	19	1.22 (0.56-2.65)	0.618

IPTW, inverse-probability-of-treatment weighting; aHR, adjusted hazard ratio; CI, confidence interval.

© 2023 Park SJ et al. *JAMA Network Open.*

eTable 15. The Cause of Death Information after AVR (aortic valve replacement)

		**Aortic Valve Replacement** (N=11993)			
		**Mechanical** N=4825		**Bioprosthetic** N=7168	
**Cause of Death**	**ICD-10 codes**	N	(%)	N	(%)
**Number of Death**		822	(100.0)	2019	(100.0)
Malignant neoplasm	C00-97	133	(16.2)	357	(17.7)
Endocrine, nutritional and metabolic diseases	E00-E90	34	(4.1)	88	(4.4)
Diseases of the circulatory system	I00-I99	401	(48.8)	926	(45.9)
Rheumatic mitral valve diseases	I05	2	(0.5)	0	(0.0)
Rheumatic aortic valve diseases	I06	9	(2.2)	16	(1.7)
Multiple valve diseases of specified origin other than rheumatic heart disease	I08	10	(2.5)	5	(0.5)
Hypertensive heart disease	I11	5	(1.2)	19	(2.1)
Angina pectoris	I20	8	(2.0)	20	(2.2)
Acute myocardial infarction	I21	52	(13.0)	93	(10.0)
Chronic ischaemic heart disease	I25	14	(3.5)	48	(5.2)
Acute and subacute endocarditis	I33	31	(7.7)	70	(7.6)
Nonrheumatic mitral valve disorders	I34	1	(0.2)	2	(0.2)
Nonrheumatic aortic valve disorders	I35	93	(23.2)	292	(31.5)
Endocarditis, valve unspecified	I38	14	(3.5)	25	(2.7)
Cardiomyopathy complicating in the puerperium	I42	7	(1.7)	7	(0.8)
Cardiac arrest	I46	15	(3.7)	25	(2.7)

© 2023 Park SJ et al. *JAMA Network Open.*

Atrial fibrillation and flutter	I48	4	(1.0)	14	(1.5)
Other cardiac arrhythmias	I49	5	(1.2)	9	(1.0)
Heart failure	I50	25	(6.2)	69	(7.5)
Complications and ill-defined descriptions of heart disease	I51	2	(0.5)	12	(1.3)
Subarachnoid haemorrhage	I60	5	(1.2)	1	(0.1)
Intracerebral haemorrhage	I61	52	(13.0)	42	(4.5)
Other nontraumatic intracranial haemorrhage	I62	9	(2.2)	5	(0.5)
Cerebral infarction	I63	15	(3.7)	60	(6.5)
Sequelae of cerebrovascular disease	I69	8	(2.0)	32	(3.5)
Aortic aneurysm and dissection	I71	3	(0.7)	17	(1.8)
Other I codes		12	(3.0)	43	(4.6)
Diseases of the respiratory system	J00-J99	47	(5.7)	172	(8.5)
Diseases of the digestive system	K00-K93	27	(3.3)	72	(3.6)
Diseases of the genitourinary system	N00-N99	40	(4.9)	89	(4.4)
Symptoms, signs and abnormal clinical and laboratory findings, NEC	R00-R99	33	(4.0)	89	(4.4)
Injury, poisoning and certain other consequences of external causes	S00-T98	51	(6.2)	85	(4.2)

**According to Age-strata**	**ICD-10 codes**	**N**	**(%)**	**N**	**(%)**
**40-54 years old**		N=1654		N=150	
**Number of Death**		185	(100.0)	32	(100.0)
Malignant neoplasm	C00-97	24	(13.0)	6	(18.8)
Endocrine, nutritional and metabolic diseases	E00-E90	6	(3.2)	1	(3.1)
Diseases of the circulatory system	I00-I99	94	(50.8)	21	(65.6)

© 2023 Park SJ et al. *JAMA Network Open.*

Diseases of the respiratory system	J00-J99	5	(2.7)	0	(0.0)
Diseases of the digestive system	K00-K93	9	(4.9)	2	(6.3)
Diseases of the genitourinary system	N00-N99	4	(2.2)	1	(3.1)
Symptoms, signs and abnormal clinical and laboratory findings, NEC	R00-R99	10	(5.4)	0	(0.0)
Injury, poisoning and certain other consequences of external causes	S00-T98	20	(10.8)	0	(0.0)
Others		13	(7.0)	1	(3.1)
**55-64 years old**		N=2227		N=773	
**Number of Death**		324	(100.0)	141	(100.0)
Malignant neoplasm	C00-97	59	(18.2)	18	(12.8)
Endocrine, nutritional and metabolic diseases	E00-E90	16	(4.9)	6	(4.3)
Diseases of the circulatory system	I00-I99	150	(46.3)	76	(53.9)
Diseases of the respiratory system	J00-J99	22	(6.8)	6	(4.3)
Diseases of the digestive system	K00-K93	10	(3.1)	7	(5.0)
Diseases of the genitourinary system	N00-N99	18	(5.6)	5	(3.5)
Symptoms, signs and abnormal clinical and laboratory findings, NEC	R00-R99	9	(2.8)	4	(2.8)
Injury, poisoning and certain other consequences of external causes	S00-T98	16	(4.9)	9	(6.4)
Others		24	(7.4)	10	(7.1)
**65-79 years old**		N=944		N=6245	
**Number of Death**		313	(100.0)	1846	(100.0)
Malignant neoplasm	C00-97	50	(16.0)	333	(18.0)
Endocrine, nutritional and metabolic diseases	E00-E90	12	(3.8)	81	(4.4)
Diseases of the circulatory system	I00-I99	157	(50.2)	829	(44.9)

© 2023 Park SJ et al. *JAMA Network Open.*

Diseases of the respiratory system	J00-J99	20	(6.4)	166	(9.0)
Diseases of the digestive system	K00-K93	8	(2.6)	63	(3.4)
Diseases of the genitourinary system	N00-N99	18	(5.8)	83	(4.5)
Symptoms, signs and abnormal clinical and laboratory findings, NEC	R00-R99	14	(4.5)	85	(4.6)
Injury, poisoning and certain other consequences of external causes	S00-T98	15	(4.8)	76	(4.1)
Others		19	(6.1)	130	(7.0)

© 2023 Park SJ et al. *JAMA Network Open.*

eTable 16. The Cause of Death Information after MVR (mitral valve replacement)

		**Mitral Valve Replacement** (N=8911)			
		**Mechanical** N=5957		**Bioprosthetic** N=2954	
**Cause of Death**	**ICD-10 codes**	N	(%)	N	(%)
**Number of Death**		985	(100.0)	1074	(100.0)
Malignant neoplasm	C00-97	101	(10.3)	106	(9.9)
Endocrine, nutritional and metabolic diseases	E00-E90	38	(3.9)	37	(3.4)
Diseases of the circulatory system	I00-I99	557	(56.5)	659	(61.4)
Rheumatic mitral valve diseases	I05	105	(18.9)	106	(16.1)
Rheumatic aortic valve diseases	I06	0	(0.0)	1	(0.2)
Multiple valve diseases of specified origin other than rheumatic heart disease	I08	17	(3.1)	28	(4.2)
Hypertensive heart disease	I11	8	(1.4)	13	(2.0)
Angina pectoris	I20	3	(0.5)	5	(0.8)
Acute myocardial infarction	I21	35	(6.3)	63	(9.6)
Chronic ischaemic heart disease	I25	15	(2.7)	23	(3.5)
Acute and subacute endocarditis	I33	40	(7.2)	49	(7.4)
Nonrheumatic mitral valve disorders	I34	52	(9.3)	91	(13.8)
Nonrheumatic aortic valve disorders	I35	3	(0.5)	2	(0.3)
Endocarditis, valve unspecified	I38	26	(4.7)	37	(5.6)
Cardiomyopathy complicating in the puerperium	I42	10	(1.8)	15	(2.3)
Cardiac arrest	I46	18	(3.2)	9	(1.4)

© 2023 Park SJ et al. *JAMA Network Open.*

Atrial fibrillation and flutter	I48	15	(2.7)	10	(1.5)
Other cardiac arrhythmias	I49	8	(1.4)	6	(0.9)
Heart failure	I50	35	(6.3)	50	(7.6)
Complications and ill-defined descriptions of heart disease	I51	7	(1.3)	6	(0.9)
Subarachnoid haemorrhage	I60	7	(1.3)	3	(0.5)
Intracerebral haemorrhage	I61	54	(9.7)	39	(5.9)
Other nontraumatic intracranial haemorrhage	I62	11	(2.0)	7	(1.1)
Cerebral infarction	I63	37	(6.6)	50	(7.6)
Sequelae of cerebrovascular disease	I69	26	(4.7)	23	(3.5)
Aortic aneurysm and dissection	I71	2	(0.4)	3	(0.5)
Other I codes		23	(4.1)	20	(3.0)
Diseases of the respiratory system	J00-J99	57	(5.8)	62	(5.8)
Diseases of the digestive system	K00-K93	36	(3.7)	24	(2.2)
Diseases of the genitourinary system	N00-N99	28	(2.8)	55	(5.1)
Symptoms, signs and abnormal clinical and laboratory findings, NEC	R00-R99	30	(3.0)	34	(3.2)
Injury, poisoning and certain other consequences of external causes	S00-T98	79	(8.0)	29	(2.7)

**According to Age-strata**	**ICD-10 codes**	**N**	**(%)**	**N**	**(%)**
**40-54 years old**		N=2783		N=154	
**Number of Death**		290	(100.0)	29	(100.0)
Malignant neoplasm	C00-97	23	(7.9)	11	(37.9)
Endocrine, nutritional and metabolic diseases	E00-E90	23	(7.9)	3	(37.9)
Diseases of the circulatory system	I00-I99	166	(57.2)	6	(20.7)

© 2023 Park SJ et al. *JAMA Network Open.*

Diseases of the respiratory system	J00-J99	13	(4.5)	0	(0.0)
Diseases of the digestive system	K00-K93	8	(2.8)	0	(0.0)
Diseases of the genitourinary system	N00-N99	9	(3.1)	4	(13.8)
Symptoms, signs and abnormal clinical and laboratory findings, NEC	R00-R99	7	(2.4)	1	(3.4)
Injury, poisoning and certain other consequences of external causes	S00-T98	32	(11.0)	1	(3.4)
Others		9	(3.1)	3	(10.3)
**55-64 years old**		N=2391		N=463	
**Number of Death**		613	(100.0)	393	(100.0)
Malignant neoplasm	C00-97	76	(12.4)	50	(12.7)
Endocrine, nutritional and metabolic diseases	E00-E90	26	(4.2)	19	(4.8)
Diseases of the circulatory system	I00-I99	338	(55.1)	231	(58.8)
Diseases of the respiratory system	J00-J99	34	(5.5)	19	(4.8)
Diseases of the digestive system	K00-K93	24	(3.9)	10	(2.5)
Diseases of the genitourinary system	N00-N99	16	(2.6)	17	(4.3)
Symptoms, signs and abnormal clinical and laboratory findings, NEC	R00-R99	22	(3.6)	9	(2.3)
Injury, poisoning and certain other consequences of external causes	S00-T98	43	(7.0)	12	(3.1)
Others		34	(5.5)	26	(6.6)
**65-79 years old**		N=783		N=2337	
**Number of Death**		82	(100.0)	652	(100.0)
Malignant neoplasm	C00-97	2	(2.4)	45	(6.9)
Endocrine, nutritional and metabolic diseases	E00-E90	0	(0.0)	15	(2.3)
Diseases of the circulatory system	I00-I99	53	(64.6)	422	(64.7)

© 2023 Park SJ et al. *JAMA Network Open.*

Diseases of the respiratory system	J00-J99	10	(12.2)	43	(6.6)
Diseases of the digestive system	K00-K93	4	(4.9)	14	(2.1)
Diseases of the genitourinary system	N00-N99	3	(3.7)	34	(5.2)
Symptoms, signs and abnormal clinical and laboratory findings, NEC	R00-R99	1	(1.2)	24	(3.7)
Injury, poisoning and certain other consequences of external causes	S00-T98	4	(4.9)	16	(2.5)
Others		5	(6.1)	39	(6.0)

© 2023 Park SJ et al. *JAMA Network Open.*

eTable 17. The Cause of Death Information after DVR (double valve replacement)

		**Double Valve Replacement** (N=3470)			
		**Mechanical** N=2463		**Bioprosthetic** N=1007	
**Cause of Death**	**ICD-10 codes**	N	(%)	N	(%)
**Number of Death**		428	(100.0)	398	(100.0)
Malignant neoplasm	C00-97	45	(10.5)	45	(11.3)
Endocrine, nutritional and metabolic diseases	E00-E90	8	(1.9)	12	(3.0)
Diseases of the circulatory system	I00-I99	272	(63.6)	248	(62.3)
Rheumatic mitral valve diseases	I05	36	(13.2)	12	(4.8)
Rheumatic aortic valve diseases	I06	2	(0.7)	1	(0.4)
Multiple valve diseases of specified origin other than rheumatic heart disease	I08	39	(14.3)	31	(12.5)
Hypertensive heart disease	I11	3	(1.1)	4	(1.6)
Angina pectoris	I20	1	(0.4)	0	(0.0)
Acute myocardial infarction	I21	14	(5.1)	10	(4.0)
Chronic ischaemic heart disease	I25	2	(0.7)	4	(1.6)
Acute and subacute endocarditis	I33	19	(7.0)	36	(14.5)
Nonrheumatic mitral valve disorders	I34	10	(3.7)	13	(5.2)
Nonrheumatic aortic valve disorders	I35	21	(7.7)	34	(13.7)
Endocarditis, valve unspecified	I38	17	(6.3)	24	(9.7)
Cardiomyopathy complicating in the puerperium	I42	2	(0.7)	3	(1.2)
Cardiac arrest	I46	7	(2.6)	4	(1.6)

© 2023 Park SJ et al. *JAMA Network Open.*

Atrial fibrillation and flutter	I48	2	(0.7)	4	(1.6)
Other cardiac arrhythmias	I49	3	(1.1)	3	(1.2)
Heart failure	I50	15	(5.5)	22	(8.9)
Complications and ill-defined descriptions of heart disease	I51	1	(0.4)	6	(2.4)
Subarachnoid haemorrhage	I60	7	(2.6)	4	(1.6)
Intracerebral haemorrhage	I61	27	(9.9)	8	(3.2)
Other nontraumatic intracranial haemorrhage	I62	9	(3.3)	1	(0.4)
Cerebral infarction	I63	13	(4.8)	8	(3.2)
Sequelae of cerebrovascular disease	I69	7	(2.6)	4	(1.6)
Aortic aneurysm and dissection	I71	4	(1.5)	1	(0.4)
Other I codes		11	(4.0)	11	(4.4)
Diseases of the respiratory system	J00-J99	14	(3.3)	19	(4.8)
Diseases of the digestive system	K00-K93	11	(2.6)	12	(3.0)
Diseases of the genitourinary system	N00-N99	13	(3.0)	18	(4.5)
Symptoms, signs and abnormal clinical and laboratory findings, NEC	R00-R99	20	(4.7)	8	(2.0)
Injury, poisoning and certain other consequences of external causes	S00-T98	25	(5.8)	14	(3.5)

**According to Age-strata**	**ICD-10 codes**	**N**	**(%)**	**N**	**(%)**
**40-54 years old**		N=1163		N=43	
**Number of Death**		131	(100.0)	12	(100.0)
Malignant neoplasm	C00-97	13	(100.0)	1	(8.3)
Endocrine, nutritional and metabolic diseases	E00-E90	2	(15.4)	1	(8.3)
Diseases of the circulatory system	I00-I99	83	(638.5)	8	(66.7)

© 2023 Park SJ et al. *JAMA Network Open.*

Diseases of the respiratory system	J00-J99	4	(30.8)	0	(0.0)
Diseases of the digestive system	K00-K93	3	(23.1)	0	(0.0)
Diseases of the genitourinary system	N00-N99	5	(38.5)	0	(0.0)
Symptoms, signs and abnormal clinical and laboratory findings, NEC	R00-R99	6	(46.2)	1	(8.3)
Injury, poisoning and certain other consequences of external causes	S00-T98	9	(69.2)	0	(0.0)
Others		6	(4.6)	1	(8.3)
**55-64 years old**		N=996		N=134	
**Number of Death**		187	(100.0)	50	(100.0)
Malignant neoplasm	C00-97	23	(12.3)	6	(12.0)
Endocrine, nutritional and metabolic diseases	E00-E90	5	(2.7)	1	(2.0)
Diseases of the circulatory system	I00-I99	120	(64.2)	26	(52.0)
Diseases of the respiratory system	J00-J99	4	(2.1)	4	(8.0)
Diseases of the digestive system	K00-K93	2	(1.1)	0	(0.0)
Diseases of the genitourinary system	N00-N99	5	(2.7)	3	(6.0)
Symptoms, signs and abnormal clinical and laboratory findings, NEC	R00-R99	7	(3.7)	0	(0.0)
Injury, poisoning and certain other consequences of external causes	S00-T98	11	(5.9)	7	(14.0)
Others		10	(5.3)	3	(6.0)
**65-79 years old**		N=304		N=830	
**Number of Death**		110	(100.0)	336	(100.0)
Malignant neoplasm	C00-97	9	(8.2)	38	(11.3)
Endocrine, nutritional and metabolic diseases	E00-E90	1	(0.9)	10	(3.0)
Diseases of the circulatory system	I00-I99	69	(62.7)	214	(63.7)

© 2023 Park SJ et al. *JAMA Network Open.*

Diseases of the respiratory system	J00-J99	6	(5.5)	15	(4.5)
Diseases of the digestive system	K00-K93	6	(5.5)	12	(3.6)
Diseases of the genitourinary system	N00-N99	3	(2.7)	15	(4.5)
Symptoms, signs and abnormal clinical and laboratory findings, NEC	R00-R99	7	(6.4)	7	(2.1)
Injury, poisoning and certain other consequences of external causes	S00-T98	5	(4.5)	7	(2.1)
Others		4	(3.6)	18	(5.4)

© 2023 Park SJ et al. *JAMA Network Open.*

eTable 18. Comparative Outcomes of Mechanical versus Bio-prosthetic AVR (aortic valve replacement) without competing-risk analysis

	**Original**		**IPTW-adjusted**			
	HR (95% CI)	P- value	Mechanical prosthesis	Biological prosthesis	aHR (95% CI)	P-value
**Age < 55 (N=1804)**						
Cardiovascular death	3.37 (2.09-5.43)	<0.001	93	15	2.07 (1.12-3.83)	0.002
Non-Cardiovascular death	2.11 (1.23-3.96)	0.020	93	15	2.32 (1.04-5.18)	0.040
Valve related events						
Reoperation	5.34 (2.69-10.60)	<0.001	35	9	3.59 (1.62-7.96)	0.005
Thromboembolism	0.86 (0.38-1.96)	0.724	117	4	0.50 (0.18-1.38)	0.178
Major bleeding	2.98 (1.25-7.13)	0.014	35	5	1.95 (0.65-5.91)	
**55 ≤ Age < 65 (N=3000)**						
Cardiovascular death	1.79 (1.35-2.36)	<0.001	150	73	1.49 (1.07-2.08)	0.019
Non-Cardiovascular death	1.39 (1.04-1.85)	0.024	174	63	1.11 (0.80-1.56)	0.534
Valve related events						
Reoperation	2.87 (1.74-4.73)	<0.001	35	32	2.89 (1.62-5.13)	<0.001
Thromboembolism	0.71 (0.49-1.02)	0.063	173	34	0.57 (0.38-0.87)	0.009
Major bleeding	0.66 (0.37-1.17)	0.157	74	14	0.55 (0.29-1.06)	0.072
**Age ≥ 65 (N=7189)**						
Cardiovascular death	0.98 (0.83-1.16)	0.819	188	814	0.66 (0.52-0.83)	<0.001
Non-Cardiovascular death	1.29 (1.09-1.53)	0.003	175	1020	0.90 (0.72-1.13)	0.357

© 2023 Park SJ et al. *JAMA Network Open.*

Valve related events						
Reoperation	2.00 (1.01-3.98)	0.047	5	103	2.92 (1.22-6.99)	0.016
Thromboembolism	0.64 (0.52-0.80)	<0.001	109	375	0.52 (0.39-0.69)	<0.001
Major bleeding	0.31 (0.22-0.43)	<0.001	38	90	0.37 (0.24-0.57)	<0.001

IPTW, inverse-probability-of-treatment weighting; aHR, adjusted hazard ratio; CI, confidence interval.

© 2023 Park SJ et al. *JAMA Network Open.*

eTable 19. Comparative Outcomes of Mechanical versus Bio-prosthetic MVR (mitral valve replacement) without competing-risk analysis

	**Original**		**IPTW-adjusted**			
	HR (95% CI)	P- value	Mechanical prosthesis	Biological prosthesis	aHR (95% CI)	P-value
**Age < 55 (N=2937)**			2783	154		
Cardiovascular death	0.74 (0.33-1.67)	0.470	170	3	0.36 (0.14-0.88)	0.025
Non-Cardiovascular death	3.96 (2.54-6.19)	<0.001	131	14	2.23 (1.14-4.35)	0.019
Valve related events						
Reoperation	8.59 (5.30-13.90)	<0.001	66	14	4.86 (2.62-9.03)	<0.001
Thromboembolism	0.64 (0.29-1.45)	0.286	201	8	0.84 (0.27-2.58)	0.761
Major bleeding	0.64 (0.20-2.01)	0.444	101	3	0.66 (0.11-4.08)	0.653
**55 ≤ Age < 70 (N=4231)**			2999	1232		
Cardiovascular death	1.85 (1.56-2.18)	<0.001	385	194	1.26 (1.02-1.57)	0.033
Non-Cardiovascular death	1.68 (1.38-2.04)	<0.001	307	140	1.17 (0.91-1.51)	0.216
Valve related events						
Reoperation	6.19 (4.32-8.87)	<0.001	43	126	8.48 (5.58-12.87)	<0.001
Thromboembolism	1.05 (0.84-1.32)	0.675	266	107	1.02 (0.75-1.38)	0.916
Major bleeding	1.33 (0.98-1.80)	0.071	136	62	1.16 (0.77-1.74)	0.472
**Age ≥ 70 (N=1743)**			175	1568		
Cardiovascular death	0.96 (0.72-1.28)	0.794	43	427	1.13 (0.77-1.66)	0.524
Non-Cardiovascular death	1.03 (0.70-1.51)	0.894	28	235	0.95 (0.60-1.53)	0.843

© 2023 Park SJ et al. *JAMA Network Open.*

Valve related events						
Reoperation	Infinity	<0.001	0	54	Infinity	<0.001
Thromboembolism	1.84 (1.00-3.39)	0.051	9	157	2.23 (0.95-5.25)	0.067
Major bleeding	1.06 (0.48-2.33)	0.883	5	59	1.37 (0.52-3.61)	0.522

IPTW, inverse-probability-of-treatment weighting; aHR, adjusted hazard ratio; CI, confidence interval.

© 2023 Park SJ et al. *JAMA Network Open.*

eTable 20. Comparative Outcomes of Mechanical versus Bio-prosthetic DVR (double valve replacement) without competing-risk analysis

	Original		IPTW-adjusted			
	HR (95% CI)	P- value	Mechanical prosthesis	Biological prosthesis	aHR (95% CI)	P-value
**Age < 55 (N=1206)**			1163	43		
Cardiovascular death	3.01 (1.46-6.22)	0.003	86	6	2.00 (0.68-5.89)	0.207
Non-Cardiovascular death	2.91 (1.05-8.09)	0.041	49	1	0.93 (0.22-3.84)	0.917
Valve related events						
Reoperation	10.16 (4.63-22.31)	<0.001	31	6	5.77 (1.85-18.01)	0.003
Thromboembolism	1.62 (0.59-4.41)	0.348	88	3	1.05 (0.25-4.37)	0.945
Major bleeding	0.62 (0.09-4.51)	0.640	51	1	0.40 (0.05-3.08)	0.378
**55 ≤ Age < 65 (N=1130)**			996	134		
Cardiovascular death	1.77 (1.16-2.71)	0.008	121	31	2.13 (1.21-3.78)	0.009
Non-Cardiovascular death	2.95 (1.85-4.71)	<0.001	69	15	1.82 (0.94-3.54)	0.078
Valve related events						
Reoperation	6.81 (3.91-11.84)	<0.001	30	26	8.34 (4.57-15.2)	<0.001
Thromboembolism	0.32 (0.12-0.87)	0.025	99	4	0.29 (0.09-0.92)	0.035
Major bleeding	1.77 (0.92-3.39)	0.086	53	12	2.04 (0.91-4.57)	0.084
**Age ≥ 65 (N=1134)**			304	830		
Cardiovascular death	1.21 (0.93-1.59)	0.161	79	218	0.95 (0.64-1.41)	0.800
Non-Cardiovascular death	1.23 (0.86-1.75)	0.251	40	113	0.96 (0.59-1.55)	0.855

© 2023 Park SJ et al. *JAMA Network Open.*

Valve related events						
Reoperation	3.67 (1.45-9.29)	0.006	3	53	6.29 (2.14-18.46)	0.001
Thromboembolism	0.65 (0.42-0.99)	0.045	31	56	0.62 (0.35-1.09)	0.098
Major bleeding	0.74 (0.36-0.55)	0.429	6	19	1.20 (0.55-2.61)	0.655

IPTW, inverse-probability-of-treatment weighting; aHR, adjusted hazard ratio; CI, confidence interval.

© 2023 Park SJ et al. *JAMA Network Open.*

eTable 21. Adjusted hazards of bio-prosthesis for mortality according to various sub-groups in AVR (aortic valve replacement)

		Mechanical prosthesis	Biological prosthesis	*aHR (95% CI)	P value	P value for interaction
**Age < 55 (N=1804)**		N=1654	N=150			
Sex type	Male	1156	108	2.12 (1.20-3.74)	0.009	0.931
	Female	498	42	2.24 (0.78-6.42)	0.135	
Diabetes mellitus	No	1473	132	2.21 (1.23-3.96)	0.008	0.674
	Yes	181	19	1.76 (0.72-4.29)	0.214	
Prior Stroke	No	1566	136	2.03 (1.18-3.51)	0.011	0.864
	Yes	88	14	2.29 (0.65-7.99)	0.196	
Congestive heart failure	No	1273	117	2.21 (1.23-3.96)	0.008	0.962
	Yes	381	33	2.15 (0.81-5.70)	0.125	
Chronic kidney disease	No	1588	145	2.31 (1.34-3.99)	0.003	0.790
	Yes	66	5	2.67 (1.06-6.71)	0.037	
Atrial fibrillation	No	1605	146	2.29 (1.37-3.83)	0.002	0.460
	Yes	49	4	0.78 (0.05-12.9)	0.863	
Charlson comorbidity index	<2	1137	104	2.26 (1.11-4.58)	0.025	0.948
	≥2	517	46	2.18 (1.10-4.33)	0.026	
Dialysis	No	1591	142	2.40 (1.41-4.10)	0.001	0.122
	Yes	63	8	0.72 (0.17-3.00)	0.655	
**55 ≤ Age < 65 (N=3000)**		N=2227	N=773			
Sex type	Male	1426	484	1.40 (1.05-1.85)	0.020	0.342
	Female	801	289	1.09 (0.72-1.67)	0.682	
Diabetes mellitus	No	1714	596	1.38 (1.04-1.83)	0.028	0.392
	Yes	513	177	1.11 (0.73-1.67)	0.630	
Prior Stroke	No	2057	715	1.33 (1.04-1.71)	0.025	0.352
	Yes	170	58	0.94 (0.47-1.88)	0.855	
Congestive heart failure	No	1664	570	1.29 (0.97-1.70)	0.079	0.931
	Yes	563	203	1.26 (0.82-1.92)	0.288	
Chronic kidney disease	No	2113	731	1.32 (1.02-1.69)	0.032	0.781
	Yes	114	42	1.20 (0.64-2.24)	0.575	

© 2023 Park SJ et al. *JAMA Network Open.*

Atrial fibrillation	No	2109	732	1.29 (1.02-1.64)	0.036	0.891
	Yes	118	41	1.20 (0.41-3.46)	0.740	
Charlson comorbidity index	<2	1217	417	1.41 (0.97-2.04)	0.069	0.505
	≥2	1010	357	1.20 (0.88-1.63)	0.248	
Dialysis	No	2142	739	1.31 (1.02-1.68)	0.036	0.469
	Yes	85	35	1.02 (0.54-1.91)	0.956	
Concomitant coronary bypass	No	1991	695	1.29 (1.00-1.68)	0.054	0.969
	Yes	236	78	1.28 (0.74-2.22)	0.381	
**Age ≥ 65 (N=7189)**		N=944	N=6245			
Sex type	Male	523	3406	0.83 (0.68-1.00)	0.046	0.354
	Female	421	2839	0.71 (0.55-0.92)	0.009	
Diabetes mellitus	No	651	4240	0.81 (0.67-0.97)	0.022	0.413
	Yes	293	2005	0.70 (0.53-0.93)	0.015	
Prior Stroke	No	813	5374	0.81 (0.68-0.96)	0.013	0.236
	Yes	131	871	0.63 (0.43-0.91)	0.015	
Congestive heart failure	No	624	4212	0.77 (0.63-0.93)	0.007	0.792
	Yes	321	2033	0.80 (0.62-1.03)	0.081	
Chronic kidney disease	No	892	5920	0.79 (0.67-0.92)	0.003	0.348
	Yes	53	325	0.60 (0.35-1.02)	0.061	
Atrial fibrillation	No	859	5794	0.77 (0.65-0.90)	0.001	0.609
	Yes	85	451	0.89 (0.51-1.55)	0.683	
Charlson comorbidity index	<2	349	2393	0.86 (0.67-1.12)	0.264	0.371
	≥2	596	3853	0.75 (0.62-0.90)	0.003	
Dialysis	No	905	6072	0.80 (0.69-0.94)	0.006	0.201
	Yes	40	174	0.54 (0.29-0.98)	0.042	

*Analyzed in the inverse-probability-of-treatment weighting-adjusted cohort in each age-strata.

aHR, adjusted hazard ratio.

© 2023 Park SJ et al. *JAMA Network Open.*

eTable 22. Adjusted hazards of bio-prosthesis for mortality according to various sub-groups in MVR (mitral valve replacement)

		Mechanical prosthesis	Biological prosthesis	*aHR (95% CI)	P value	P value for interaction
**Age < 55 (N=2937)**		N=2783	N=154			
Sex type	Male	1225	67	0.92 (0.39-2.18)	0.846	0.412
	Female	1558	87	1.47 (0.71-3.07)	0.301	
Diabetes mellitus	No	2551	143	1.17 (0.60-2.27)	0.642	0.775
	Yes	232	11	1.39 (0.51-3.79)	0.515	
Prior Stroke	No	2450	128	1.22 (0.66-2.25)	0.527	0.550
	Yes	333	26	0.78 (0.21-2.92)	0.717	
Congestive heart failure	No	1876	114	1.48 (0.78-2.82)	0.230	0.084
	Yes	907	40	0.53 (0.20-1.41)	0.202	
Chronic kidney disease	No	2717	150	1.08 (0.57-2.05)	0.815	0.419
	Yes	66	4	1.89 (0.57-6.26)	0.298	
Atrial fibrillation	No	1940	114	1.48 (0.82-2.65)	0.191	0.042
	Yes	843	40	0.17 (0.02-1.26)	0.083	
Charlson comorbidity index	<2	1787	93	1.41 (0.57-3.48)	0.453	0.378
	≥2	996	61	0.86 (0.45-1.64)	0.642	
Dialysis	No	2743	151	1.17 (0.64-2.14)	0.609	0.421
	Yes	40	3	0.70 (0.24-2.10)	0.527	
**55 ≤ Age < 70 (N=4231)**		N=2999	N=1232			
Sex type	Male	1131	459	1.32 (1.03-1.68)	0.027	0.439
	Female	1868	773	1.16 (0.93-1.44)	0.182	
Diabetes mellitus	No	2390	953	1.31 (1.08-1.58)	0.006	0.094
	Yes	609	279	0.94 (0.68-1.31)	0.731	
Prior Stroke	No	2506	1020	1.28 (1.07-1.53)	0.008	0.265
	Yes	493	212	0.99 (0.66-1.49)	0.971	
Congestive heart failure	No	1608	639	1.26 (1.00-1.58)	0.046	0.641
	Yes	1392	593	1.17 (0.93-1.47)	0.193	
Chronic kidney disease	No	2916	1186	1.21 (1.02-1.43)-	0.025	0.562
	Yes	83	46	0.94 (0.41-2.18)	0.885	

© 2023 Park SJ et al. *JAMA Network Open.*

Atrial fibrillation	No	1741	739	1.25 (1.02-1.53)	0.033	0.756
	Yes	1258	493	1.18 (0.90-1.55)	0.224	
Charlson comorbidity index	<2	1364	527	1.49 (1.16-1.92)	0.002	0.035
	≥2	1635	705	1.05 (0.85-1.29)	0.682	
Dialysis	No	2950	1205	1.19 (1.01-1.41)	0.038	0.144
	Yes	49	27	1.87 (1.05-3.31)	0.033	
Concomitant coronary bypass	No	2798	1151	1.31 (1.10-1.56)	0.003	0.008
	Yes	201	82	0.69 (0.45-1.07)	0.099	
**Age ≥ 70 (N=1743)**		N=175	N=1568			
Sex type	Male	51	481	0.98 (0.62-1.54)	0.936	0.704
	Female	125	1087	1.10 (0.76-1.60)	0.617	
Diabetes mellitus	No	142	1243	1.05 (0.74-1.49)	0.795	0.836
	Yes	33	325	1.12 (0.68-1.83)	0.661	
Prior Stroke	No	144	1270	1.04 (0.75-1.44)	0.815	0.756
	Yes	31	298	1.17 (0.61-2.22)	0.641	
Congestive heart failure	No	77	702	0.68 (0.47-0.98)	0.038	0.001
	Yes	98	866	1.77 (1.16-2.70)	0.008	
Chronic kidney disease	No	169	1493	1.06 (0.78-1.43)	0.721	0.477
	Yes	6	75	0.80 (0.40-1.61)	0.535	
Atrial fibrillation	No	104	855	1.16 (0.77-1.74)	0.483	0.443
	Yes	71	713	0.93 (0.64-1.36)	0.708	
Charlson comorbidity index	<2	47	480	0.77 (0.45-1.29)	0.318	0.136
	≥2	128	1089	1.24 (0.87-1.75)	0.235	
Dialysis	No	174	1541	1.05 (0.78-1.41)	0.742	0.558
	Yes	1	27	0.91 (0.61-1.34)	0.622	

*Analyzed in the inverse-probability-of-treatment weighting-adjusted cohort in each age-strata.

aHR, adjusted hazard ratio.

© 2023 Park SJ et al. *JAMA Network Open.*

eTable 23. Baseline and operative characteristics of AVR (aortic valve replacement) patients aged 40 to 64 years

	Unadjusted				IPTW-adjusted		
	Mechanical prosthesis (n = 3881)	Biological prosthesis (n = 923)	p-value	SMD	Mechanical prosthesis (n = 3881)	Biological prosthesis (n = 923)	SMD
Baseline Demographics							
Age, years	54.9±6.4	59.4±5.3	<0.001	0.769	55.7±6.4	56.1±6.3	0.057
Female	1296 (33.4)	327 (35.4)	0.24	0.043	1308.9 (33.7)	300.2 (32.5)	0.026
Baseline Comorbidities							
Atrial fibrillation	179 (4.6)	33 (3.6)	0.168	0.052	171.7 (4.4)	37.8 (4.1)	0.016
Hypertension	1918 (49.4)	540 (58.5)	<0.001	0.183	1975.7 (50.9)	443.7 (48.1)	0.057
Diabetes mellitus	678 (17.5)	212 (23.0)	<0.001	0.137	716 (18.4)	174.9 (18.9)	0.013
Dyslipidemia	575 (14.8)	190 (20.6)	<0.001	0.152	626.2 (16.1)	144.8 (15.7)	0.012
Chronic kidney disease	156 (4.0)	75 (8.1)	<0.001	0.173	184 (4.7)	45.2 (4.9)	0.007
Dialysis	124 (3.2)	63 (6.8)	<0.001	0.167	150 (3.9)	41 (4.4)	0.029
Stroke, TIA or SE	260 (6.7)	62 (6.7)	0.984	0.001	262.2 (6.8)	68.2 (7.4)	0.025
Ischemic heart disease	1197 (30.8)	307 (33.3)	0.154	0.052	1210.1 (31.2)	263 (28.5)	0.059
Myocardial infarction	96 (2.5)	29 (3.1)	0.252	0.04	100.8 (2.6)	18.2 (2.0)	0.042
Previous PCI	116 (3.0)	41 (4.4)	0.026	0.077	129.6 (3.3)	25.6 (2.8)	0.033
Congestive heart failure	915 (23.6)	249 (27.0)	0.03	0.078	945.7 (24.4)	228 (24.7)	0.008
Anemia	340 (8.8)	123 (13.3)	<0.001	0.146	376 (9.7)	98 (10.6)	0.031
COPD	104 (2.7)	35 (3.8)	0.07	0.063	114.1 (2.9)	24.2 (2.6)	0.019
Asthma	365 (9.4)	107 (11.6)	0.045	0.071	385.3 (9.9)	99.4 (10.8)	0.028
Peripheral vascular disease	177 (4.6)	58 (6.3)	0.029	0.076	188.1 (4.8)	35.7 (3.9)	0.048
Previous cardiac surgery	15 (0.4)	1 (0.1)	0.336	0.056	12.4 (0.3)	0.3 (0.0)	0.069
Previous cancer	154 (4.0)	82 (8.9)	<0.001	0.201	191.8 (4.9)	51.6 (5.6)	0.029

© 2023 Park SJ et al. *JAMA Network Open*

Charlson comorbidity index			<0.001	0.27			0.035
0	1365 (35.2)	236 (25.6)		0.27	1296 (33.4)	303.2 (32.8)	
1	1047 (27.0)	226 (24.5)			1030.8 (26.6)	248.2 (26.9)	
2	643 (16.6)	178 (19.3)			653 (16.8)	149.7 (16.2)	
≥ 3	547 (14.1)	180 (19.5)			591.1 (15.2)	140.3 (15.2)	
≥ 5	279 (7.2)	103 (11.2)			310.1 (8.0)	81.6 (8.8)	
Years of Surgery			<0.001	0.398			0.093
2002~2005	828 (21.3)	113 (12.2)			756.4 (19.5)	152.4 (16.5)	
2006~2009	911 (23.5)	174 (18.9)			875.6 (22.6)	197.1 (21.4)	
2010~2013	999 (25.7)	197 (21.3)			964.1 (24.8)	242.7 (26.3)	
2014~2018	1143 (29.5)	439 (47.6)			1284.9 (33.1)	330.7 (35.8)	
Level of hospital			<0.001	0.154			0.019
*Tertiary general hospital	3125 (80.5)	684 (74.1)			3077.3 (79.3)	724.7 (78.5)	
General hospital	756 (19.5)	239 (25.9)			804 (20.7)	198.3 (21.5)	
Cumulative hospital volume for cardiac surgery			<0.001	0.169			0.095
<250	975 (25.1)	228 (24.7)			984.3 (25.4)	252 (27.3)	
250-999	1034 (26.6)	301 (32.6)			1089 (28.1)	283.2 (30.7)	
1000-2999	1108 (28.5)	261 (28.3)			1088.7 (28.1)	239.4 (25.9)	
≥3000	764 (19.7)	133 (14.4)			719.1 (18.5)	148.3 (16.1)	
Endocarditis	600 (15.5)	159 (17.2)	0.186	0.048	615.1 (15.8)	156.4 (16.9)	0.029
Congestive heart failure	960 (24.7)	248 (26.9)	0.179	0.049	982.1 (25.3)	248.1 (26.9)	0.036
Bicuspid aortic valve	546 (14.1)	130 (14.1)	0.99	<0.001	545.8 (14.1)	147 (15.9)	0.052
Mode of valve disease			<0.001	0.192			0.032
Aortic stenosis	1083 (27.9)	330 (35.8)			1149.7 (29.6)	280.4 (30.4)	
Aortic regurgitation	1248 (32.2)	236 (25.6)			1199.3 (30.9)	291.5 (31.6)	
Combined	1430 (36.8)	323 (35.0)			1406.4 (36.2)	320.5 (34.7)	
Unspecified	120 (3.1)	34 (3.7)			125.6 (3.2)	30.5 (3.3)	

© 2023 Park SJ et al. *JAMA Network Open*

Concomitant Procedure							
Tricuspid valve repair	115 (3.0)	15 (1.6)	0.024	0.089	104.5 (2.7)	22.9 (2.5)	0.013
Coronary arterial bypass grating	317 (8.2)	99 (10.7)	0.013	0.088	332.3 (8.6)	72.2 (7.8)	0.027
Surgical ablation for atrial fibrillation	152 (3.9)	29 (3.1)	0.267	0.042	147.9 (3.8)	43.2 (4.7)	0.043
Health Screening Data							
Height, m	163.6±8.6	162.1±8.3	<0.001	0.172	163.4±8.5	163.0±8.22	0.045
Weight, kg	65.6±11.2	64.2±10.9	0.004	0.128	65.3±11.2	64.8±11.4	0.043
BMI, kg/m^2	24.4±3.2	24.4±3.4	0.667	0.019	24.4±3.3	24.3±3.4	0.019
< 18.5	38 (1.0)	22 (2.4)	0.003	0.142	54 (1.4)	13.3 (1.4)	0.075
≥ 18.5 and < 23	835 (21.5)	196 (21.2)			836.8 (21.6)	222.2 (24.1)	
≥ 23 and < 25	647 (16.7)	168 (18.2)			661.3 (17.0)	153.1 (16.6)	
≥ 25 and < 30	911 (23.5)	233 (25.2)			912.9 (23.5)	198.9 (21.5)	
≥ 30	137 (3.5)	30 (3.3)			136.5 (3.5)	37.6 (4.1)	
Not available	1313 (33.8)	274 (29.7)			1279.4 (33.0)	298 (32.3)	
Systolic blood pressure, mmHg			0.052	0.102			0.027
< 120	849 (21.9)	206 (22.3)			852 (22.0)	206.9 (22.4)	
≥ 120 and < 140	1218 (31.4)	301 (32.6)			1232.8 (31.8)	300.9 (32.6)	
≥ 140	501 (12.9)	142 (15.4)			516.8 (13.3)	117.2 (12.7)	
Not available	1313 (33.8)	274 (29.7)			1279.4 (33.0)	298 (32.3)	
Diastolic blood pressure, mmHg			0.096	0.093			0.036
< 80	1403 (36.2)	363 (39.3)			1429.4 (36.8)	348 (37.7)	
≥ 80 and < 90	857 (22.1)	214 (23.2)			870.6 (22.4)	212.3 (23.0)	
≥ 90	308 (7.9)	72 (7.8)			301.6 (7.8)	64.7 (7.0)	
Not available	1313 (33.8)	274 (29.7)			1279.4 (33.0)	298 (32.3)	
Smoking			0.092	0.094			0.019
Never smoker	1358 (35.0)	356 (38.6)			1380 (35.6)	335 (36.3)	
Previous smoker	567 (14.6)	137 (14.8)			566.8 (14.6)	134.4 (14.6)	

© 2023 Park SJ et al. *JAMA Network Open*

Current smoker	620 (16.0)	150 (16.3)			631.7 (16.3)	151.1 (16.4)	
Not available	1336 (34.4)	280 (30.3)			1302.5 (33.6)	302.5 (32.8)	
Alcohol use			<0.001	0.200			0.056
None	1025 (26.4)	320 (34.7)			1082.9 (27.9)	279.8 (30.3)	
Mild-to-moderate	1372 (35.4)	277 (30.0)			1334.2 (34.4)	301.1 (32.6)	
Heavy	154 (4.0)	48 (5.2)			167.9 (4.3)	41 (4.4)	
Not available	1330 (34.3)	278 (30.1)			1296 (33.4)	301.1 (32.6)	
Creatinine, mg/dL			<0.001	0.224			0.07
<= 1.5	1702 (43.9)	497 (53.8)			1774.6 (45.7)	447 (48.4)	
> 1.5	67 (1.7)	25 (2.7)			74.4 (1.9)	22.6 (2.4)	
Not available	2112 (54.4)	401 (43.4)			2032 (52.4)	453.4 (49.1)	
eGFR, mL/min/1.73m^2			<0.001	0.25			0.052
≥ 60	1287 (33.2)	389 (42.1)			1359.7 (35.0)	343.4 (37.2)	
< 60	136 (3.5)	58 (6.3)			158.4 (4.1)	41.1 (4.5)	
Not available	2458 (63.3)	476 (51.6)			2362.9 (60.9)	538.4 (58.3)	

*Designated and certificated by the Ministry of Health and Welfare

Values are n (%), or mean ± standard deviation, unless otherwise indicated.
IPTW, inverse-probability-of-treatment weighting; SMD, standardized mean difference; BMI, body mass index; TIA, transient ischemic attack; SE, systemic embolization; PCI, percutaneous coronary intervention; COPD, chronic obstructive pulmonary disease; eGFR, estimated glomerular filtration rate; AVR, aortic valve replacement; MVR, mitral valve replacement.

© 2023 Park SJ et al. *JAMA Network Open*

eTable 24. Baseline and operative characteristics of MVR (mitral valve replacement) patients aged 40 to 69 years

	Unadjusted				IPTW-adjusted		
	Mechanical prosthesis (n = 5782)	Biological prosthesis (n = 1386)	p-value	SMD	Mechanical prosthesis (n = 5782)	Biological prosthesis (n = 1386)	SMD
Baseline Demographics							
Age, years	54.7±7.5	63.0±6.4	<0.001	1.19	56.3±8.0	57.5±7.8	0.156
Female	3400 (58.8)	886 (63.9)	<0.001	0.105	3447.7 (59.6)	829.9 (59.9)	0.005
Baseline conditions							
Atrial fibrillation	2158 (37.3)	493 (35.6)	0.225	0.036	2153.1 (37.2)	510 (36.8)	0.009
Hypertension	2780 (48.1)	811 (58.5)	<0.001	0.21	2904.8 (50.2)	726 (52.4)	0.043
Diabetes mellitus	765 (13.2)	323 (23.3)	<0.001	0.263	896.5 (15.5)	247.2 (17.8)	0.063
Dyslipidemia	695 (12.0)	200 (14.4)	0.015	0.071	725.2 (12.5)	185.8 (13.4)	0.026
Chronic kidney disease	119 (2.1)	72 (5.2)	<0.001	0.168	156.6 (2.7)	53.8 (3.9)	0.066
Dialysis	65 (1.1)	51 (3.7)	<0.001	0.167	94.6 (1.6)	31.5 (2.3)	0.046
Stroke, TIA or SE	812 (14.0)	228 (16.5)	0.022	0.067	843.4 (14.6)	217.3 (15.7)	0.03
Ischemic heart disease	1147 (19.8)	368 (26.6)	<0.001	0.16	1208.4 (20.9)	299.6 (21.6)	0.017
Myocardial infarction	140 (2.4)	48 (3.5)	0.029	0.062	156.7 (2.7)	37.7 (2.7)	<0.001
Previous PCI	84 (1.5)	43 (3.1)	<0.001	0.111	100.4 (1.7)	21.3 (1.5)	0.016
Congestive heart failure	2264 (39.15)	658 (47.5)	<0.001	0.168	2355.7 (40.7)	581.4 (41.9)	0.024
Anemia	462 (8.0)	181 (13.1)	<0.001	0.166	529.8 (9.2)	136.1 (9.8)	0.022
COPD	164 (2.8)	78 (5.6)	<0.001	0.139	191.5 (3.3)	47.2 (3.4)	0.005
Asthma	776 (13.4)	253 (18.3)	<0.001	0.133	826.6 (14.3)	215.1 (15.5)	0.034
Peripheral vascular disease	245 (4.2)	87 (6.3)	0.001	0.091	273.6 (4.7)	70.5 (5.1)	0.016
Previous cardiac surgery	21 (0.4)	8 (0.6)	0.26	0.031	21.6 (0.4)	4.1 (0.3)	0.013
Previous cancer	193 (3.3)	78 (5.6)	<0.001	0.111	220.5 (3.8)	65.6 (4.7)	0.046

© 2023 Park SJ et al. *JAMA Network Open*

Charlson comorbidity index			<0.001	0.398			0.127
0	1590 (27.5)	215 (15.5)			1451.2 (25.1)	289.1 (20.9)	
1	1675 (29.0)	346 (25.0)			1617.6 (28.0)	374.4 (27.0)	
2	1152 (19.9)	311 (22.4)			1181.3 (20.4)	305.9 (22.1)	
≥ 3	998 (17.3)	315 (22.7)			1066.8 (18.5)	273 (19.7)	
≥ 5	367 (6.3)	199 (14.4)			465.1 (8.0)	143.6 (10.4)	
Years of Surgery			<0.001	0.148			0.101
2002~2005	1565 (27.1)	302 (21.8)			1497.1 (25.9)	302.6 (21.8)	
2006~2009	1419 (24.5)	386 (27.8)			1452.6 (25.1)	367 (26.5)	
2010~2013	1286 (22.2)	285 (20.6)			1265.4 (21.9)	304.1 (21.9)	
2014~2018	1512 (26.2)	413 (29.8)			1566.8 (27.1)	412.3 (29.7)	
Level of hospital			<0.001	0.125			0.002
*Tertiary general hospital	4433 (76.7)	987 (71.2)			4393.4 (76.0)	1051.9 (75.9)	
General hospital	1349 (23.3)	399 (28.8)			1388.6 (24.0)	334.1 (24.1)	
Cumulative hospital volume for cardiac surgery			<0.001	0.313			0.228
<250 cases	1985 (34.3)	544 (39.2)			2028 (35.1)	486.8 (35.1)	
250-999 cases	1239 (21.4)	427 (30.8)			1371.9 (23.7)	453.8 (32.7)	
1000-2999 cases	2558 (44.2)	415 (29.9)			2382.1 (41.2)	445.4 (32.1)	
Endocarditis	747 (12.9)	255 (18.4)	<0.001	0.151	805.1 (13.9)	224.8 (16.2)	0.064
Congestive heart failure	2057 (35.6)	571 (41.2)	<0.001	0.116	2122.3 (36.7)	544.6 (39.3)	0.053
Mode of valve disease			<0.001	0.178			0.074
Mitral stenosis	3033 (52.5)	700 (50.5)			3015.4 (52.2)	708.7 (51.1)	
Mitral regurgitation	174 (3.0)	70 (5.1)			180.3 (3.1)	56.2 (4.1)	
Combined	2353 (40.7)	520 (37.5)			2336.7 (40.4)	545.9 (40.0)	
Unspecified	222 (3.8)	96 (6.9)			249.5 (4.3)	75.2 (5.4)	
Concomitant Procedure							
Tricuspid valve repair	2372 (41.0)	573 (41.3)	0.829	0.006	2347.4 (40.6)	527.2 (38.0)	0.052

© 2023 Park SJ et al. *JAMA Network Open*

Coronary arterial bypass grating	251 (4.3)	105 (7.6)	<0.001	0.137	290.9 (5.0)	75.6 (5.5)	0.019
Surgical ablation for atrial fibrillation	2570 (44.4)	612 (44.2)	0.844	0.006	2545.7 (44.0)	618.2 (44.6)	0.012
Health Screening Data							
Height, m	160.8±8.7	158.1±8.3	<0.001	0.318	160.4±8.7	159.7±8.8	0.083
Weight, kg	61.2±10.8	58.5±10.1	<0.001	0.257	60.8±10.7	60.2±10.6	0.052
BMI, kg/m^2	23.6±3.2	23.4±3.3	0.056	0.074	23.6±3.2	23.6±3.3	0.001
< 18.5	119 (2.05)	40 (2.9)	0.148	0.084	133.4 (2.3)	31.8 (2.3)	0.017
≥ 18.5 and < 23	1379 (23.84)	352 (25.4)			1395.6 (24.1)	339.4 (24.5)	
≥ 23 and < 25	810 (14.00)	210 (15.2)			812.2 (14.0)	197.3 (14.2)	
≥ 25 and < 30	936 (16.18)	205 (14.8)			916 (15.8)	214.9 (15.5)	
≥ 30	113 (1.95)	27 (1.9)			115.8 (2.0)	30 (2.2)	
Not available	2425 (41.94)	552 (39.8)			2409 (41.7)	572.7 (41.3)	
Systolic blood pressure, mmHg			<0.001	0.161			0.043
< 120	1678 (29.02)	343 (24.7)			1623.7 (28.1)	377.9 (27.3)	
≥ 120 and < 140	1326 (22.93)	359 (25.9)			1347.2 (23.3)	323.7 (23.4)	
≥ 140	353 (6.10)	131 (9.5)			402.2 (7.0)	111.3 (8.0)	
Not available	2425 (41.94)	553 (39.9)			2409 (41.7)	573.2 (41.4)	
Diastolic blood pressure, mmHg			0.008	0.1			0.037
< 80	2128 (36.8)	485 (35.0)			2095.8 (36.2)	486.9 (35.1)	
≥ 80 and < 90	909 (15.7)	244 (17.6)			931.7 (16.1)	234.3 (16.9)	
≥ 90	320 (5.5)	104 (7.5)			345.5 (6.0)	91.6 (6.6)	
Not available	2425 (41.9)	553 (39.9)			2409 (41.7)	573.2 (41.4)	
Smoking			0.004	0.110			0.052
Never smoker	2307 (39.9)	612 (44.2)			2352.3 (40.7)	585.9 (42.3)	
Previous smoker	443 (7.7)	86 (6.2)			425 (7.4)	85.2 (6.1)	
Current smoker	534 (9.2)	101 (7.3)			508.8 (8.8)	122.3 (8.8)	

© 2023 Park SJ et al. *JAMA Network Open*

Not available	2498 (43.2)	587 (42.4)			2495.8 (43.2)	592.7 (42.8)	
Alcohol use			0.001	0.117			0.011
None	1496 (25.9)	420 (30.3)			1533.3 (26.5)	364.2 (26.3)	0.011
Mild-to-moderate	1682 (29.1)	346 (25.0)			1641.4 (28.4)	400.1 (28.9)	
Heavy	113 (2.0)	31 (2.2)			115.9 (2.0)	28.5 (2.1)	
Not available	2491 (43.1)	589 (42.5)			2491.3 (43.1)	593.2 (42.8)	
Creatinine, mg/dL			<0.001	0.137			0.031
<= 1.5	2121 (36.7)	514 (37.1)			2118.2 (36.6)	501 (36.1)	
> 1.5	54 (0.9)	38 (2.7)			71.5 (1.2)	22 (1.6)	
Not available	3607 (62.4)	834 (60.2)			3592.3 (62.1)	862.9 (62.3)	
eGFR, mL/min/1.73m^2			<0.001	0.214			0.054
≥ 60	1490 (25.8)	329 (23.7)			1477.3 (25.5)	345.1 (24.9)	
< 60	209 (3.6)	121 (8.7)			254.8 (4.4)	77.2 (5.6)	
Not available	4083 (70.6)	936 (67.5)			4049.9 (70.0)	963.7 (69.5)	

*Designated and certificated by the Ministry of Health and Welfare

Values are n (%), or mean ± standard deviation, unless otherwise indicated.
IPTW, inverse-probability-of-treatment weighting; SMD, standardized mean difference; BMI, body mass index; TIA, transient ischemic attack; SE, systemic embolization; PCI, percutaneous coronary intervention; COPD, chronic obstructive pulmonary disease; eGFR, estimated glomerular filtration rate; AVR, aortic valve replacement; MVR, mitral valve replacement.

© 2023 Park SJ et al. *JAMA Network Open*

eTable 25. Comparative Outcomes of Mechanical versus Biologic Prosthesis in Patients Aged < 65 years in AVR (aortic valve replacement), < 70 years in MVR (mitral valve replacement), and 65 years in DVR (double valve replacement) using Competing-risk Analysis.

	Original		IPTW-adjusted			
	HR (95% CI)	P- value	Mechanical prosthesis	Biological prosthesis	aHR (95% CI)	P-value
**AVR, Age < 65 (N=4804)**			3881	923		
Death	1.90 (1.60-2.26)	<0.001	518	175	1.60 (1.28-2.01)	<0.001
Cardiovascular death	2.06 (1.62-2.61)	<0.001	248	100	1.86 (1.37-2.51)	<0.001
Non-Cardiovascular death	1.55 (1.20-2.00)	0.001	269	74	1.26 (0.89-1.78)	0.19
Valve related events						
Reoperation	2.83 (1.90-4.23)	<0.001	12	4	3.30 (2.05-5.31)	<0.001
Thromboembolism	0.71 (0.52-0.99)	0.042	292	36	0.54 (0.36-0.81)	0.003
Major bleeding	0.93 (0.58-1.50)	0.77	111	20	0.81 (0.45-1.44)	0.47
**MVR, Age < 70 (N=7168)**			5782	1386		
Death	2.32 (2.07-2.61)	<0.001	1039	313	1.36 (1.16-1.61)	<0.001
Cardiovascular death	2.16 (1.85-2.51)	<0.001	578	157	1.18 (0.95-1.47)	0.13
Non-Cardiovascular death	2.16 (1.81-2.57)	<0.001	461	156	1.52 (1.19-1.94)	<0.001
Valve related events						
Reoperation	4.88 (3.74-6.36)	<0.001	102	152	7.01 (5.11-9.60)	<0.001
Thromboembolism	1.03 (0.83-1.26)	0.82	470	105	0.97 (0.72-1.32)	0.85

© 2023 Park SJ et al. *JAMA Network Open*

Major bleeding	1.24 (0.94-1.63)	0.13	241	52	0.94 (0.62-1.43)	0.79
**DVR, Age < 65 (N=)**						
Death						
Cardiovascular death						
Non-Cardiovascular death						
Valve related events						
Reoperation						
Thromboembolism						
Major bleeding						

IPTW, inverse-probability-of-treatment weighting; aHR, adjusted hazard ratio; CI, confidence interval.

© 2023 Park SJ et al. *JAMA Network Open*

eFigure 1. Patient Inclusion Flow Diagram

AVR, aortic valve replacement; ECMO, extracorporeal membrane oxygenation; IABP, intra-aortic balloon pump; MVR, mitral valve replacement; NHIS, National Health Insurance Service; PVR, pulmonary valve replacement; TVR, tricuspid valve replacement.

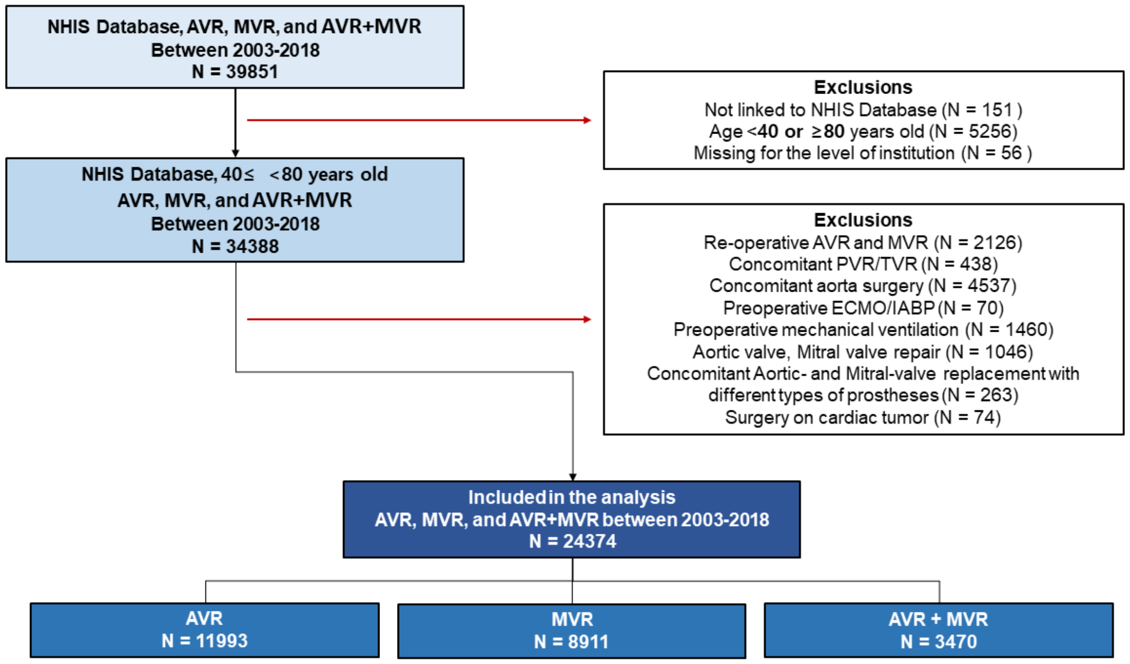

© 2023 Park SJ et al. *JAMA Network Open*

eFigure 2. Distributions of patients undergoing valve replacements depending on age strata.

In (A) aortic valve replacement, (B) mitral valve replacement, and (C) simultaneous aortic- and mitral-valve replacement.

AVR, aortic valve replacement; MVR, mitral valve replacement.

© 2023 Park SJ et al. *JAMA Network Open*

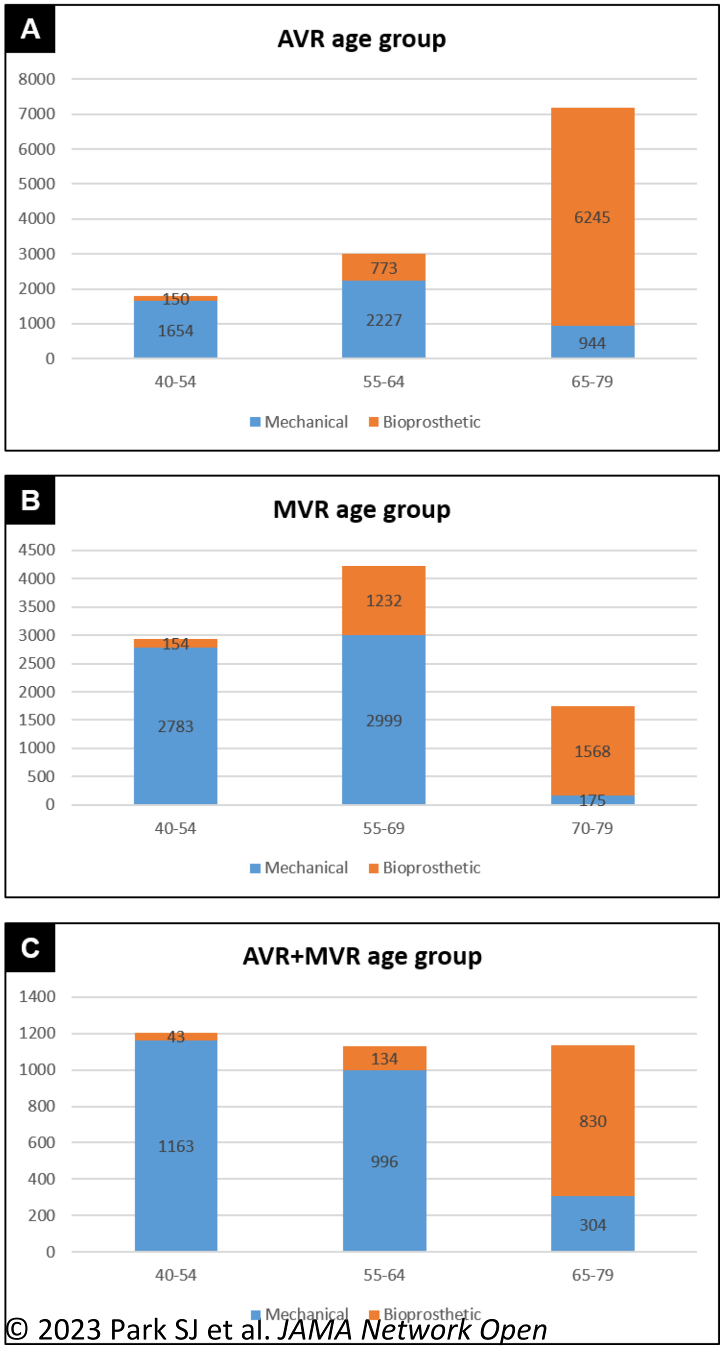

© 2023 Park SJ et al. *JAMA Network Open*

eFigure 3. Distributions of propensity scores according to age strata in (A) AVR, (B) MVR, and (C) DVR.

AVR, aortic valve replacement; MVR, mitral valve replacement; DVR, double valve replacement.

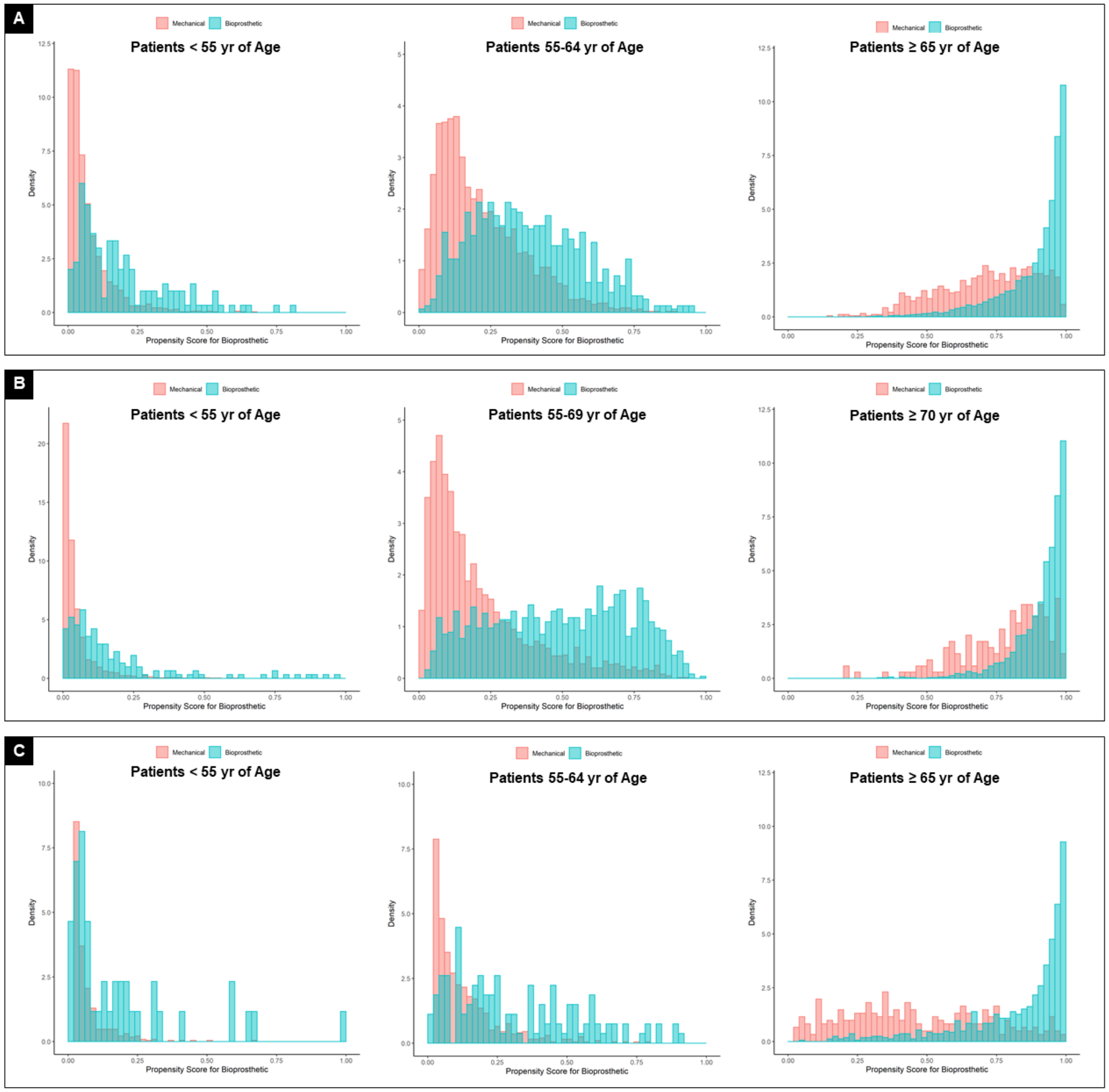

© 2023 Park SJ et al. *JAMA Network Open*

eFigure 4. The adjusted risks of mortality of bio-prosthesis in patients aged < 55 years in (A) AVR and (B) MVR.
AVR, aortic valve replacement; MVR, mitral valve replacement; aHR, adjusted hazard ratio; CI, confidence interval.

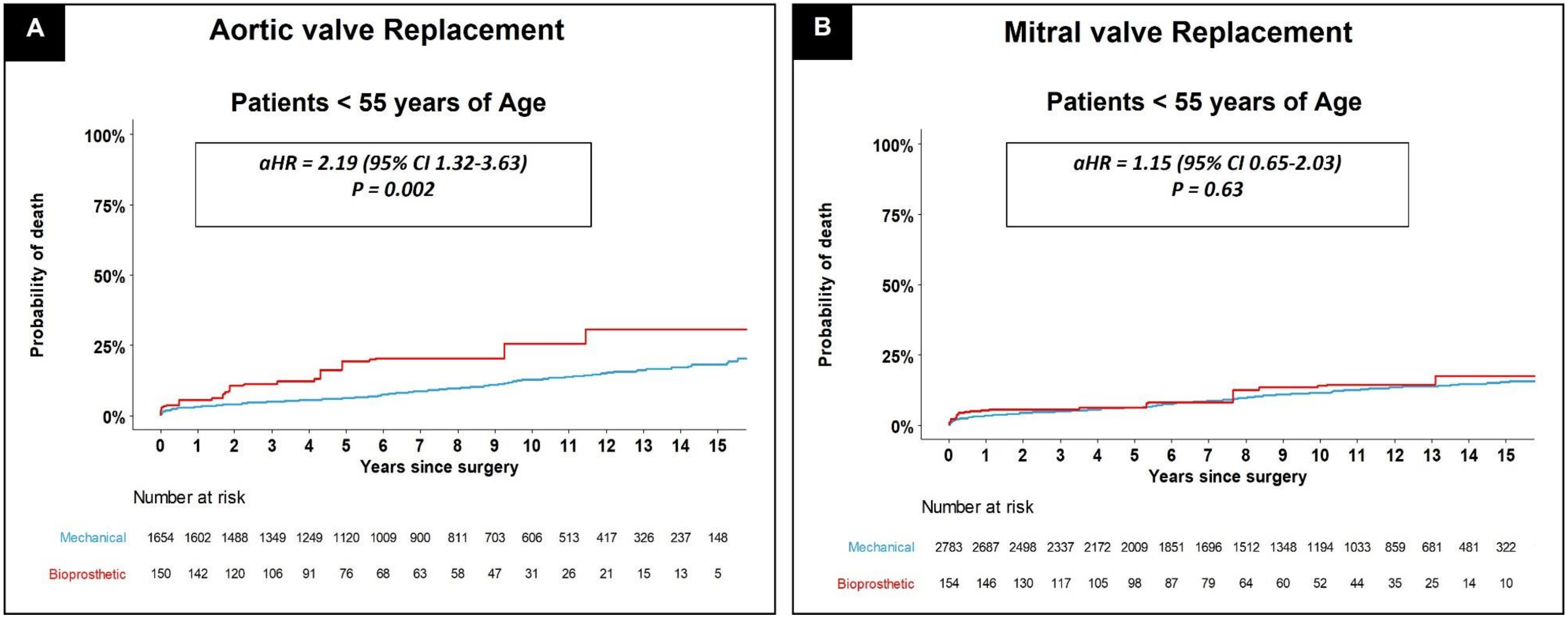

© 2023 Park SJ et al. *JAMA Network Open*

eFigure 5. The adjusted risks of mortality of bioprosthesis according to age strata in DVR.

DVR, double valve replacement; aHR, adjusted hazard ratio; CI, confidence interval

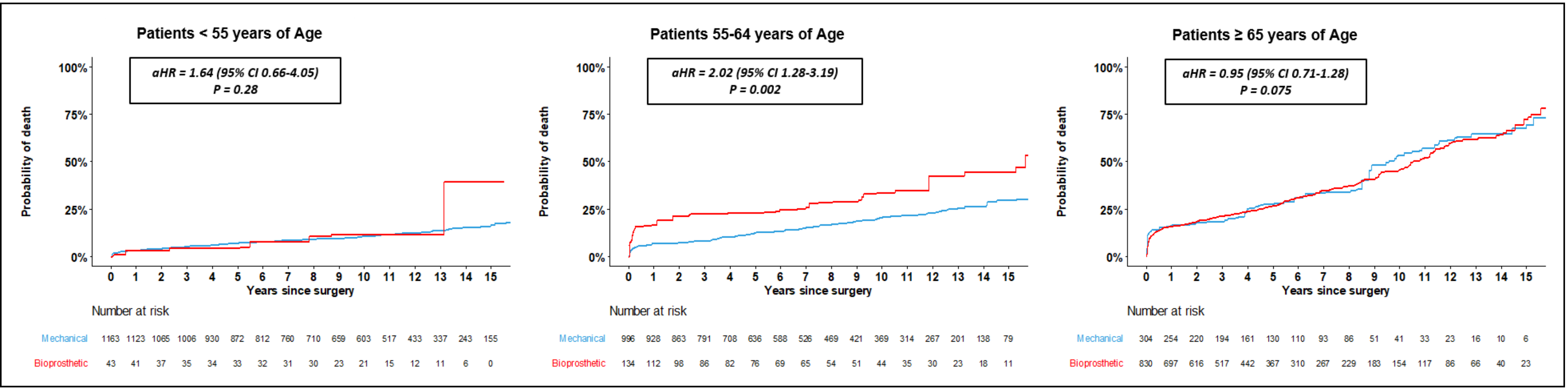

© 2023 Park SJ et al. *JAMA Network Open*

eFigure 6. Adjusted risks for mortality according to various subgroups in the middle age group.

(A) Aortic valve replacement in 55 to 64 years of age, and (B) Mitral valve replacement in 55 to 69 years of age

CHF, congestive heart failure; CKD, chronic kidney disease; DM, diabetes mellitus.

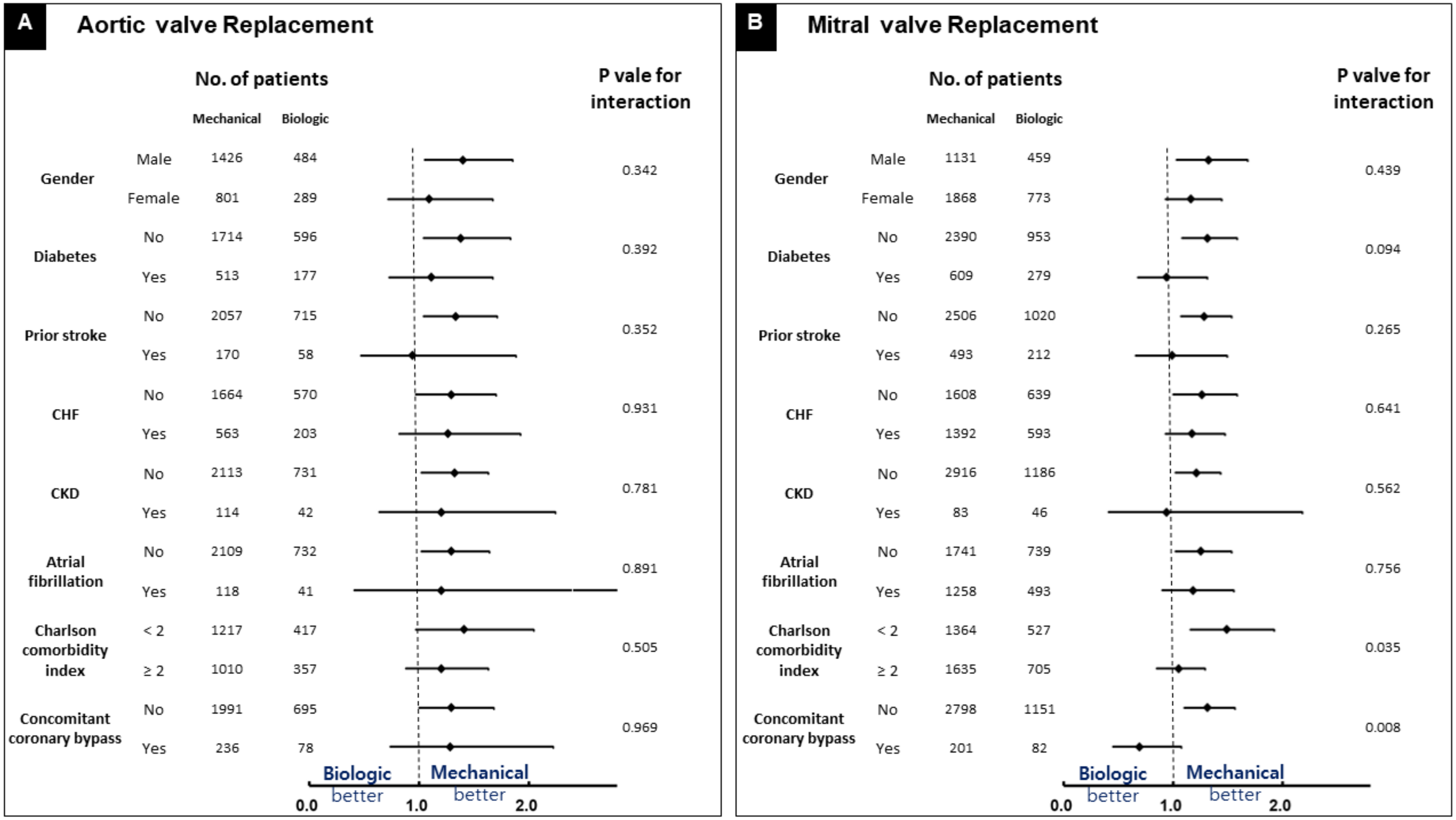

© 2023 Park SJ et al. *JAMA Network Open*
