# Supplementary material for: Evaluating Reference Ages for Selecting Prosthesis Types for Heart Valve Replacement in Korea
Source: JAMA Netw Open. 2023 May 22;6(5):e2314671. doi: 10.1001/jamanetworkopen.2023.14671 (PMC10203891; doi:10.1001/jamanetworkopen.2023.14671)
Supplement: Supplement 2. — Data Sharing Statement [file jamanetwopen-e2314671-s002.pdf]

Data Sharing Statement

Park. Evaluating Reference Ages for Selecting Prosthesis Types for Heart Valve Replacement in Korea. *JAMA Netw Open*. Published May 22, 2023.
doi:10.1001/jamanetworkopen.2023.14671

Data
Data available: No
